# Selenazolyl-hydrazones as Novel Selective MAO Inhibitors With Antiproliferative and Antioxidant Activities: Experimental and *In-silico* Studies

**DOI:** 10.3389/fchem.2018.00247

**Published:** 2018-07-03

**Authors:** Hana Elshaflu, Tamara R. Todorović, Milan Nikolić, Aleksandar Lolić, Aleksandar Višnjevac, Stefanie Hagenow, José M. Padrón, Alfonso T. García-Sosa, Ivana S. Djordjević, Sonja Grubišić, Holger Stark, Nenad R. Filipović

**Affiliations:** ^1^Faculty of Technology and Metallurgy, University of Belgrade, Belgrade, Serbia; ^2^Department of General and Inorganic Chemistry, University of Belgrade - Faculty of Chemistry, Belgrade, Serbia; ^3^Division of Physical Chemistry, Ruder Bošković Institute, Zagreb, Croatia; ^4^Institute of Pharmaceutical and Medicinal Chemistry, Heinrich Heine University Düsseldorf, Düsseldorf, Germany; ^5^Instituto Universitario de Bio-Orgánica “Antonio González”, Universidad de La Laguna, Tenerife, Spain; ^6^Institute of Chemistry, University of Tartu, Tartu, Estonia; ^7^Institute of Chemistry, Technology and Metallurgy, University of Belgrade, Belgrade, Serbia; ^8^Department of Chemistry and Biochemistry, Faculty of Agriculture, University of Belgrade, Belgrade, Serbia

**Keywords:** selenazoles, MAO B, Anticancer activity, Docking, Antioxidant agents

## Abstract

The novel approach in the treatment of complex multifactorial diseases, such as neurodegenerative disorders and cancer, requires a development of efficient multi-targeting oriented drugs. Since oxidative stress significantly contributes to the pathogenesis of cancer and neurodegenerative disorders, potential drug candidates should possess good antioxidant properties. Due to promising biological activities shown for structurally related (1,3-thiazol-2-yl)hydrazones, a focused library of 12 structurally related benzylidene-based (1,3-selenazol-2-yl)hydrazones was designed as potential multi-targeting compounds. Monoamine oxidases (MAO) A/B inhibition properties of this class of compounds have been investigated. Surprisingly, the *p*-nitrophenyl-substituted (1,3-selenazol-2-yl)hydrazone **4** showed MAO B inhibition in a nanomolar concentration range (IC_50_ = 73 nM). Excellent antioxidant properties were confirmed in a number of different *in vitro* assays. Antiproliferative activity screening on a panel of six human solid tumor cell lines showed that potencies of some of the investigated compounds was comparable or even better than that of the positive control 5-fluorouracil. *In-silico* calculations of ADME properties pointed to promising good pharmacokinetic profiles of investigated compounds. Docking studies suggest that some compounds, compared to positive controls, have the ability to strongly interact with targets relevant to cancer such as 5′-nucleotidase, and to neurodegenerative diseases such as the small conductance calcium-activated potassium channel protein 1, in addition to confirmation of inhibitory binding at MAO B.

## Introduction

After selenium was recognized as essential element to mammals, awareness about selenium toxicity changed in great extent and nowadays it is considered as micronutrient used in disease prevention and treatment by selenium supplementation (Weekley and Harris, 2013). Selenium chemistry is more similar to sulfur, than to lighter chalcogen element—oxygen. The most important difference among two heavier chalcogens, which is related to their redox chemistry, is known as “selenium paradox”—selenium's ability to undergo fast oxidation and then reversible reduction (Reich and Hondal, 2016). Also, almost all chemical reactions involving selenium are faster in comparison to the similar reactions with sulfur. For example, replacement of selenium with sulfur in selenium-based enzymes reduced catalytic activity, while opposite trend was found for Cys-containing enzymes after isosteric replacement of sulfur with selenium atom (Reich and Hondal, 2016).

Selenocysteine, selenium analog of cysteine, is the 21st amino acid incorporated in 25 Se-proteins encoded within human genome, while more than 30 Se-proteins have been identified in mammals (Cardoso et al., 2015). Numerous biological functions of selenium are expressed mainly *via* its role in catalytic reactions since it is constituent of active site of Se-proteins. Selenium is essential for the brain and participates in the pathology of neurodegenerative disorders, amyotrophic lateral sclerosis and epilepsy (Solovyev, 2015). Some Se-proteins are involved in processes such as thyroid hormones metabolism, spermatogenesis, and Se-proteins biosynthesis, while others participate in antioxidant defense and redox state regulation (Roman et al., 2014). Se-proteins with antioxidant function protect directly against oxidative stress or indirectly via regeneration and activation of low molecular weight antioxidants, when provided at low nutritional levels. On the other hand, elevated doses of selenium result in manifestation of its pro-oxidant, growth inhibition and cytotoxic properties (Fernandes and Gandin, 2015). Apart from usage of selenium compounds in diseases prevention by selenium supplementation, developing of synthetic organoselenium compounds as well as their metal complexes is subject of research in the field of medicinal chemistry. The potential of synthetic selenium compounds in medicinal chemistry include antioxidant, antitumor, antiviral, antimicrobial, anti-infective, anti-inflammatory, antiparasitic, antidiabetic, antimalarial, neuroprotective, antihypersensitive, and cardiotonic agents as well as enzyme inhibitors and immunomodulators (Karvekar et al., 2007; Akhoon et al., 2015; Filipović et al., 2016).

Selenium-containing heterocycles represent an interesting class of compounds because of both, interesting chemical properties and pharmaceutical applications (Mugesh et al., 2001). Based on the advantages related to the presence of selenium and the importance of heterocycles in the field of medicinal chemistry, synthesis of organoselenium compounds containing 1,3-selenazole ring, as well as study of their biological application, is in focus of current research. Many functionalized 1,3-selenazole rings are important constituent of pharmacologically active compounds (Zhao et al., 2013). 1,3-Selenazole derivatives are known to inhibit the synthesis of nitric acid (Ueda et al., 2005) and they act as antagonists for histamine H2 receptors (van der Goot et al., 1994). They also display anticancer (Zaharia et al., 2013; Zhao et al., 2013; Hong et al., 2015), antimicrobial (Al-Rubaie et al., 2014; Łaczkowski et al., 2016; Mbaveng et al., 2016; Filipović et al., 2017), and xantine oxidase inhibitory activities (Šmelcerović et al., 2017).

The biological activity (1,3-selenazol-2-yl)hydrazones is relatively unexplored area of research: only two studies dealing with anticancer (Zaharia et al., 2013; Zhao et al., 2013) and three studies dealing with antimicrobial activity (Łaczkowski et al., 2016; Mbaveng et al., 2016; Filipović et al., 2017) of (1,3-selenazol-2-yl)hydrazones have been published up to now. Despite the fact that (1,3-selenazol-2-yl)hydrazones are structurally related to their sulfur analogs, which are well known as potent monoamine oxidases (MAO) A/B inhibitors (Secci et al., 2012; Carradori et al., 2018; Oncü Can et al., 2018; Tripathi et al., 2018) with good antioxidative properties, there is no study of MAO A/B inhibition capacity of this class of selenium compounds to the best of our knowledge. Our recent study on pyridine-based (1,3-chalcogenazole-2-yl)hydrazones revealed that selenium-based compounds exhibited lower toxicity and superior antioxidant properties in comparison to their sulfur analogs (Filipović et al., 2017).

Modern treatment of complex multifactorial diseases, such as cancer and neurodegeneration, is transferred from development of single-targeting agents to simultaneous interactions with multiple targets via multi-targeting agents (MTAs) (Talevi, 2015). Both, neurodegeneration and cancer have their own molecular targets which need to be considered for design of novel MTAs. In the case of neurodegeneration, monoamine oxidases (MAO) A/B are suggested as one of the main targets for design of novel MTAs (Ramsay et al., 2016), while novel MTAs for the treatment of cancer are focused on targets like DNA and cancer-related proteins (Fu et al., 2017). However, since oxidative stress significantly contributes to the pathogenesis of cancer and neurodegeneration, novel effective MTAs should possess also good antioxidant properties (Lü et al., 2010; Carradori et al., 2018).

**Figure 1:**
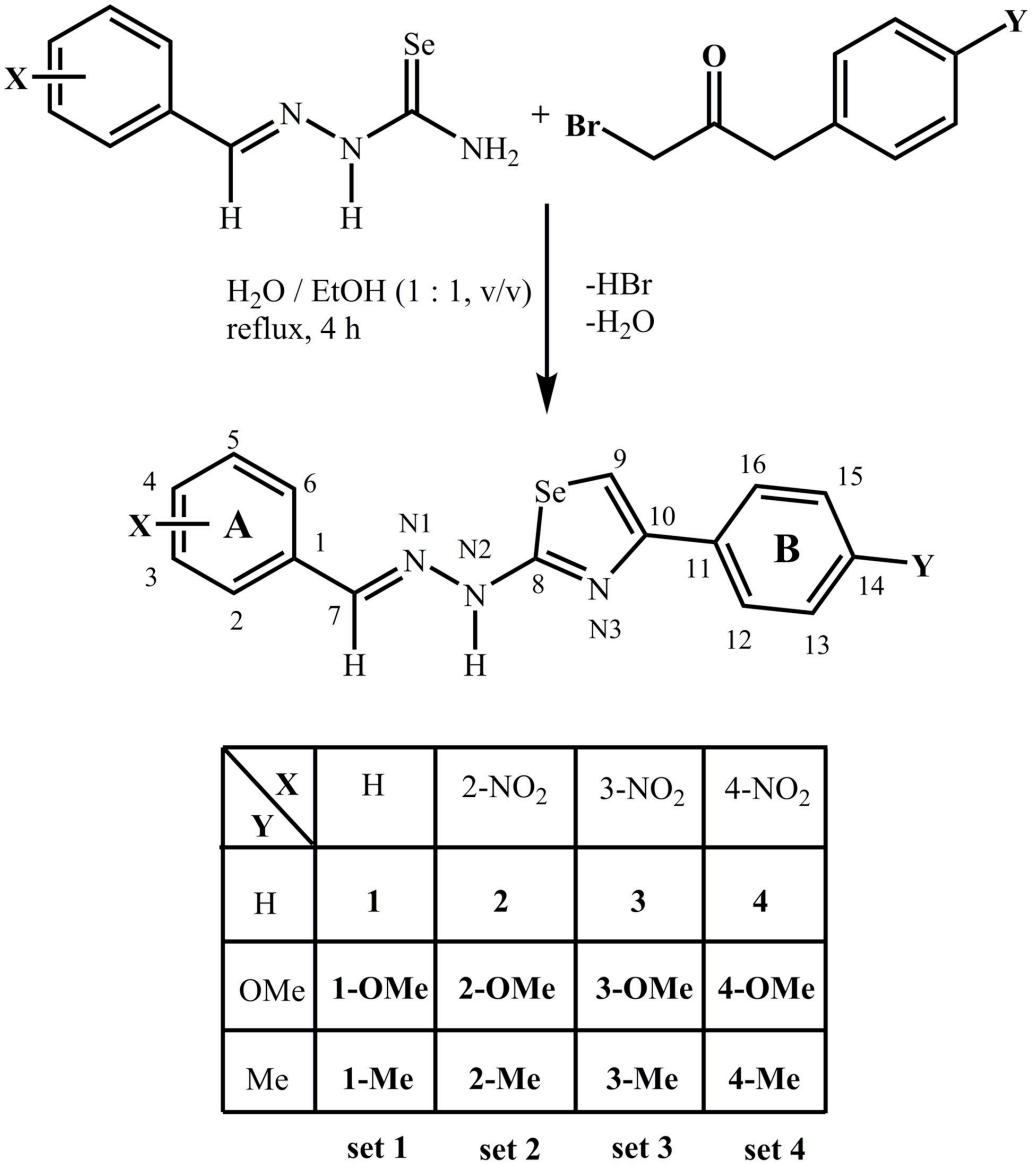
Synthesis of benzylidene-based (1,3-selenazol-2-yl) hydrazones studied in this work.

Since biological activity is influenced by the structural and molecular properties, particularly electronic properties, future prospects for design and development of new compounds with potential targeted biological activity can be based on the information obtained from experimental and theoretical results. In this work we designed a focused library of 12 structurally related benzylidene-based (1,3-selenazol-2-yl)hydrazones (Figure 1) and tested their antiproliferative, antioxidative and MAO A/B inhibition properties. In order to evaluate the multi-targeting properties of investigated compounds to both, Parkinson's disease and cancer, possible targets for the most active compounds were suggested by the similarity ensemble approach (SEA) (Keiser et al., 2007).

## Materials and methods

### Chemicals and drugs

Potassium selenocyanate (99%, Cat. No. 201980500), hydrazine monohydrate (100%, Cat. No. 196711000), 2-bromoacetophenone (98%, Cat. No. 152010250), and 2-bromo-4′-methylacetophenone (97%, 170390250) were obtained from Acros Organics. Benzaldehyde (≥99.5%, Cat. No. 418099), 2-nitrobenzaldehyde (98%, Cat. No. N10802), 3-nitrobenzaldehyde (99%, Cat. No. N10845) and 4-nitrobenzaldehyde (98%, 130176), diphenyl-1-picrylhydrazyl (DPPH, Cat. No. D9132), 2,2′-azobis(2-methylpropionamidine) dihydrochloride (AAPH; 97%, Cat. No. 440914), and fluorescein sodium salt (Cat. No. 46960-25G-F) were obtained from Sigma. 2-Bromo-4′-methoxyacetophenone (97%, Cat. No. CD00356EB) was obtained from Maybridge. Acetone selenosemicarbazone and the precursors, benzaldehyde selenosemicarbazone and 2-, 3-, and 4-nitrobenzaldehyde selenosemicarbazones, were synthesized according reported literature procedures (Huls and Renson, 1956). All solvents used in synthesis (reagent grade) were obtained from commercial suppliers and used without further purification.

### Instruments

Melting points were determined using an Electro thermal IA 9200 digital melting point apparatus and are uncorrected. Elemental analyses (C, H, N) were performed by the standard micromethods using the ELEMENTAR Vario EL III CHNS/O analyzer. Elemental analyses are within ±0.4%, confirming >95% purity. Infra-red (IR) spectra were recorded on a Thermo Scientific Nicolet 6700 FT-IR spectrometer by the Attenuated Total Reflection (ATR) technique in the region 4,000–400 cm^−1^. Abbreviations used for IR spectra: vs, very strong; s, strong; m, medium; w, weak. The NMR spectra (1D and 2D) were recorded on a Bruker Avance 500. Chemical shifts are given on δ scale relative to tetramethylsilane. Abbreviations used for NMR spectra: s, singlet; br. s, broad singlet; d, doublet; t, triplet; dd, double of doublets; m, multiplet; ovlp., overlapping. Atom numbering is given in Figure 1. BioTek'sPowerWave XS Tecan Infinite® M1000 PRO multimode reader was used for measurment of fluorescence intensity in MAO assay.

### General procedure for preparation of benzylidene-based (1,3-selenazol-2-yl)hydrazones

All compounds were prepared by the reaction of corresponding selenosemicarbazones and α-bromocarbonyl derivatives. Selenosemicarbazones (0.4 mmol) were suspended in 20 mL of water/EtOH (1:1, v/v) solvent mixture and 0.4 mmol of α-bromocarbonyl derivatives were added. The reaction mixtures were refluxed with stirring for 4 h. After completion of the reactions, monitored by TLC (ethyl acetate/hexane 1:1, v/v), the obtained precipitates were filtered off and washed with cold EtOH. The crude products were recrystallized from acetonitrile.

#### 2-(2-benzylidenehydrazinyl)-4-phenyl-1,3-selenazole (1)

Yield: 0.10 g (77%). Mp: 178–180°C. IR (ATR, ν_max_/cm^−1^): 3,298 (w), 3,139 (w), 3,058 (w), 2,959 (w), 2,852 (w), 1,602 (w), 1,559 (vs), 1,483 (m), 1,435 (m), 1,347 (w), 1,263 (s), 1,120 (m), 1,040 (w), 1,023 (w), 934 (w), 754 (m), 710 (m), 689 (m), 507 (w). ^1^H NMR (500.26 MHz, DMSO-*d*_6_) δ: 7.29 (t, 1H, H–C14, *J* = 7.2 Hz), 7.34–7.48 (m, 5H, H–C4, H–C3 = H–C5, H–C13 = H–C15), 7.66 (d, 2H, H–C2 = H–C6, *J* = 7.2 Hz), 7.69 (s, 1H, H–C9), 7.85 (d, 2H, H–C12 = H–C16, *J* = 7.2 Hz), 8.09 (s, 1H, H–C7), 12.27 (br. s, 1H, H–N2). ^13^C NMR (126.0 MHz, DMSO-*d*_6_) δ: 107.95 (C9), 126.45 (C12 = C16), 126.97 (C2 = C6), 128.01 (C14), 129.25 (C13 = C15), 129.53 (C3 = C5), 130.03 (C4), 135.04 (C1), 136.10 (C11), 142.88 (C7), 151.62 (C10), 171.93 (C8). Anal. Calcd. for C_16_H_13_N_3_Se (%): C, 58.90; H, 4.02; N, 12.88. Found: C, 58.62; H, 4.22; N, 12.67%.

#### 2-(2-benzylidenehydrazinyl)-4-(4-methoxyphenyl)-1,3-selenazole (1-OMe)

Yield: 0.13 g (91%). Mp: 180–182°C. IR (ATR, ν_max_/cm^−1^): 3,140 (m), 3,062 (m), 3,004 (m), 2,958 (m), 2,905 (m), 2,836 (m), 1,612 (m), 1,572 (vs), 1,490 (s), 1,436 (m), 1,355 (m), 1,320 (m), 1,300 (m), 1,265 (m), 1,246 (s), 1,175 (m), 1,124 (m), 1,027 (s), 937 (w), 832 (m), 755 (m), 694 (m), 610 (w), 528 (w). ^1^H NMR (500.26 MHz, DMSO-*d*_6_) δ: 3.78 (s, 3H, H–C17), 6.96 (d, 2H, H–C13 = H–C15, *J* = 8.7 Hz), 7.39 (dd, 1H, H–C4, *J* = 8.3 Hz, *J* = 6.2 Hz), 7.44 (t, 2H,H–C3 = H–C5, *J* = 7.4 Hz), 7.51 (s, 1H, H–C9), 7.67 (d, 2H, H–C2 = H–C6, *J* = 7.4 Hz), 7.78 (d, 2H, H–C12 = H–C16, *J* = 8.7 Hz), 8.09 (s, 1H, H–C7), 12.24 (br. s, 1H, H–N2). ^13^C NMR (126.0 MHz, DMSO-*d*_6_) δ: 55.68 (C17), 105.34 (C9), 114.50 (C12 = C16), 126.86 (C13 = C15), 127.65 (C2 = C6), 128.85 (C11), 129.42 (C3 = C5), 129.89 (C4), 135.00 (C1), 142.80 (C7), 150.78 (C10), 159.21 (C14), 171.70 (C8). Anal. Calcd. for C_17_H_15_N_3_OSe (%): C, 57.31; H, 4.24; N, 11.79. Found: C, 57.64; H, 4.35; N, 11.92%.

#### 2-(2-benzylidenehydrazinyl)-4-(p-tolyl)-1,3-selenazole (1-Me)

Yield: 0.07 g (51%). Mp: 174–176°C. IR (ATR, ν_max_/cm^−1^): 3,143 (m), 3,057 (m), 2,911 (m), 2,859 (m), 1,617 (m), 1,576 (vs), 1,561 (vs), 1,492 (m), 1,436 (m), 1,264 (m), 1,039 (m), 823 (m), 751 (m), 689 (m), 611 (w). ^1^H NMR (500.26 MHz, DMSO-*d*_6_) δ: 2.31 (s, 3H, H–C17), 7.21 (d, 2H, H–C13 = H–C15,*J* = 8.0 Hz), 7.39 (t, 1H, H–C4, *J* = 7.2 Hz), 7.44 (t, 2H, H–C3 = H–C5, *J* = 7.4 Hz), 7.59 (s, 1H, H–C9), 7.67 (d, 2H,H–C2 = H–C6, *J* = 7.4 Hz), 7.72 (d, 2H, H–C12 = H–C16, *J* = 8.0 Hz), 8.09 (s, 1H, H–C7), 12.26 (br. s, 1H, H–N2). ^13^C NMR (126.0 MHz, DMSO-*d*_6_) δ: 21.37 (C17), 106.71 (C9), 126.29 (C12 = C16), 126.87 (C2 = C6), 129.43 (C13 = C15), 129.72 (C3 = C5), 129.91 (C4), 133.32 (C11), 134.98 (C1), 137.17 (C14), 142.80 (C7), 151.24 (C10), 171.73 (C8). Anal. Calcd. for C_17_H_15_N_3_Se (%): C, 60.00; H, 4.44; N, 12.35. Found: C, 60.23; H, 4.67; N, 12.59%.

#### 2-(2-(2-nitrobenzylidene)hydrazinyl)-4-phenyl-1,3-selenazole (2)

Yield: 0.083 g (56%). Mp: 183–184°C.IR (ATR, ν_max_/cm^−1^): 3,164 (w), 3,115 (w), 3,047 (w), 2,963 (w), 2,851 (m), 2,792 (m), 1,580 (vs), 1,518 (vs), 1,481 (m), 1,438 (m), 1,341 (s), 1,297 (m), 1,266 (s), 1,128 (m), 1,043 (m), 1,023 (w), 918 (w), 904 (w), 847 (w), 780 (w), 747 (w), 706 (m), 660 (m).^1^H NMR (500.26 MHz, DMSO-*d*_6_) δ: 7.30 (t, 1H, H–C14, *J* = 7.3 Hz), 7.39 (t, 2H, H–C13 = H–C15, *J* = 7.7 Hz), 7.61 (m, 1H, H–C5), 7.74 (s, 1H, H–C9), 7.78 (t. 1H, H–C4, *J* = 7.7 Hz), 7.84 (d, 2H, H–C12 = H–C16,*J* = 7.4 Hz), 8.01 (d, 1H, H–C3, *J* = 0.7 Hz), 8.03 (s. 1H, H–C6), 8.45 (s, 1H, H–C7).^13^C NMR (126.0 MHz, DMSO-*d*_6_) δ: 108.77 (C9), 125.35 (C3), 126.45 (C12 = C16), 128.13 (C14), 128.29 (C6), 129.10 (C1), 129.29 (C13 = C15), 130.49 (C5), 134.16 (C4), 135.86 (C11), 137.93 (C7), 148.13 (C2), 151.39 (C10), 171.81 (C8). Anal. Calcd. for C_16_H_12_N_4_O_2_Se (%): C, 51.76; H, 3.26; N, 15.09. Found: C, 52.32; H, 2.60; N, 15.14%.

#### 4-(4-methoxyphenyl)-2-(2-(2-nitrobenzylidene)hydrazinyl)-1,3-selenazole (2-OMe)

Yield: 0.104 g (66%), Mp: 165–166°C. IR (ATR, ν_max_/cm^−1^): 3,108 (w), 3,030 (w), 2,954 (w), 2,901 (w), 2,830 (m), 2,654 (m), 2,324 (w), 1,603 (w), 1,569 (s), 1,516 (s), 1,493 (m), 1,441 (m), 1,364 (w), 1,337 (s), 1,307 (m), 1,242 (s), 1,169 (m), 1,126 (m), 1,040 (m), 918 (w), 837 (m), 785 (w), 745 (w), 715 (w). ^1^H NMR (500.26 MHz, DMSO-*d*_6_) δ: 3.77 (s, 3H, H–C17), 6.95 (d, 2H, H–C13 = H–C15, *J* = 8.8 Hz), 7.54 (s, 1H, H–C9), 7.60 (m, 1H, H–C5), 7.74–7.80 (m, 3H, H–C4 and H–C12 = H–C16), 8.01 (d, 1H, H–C3, *J* = 1.0 Hz), 8.02 (d, 1H, H–C6, *J* = 1.0 Hz), 8.45 (s, 1H, H–C7). ^13^C NMR (126.0 MHz, DMSO-*d*_6_) δ: 55.79 (C17), 106.30 (C9), 114.63 (C13 = C15), 125.34 (C3), 127.77 (C12 = C16), 128.27 (C6), 128.67 (C11), 129.14 (C1), 130.44 (C5), 134.15 (C4), 137.95 (C7), 148.12 (C2), 150.82 (C10), 159.38 (C14), 171.73 (C8). Anal. Calcd. for C_17_H_14_N_4_O_3_Se (%): C, 50.88; H, 3.52; N, 13.96. Found: C, 51.71; H, 1.95; N, 13.98%.

#### 2-(2-(2-nitrobenzylidene)hydrazinyl)-4-(p-tolyl)-1,3-selenazole (2-Me)

Yield: 0.108 g (70%). Mp: 170–171°C. IR (ATR, ν_max_/cm^−1^): 3,305 (w), 3,156 (w), 3,116 (w), 3,074 (w), 2,971 (w), 2,917 (w), 2,858 (w), 1,569 (vs), 1,521 (vs), 1,439 (m), 1,337 (s), 1,294 (m), 1,262 (m), 1,179 (m), 1,125 (m), 1,038 (m), 922 (w), 846 (w), 828 (w), 748 (w), 730 (w), 709 (w). ^1^H NMR (500.26 MHz, DMSO-*d*_6_) δ: 2.30 (s, 3H, H–C17), 7.19 (d, 2H,H–C13 = H–C15, *J* = 8.1 Hz), 7.58–7.63 (m, 1H, H–C5), 7.64 (s, 1H, H–C9), 7.72 (d, 2H, H–C12 = H–C16, *J* = 8.1 Hz), 7.75–7.80 (m, 1H, H–C4), 8.01 (d, 1H, H–C3, *J* = 1.0 Hz), 8.02 (d, 1H, H–C6, *J* = 1.0 Hz), 8.45 (s, 1H, H–C7). ^13^C NMR (126.0 MHz, DMSO-*d*_6_) δ: 20.84 (C17), 107.03 (C9), 124.73 (C3), 125.77 (C12 = C16), 127.66 (C6), 128.50 (C1), 129.23 (C13 = C15), 129.84 (C5), 132.55 (C11), 133.53 (C4), 136.80 (C14), 137.30 (C7), 147.50 (C2), 150.71 (C10), 171.21 (C8). Anal. Calcd. for C_17_H_14_N_4_O_2_Se (%): C, 53.00; H, 3.66; N, 14.54. Found: C, 52.59; H, 3.18; N, 14.36%.

#### 2-(2-(3-nitrobenzylidene)hydrazinyl)-4-phenyl-1,3-selenazole (3)

Yield: 0.108 g (73%). Mp: 216–218°C. IR (ATR, ν_max_/cm^−1^): 3,163 (w), 3,062 (w), 2,962 (w), 2,862 (m), 2,804 (w), 1,608 (w), 1,580 (s), 1,526 (vs), 1,482 (m), 1,453 (m), 1,345 (s), 1,268 (m), 1,131 (m), 1,093 (w), 928 (w), 734 (m), 704 (m), 673 (w). ^1^H NMR (500.26 MHz, DMSO-*d*_6_) δ: 7.30 (t, 1H, H–C14, *J* = 7.3 Hz), 7.39 (t, 2H, H–C13 = H–C15, *J* = 7.6 Hz), 7.71 (t, 2H, ovlp. H–C5 and H–C9, *J* = 7.9 Hz), 7.84 (d, 2H, H–C12 = H–C16, *J* = 7.4 Hz), 8.08 (d, 1H, H–C6, *J* = 7.8 Hz), 8.19 (m, 2H, ovlp. H–C4 and H–C7), 8.45 (s, 1H, H–C2), 12.54 (s, 1H, H–N2).^13^C NMR (126.0 MHz, DMSO-*d*_6_) δ: 107.71 (C9), 120.40 (C2), 123.49 (C4), 125.83 (C12 = C16), 127.52 (C14), 128.68 (C13 = C15), 130.49 (C5), 132.31 (C6), 135.23 (C11), 136.33 (C3), 140.02 (C7), 148.34 (C1), 150.86 (C10), 171.30 (C8). Anal. Calcd. for C_16_H_12_N_4_O_2_Se (%): C, 51.76; H, 3.26; N, 15.09. Found: C, 51.54; H, 3.00; N, 15.11%.

#### 4-(4-methoxyphenyl)-2-(2-(3-nitrobenzylidene)hydrazinyl)-1,3-selenazole (3-OMe)

Yield: 0.112 g (70%). Mp: 201–203°C. IR (ATR, ν_max_/cm^−1^): 3,161 (w), 3,049 (w), 2,958 (m), 2,829 (m), 2,371 (w), 1,581 (s), 1,523 (vs), 1,489 (m), 1,452 (m), 1,330 (s), 1,320 m), 1,300 (m), 1,272 (m), 1,245 (s), 1,174 (m), 1,131 (s), 1,032 (m), 930 (w), 834 (m), 752 (w), 731 (w), 675 (w).^1^H NMR (500.26 MHz, DMSO-*d*_6_) δ: 3.77 (s, 3H, H–C17), 6.95 (m, 2H, H–C13 = H–C15), 7.52 (s, 1H, H–C9), 7.71 (t, 1H, H–C5, *J* = 8.0 Hz), 7.76 (m, 2H, H–C12 = H–C16), 8.08 (m, 1H, H–C6), 8.19 (m, 2H, ovlp. H–C4 and H–C7), 8.45 (m, 1H, H–C2), 12.44 (s, 1H, H–N2).^13^C NMR (126.0 MHz, DMSO-*d*_6_) δ: 55.16 (C17), 105.05 (C9), 114.00 (C13 = C15), 120.37 (C2), 123.44 (C4), 127.13 (C12 = C16), 127.98 (C11), 130.47 (C5), 132.28 (C6), 136.38 (C3), 140.00 (C7), 148.33 (C1), 150.60 (C10), 158.75 (C14), 171.21 (C8).Anal. Calcd. for C_17_H_14_N_4_O_3_Se (%): C, 50.88; H, 3.52; N, 13.96. Found: C, 51.55; H, 3.17; N, 14.12%.

#### 2-(2-(3-nitrobenzylidene)hydrazinyl)-4-(p-tolyl)-1,3-selenazole (3-Me)

Yield: 0.106 g (69%), Mp: 203–206°C.IR (ATR, ν_max_/cm^−1^): 3,154 (w), 3,111 (w), 3,046 (w), 2,912 (m), 2,856 (m), 2,363 (w), 1,602 (w), 1,575 (s), 1,523 (vs), 1,486 (m), 1,448 (m), 1,342 (s), 1,266 (m), 1,177 (w), 1,130 (m), 1,039 (m), 925 (w), 830 (w), 735 (w), 674 (w). ^1^H NMR (500.26 MHz, DMSO-*d*_6_) δ: 2.30 (s, 3H, H–C17), 7.19 (d, 2H, H–C13 = H–C15, *J* = 8.0 Hz), 7.62 (s, 1H, H–C9), 7.71 (m, 3H, ovlp. H–C5 and H–C12 = H–C16), 8.08 (d, 1H, H–C6, *J* = 7.8 Hz), 8.18 (dd, 1H, H–C4, *J* = 2.3 Hz, *J* = 0.8 Hz), 8.20 (s,1H, H–C7), 8.44 (m, 1H, H–C2), 12.46 (s, 1H, H–N2).^13^C NMR (126.0 MHz, DMSO-*d*_6_) δ: 20.82 (C17), 106.54 (C9), 120.37 (C2), 123.44 (C4), 125.74 (C12 = C16), 129.21 (C13 = C15), 130.45 (C5), 132.28 (C6), 132.50 (C11), 136.35 (C3), 136.78 (C14), 140.06 (C7), 148.32 (C1), 150.60 (C10), 171.22 (C8). Anal. Calcd. for C_17_H_14_N_4_O_2_Se (%): C, 53.00; H, 3.66; N, 14.54. Found: C, 53.23; H, 3.36; N, 14.59%.

#### 2-(2-(4-nitrobenzylidene)hydrazinyl)-4-phenyl-1,3-selenazole (4)

Yield: 0.102 g (69%). Mp: 228–230°C. IR (ATR, ν_max_/cm^−1^): 3290 (m), 3,110 (w), 1,584 (w), 1,554 (m), 1,511 (s), 1,440 (w), 1,321 (m), 1,282 (m), 1,146 (w), 1,040 (w), 920 (w), 847 (s), 774 (w), 711 (m), 689 (w).^1^H NMR (500.26 MHz, DMSO-*d*_6_) δ: 7.30 (t, 1H, H–C14, *J* = 7.3 Hz), 7.39 (t, 2H, H–C13 = H–C15, *J* = 7.6 Hz), 7.75 (s, 1H, H–C9), 7.84 (d, 2H, H–C12 = H–C16, *J* = 7.3 Hz), 7.88 (d, 2H, H–C2 = H–C6, *J* = 8.9 Hz), 8.16 (s, 1H, H–C7), 8.26 (d, 2H, H–C3 = H–C5, *J* = 8.9 Hz), 12.65 (s, 1H, H–N2). ^13^C NMR (126.0 MHz, DMSO-*d*_6_) δ: 107.96 (C9), 124.14 (C3 = C5), 125.76 (C12 = C16), 126.99 (C2 = C6), 127.47 (C14), 128.61 (C13 = C15), 135.16 (C11), 139.81 (C7), 140.75 (C1), 147.14 (C4), 150.88 (C10), 171.14 (C8). Anal. Calcd. for C_16_H_12_N_4_O_2_Se (%): C, 51.76; H, 3.26; N, 15.09. Found: C, 51.76; H, 3.05; N, 15.10%.

#### 4-(4-methoxyphenyl)-2-(2-(4-nitrobenzylidene)hydrazinyl)-1,3-selenazole (4-OMe)

Single crystals suitable for X-ray diffraction analysis were obtained from acetonitrile solution after 2 days. Yield: 0.119 g (74%). Mp: 208–211°C. IR (ATR, ν_max_/cm^−1^): 2,661 (w), 1,587 (m), 1,565 (m), 1,532 (w), 1,512 (s), 1,488 (m), 1,454 (m), 1,376 (w), 1,341 (s), 1,248 (m), 1,172 (m), 1,142 (m), 1,106 (m), 1,044 (m), 1,022 (m), 925 (m), 908 (w), 876 (w), 840 (m), 747 (w), 684 (w). ^1^H NMR (500.26 MHz, DMSO-*d*_6_) δ: 3.78 (s, 3H, H–C17), 6.95 (d, 2H,H–C13 = H–C15, *J* = 8.8 Hz), 7.56 (s, 1H, H–C9), 7.76 (d, 2H, H–C12 = H–C16, *J* = 8.7 Hz), 7.78 (d, 2H, H–C2 = H–C6, *J* = 8.8 Hz), 8.16 (s, 1H, H–C7), 8.26 (d, 2H, H–C3 = H–C5, *J* = 8.8 Hz) 12.58 (s, 1H, H–N2).^13^C NMR (126.0 MHz, DMSO-*d*_6_) δ: 55.15 (C17), 105.49 (C9), 113.99 (C13 = C15), 124.18 (C3 = C5), 127.02 (C2 = C6), 127.12 (C12 = C16), 127.94 (C11), 139.84 (C7), 140.87 (C1), 147.16 (C4), 150.88 (C10), 158.76 (C14), 171.18 (C8). Anal. Calcd. for C_17_H_14_N_4_O_3_Se (%): C, 50.88; H, 3.52; N, 13.96. Found: C, 50.80; H, 3.30; N, 14.02%.

#### 2-(2-(4-nitrobenzylidene)hydrazinyl)-4-(p-tolyl)-1,3-selenazole (4-Me)

Single crystals suitable for X-ray diffraction analysis were obtained from acetonitrile solution after 3 days. Yield: 0.111 g (72%), Mp: 228–230°C. IR (ATR, ν_max_/cm^−1^): 3,110 (w), 2,922 (w), 2,668 (m), 1,608 (m), 1,587 (s), 1,565 (s), 1,513 (vs), 1,456 (s), 1,409 (w), 1,374 (w), 1,341 (vs), 1,320 (s), 1,285 (s), 1,177 (w), 1,144 (m), 1,107 (w), 1,042 (m), 924 (w), 907 (w), 849 (w), 828 (m), 746 (w). ^1^H NMR (500.26 MHz, DMSO-*d*_6_) δ: 2.30 (s, 3H, H–C17), 7.19 (d, 2H, H–C13 = H–C15, *J* = 8.0 Hz), 7.65 (s, 1H, H–C9), 7.72 (d, 2H, H–C12 = H–C16, *J* = 8.1 Hz), 7.88 (d, 2H, H–C2 = H–C6, *J* = 8.9 Hz), 8.16 (s, 1H, H–C7), 8,25 (d, 2H,H–C3 = H–C5, *J* = 8.9 Hz), 12.58 (s, 1H, H–N2). ^13^C NMR (126.0 MHz, DMSO-*d*_6_) δ: 20.83 (C17), 107.10 (C9), 124.19 (C3 = C5), 125.76 (C12 = C16), 127.03 (C2 = C6), 129.23 (C13 = C15), 132.48 (C11), 136.83 (C14), 139.80 (C7), 140.86 (C1), 147.17 (C4), 150.56 (C10), 171.19 (C8). Anal. Calcd. for C_17_H_14_N_4_O_2_Se (%): C, 53.00; H, 3.66; N, 14.54. Found: C, 53.37; H, 3.22; N, 14.70%.

### X-ray crystallography

Data collection for single crystals of **4-Me** and **4-OMe** was performed at room temperature on an Oxford Diffraction Xcalibur Nova R diffractometer with a microfocusing Cu tube (λ = 1.54179 Å). Data reduction and cell refinement were carried out using the CRYSALIS PRO software (Oxford Diffraction Ltd.,, 2009). Structures were solved by direct methods with SIR2014 (Burla et al., 2015) and refined by a full matrix least-squares refinement based on *F*^2^, with SHELXL (Sheldrick, 1997). Molecular illustrations were prepared with ORTEP-3 (Farrugia, 1997) and MERCURY (Macrae et al., 2008) included into the WinGX package (Farrugia, 1999). Calculations of molecular geometries and crystal packing parameters were performed with PLATON (Spek, 2009). Hydrogen atoms of the phenyl rings and of the methyl moieties, as well as the H12 for the compound **4-Me** were included in their geometrically calculated positions and refined according to the riding model, while the others were located in the Fourier map and refined freely. CCDC 1494791 and 1494792 contain the supplementary crystallographic data. These data can be obtained free of charge *via* http://www.ccdc.cam.ac.uk/conts/retrieving.html, or from the Cambridge Crystallographic Data Centre, 12 Union Road, Cambridge CB2 1EZ, UK; fax: (+44) 1223-336-033; or e-mail: deposit@ccdc.cam.ac.uk.

### Cyclic voltammetry

The three-electrode system was used, glassy carbon (3 mm in diameter, CHI 104), non-aqueous Ag/Ag^+^ reference with porous Teflon tip (CHI 112) and Pt-wire as counter electrode. The reference electrode was filled with 10 mM AgNO_3_ solution in 0.1 M tetrabutylammonium perchlorate (TBAP) in DMSO. The potential was scanned between −2.0 and +1.0 V at scan rate of 100 mV s^−1^ in anodic positive mode. All measurements were performed at room temperature. Working solutions were deaerated for 15 min with nitrogen. The voltammograms were recorded in 0.1 M TBAP/DMSO. Concentration of the compounds was 1 mM.

### MAO A/B inhibition

MAO A and B inhibition capacities were investigated using a discontinuous fluorimetric assay (Meiring et al., 2013) as described previously by using human recombinant membrane-bound MAO purchased from Sigma-Aldrich (Affini et al., 2018). Briefly, remained enzyme activity with inhibitor, either 1 μM (one-point) or concentrations ranging from 0.0001 to 10 μM, was assessed in the presence of kynuramine [2 × K_M_, K_M_ = 20 μM (MAO A), K_M_ = 30 μM (MAO B)] in potassium phosphate buffer (pH 7.4). Reactions were started by addition of enzyme and stopped by manual addition of sodium hydroxide (2 N) after 20 min. Enzyme activity was determined by detection of 4-hydroxyquinoline (λ_Ex_ = 320 nm, λ_Em_ = 405 nm). Data were analyzed using GraphPad PRISM 6. For one-point measurements data were calculated as percentage of control and expressed as mean ± standard deviation (%). IC_50_ values are given as mean within the 95% confidence interval (CI). Data were obtained from two (one-point measurements) or at least three (IC_50_ values) independent experiments, each performed in duplicates.

### Assessment of antioxidant capacity

#### DPPH scavenging antioxidant assay

The experiments were performed according to the literature protocol (Prior et al., 2005). All tested compounds were initially dissolved in DMSO. The initial concentration of DPPH in methanol was 6.58 × 10^−5^ M. A volume of 140 μL of DPPH solution was placed into a 96-well microplate, and then 10 μL solution of the tested compounds was added. Pure DMSO (10 μL) was used as the control. The absorbance at 517 nm was measured after 30 min period of incubation in the dark at 25°C. The Equation (1) was used for calculation of the scavenging activity:

(1) $$\begin{array}{r} Scavenging\;activity\;\left(\%\right)=\frac{A_{control}-\;A_{sample}}{A_{control}}\times 100 \end{array}$$

where *A*_*sample*_ and *A*_*control*_ refer to the absorbances at 517 nm of the sample and control, respectively. IC_50_ values were calculated from the graph of scavenging activity against the concentrations of the samples. IC_50_ represents the total antioxidant concentration of the sample which decreases the amount of the initial DPPH radical by 50%. Ascorbic acid (vitamin C) was used as positive control (concentration range 10–500 μM).

#### Total reducing power (TRP) (modified potassium ferricyanide reduction method)

The mixture containing 0.5 mL of phosphate buffer (0.2 M, pH = 6.6), 0.5 mL of K_3_**[**Fe(CN)_6_] (1%; w/v) and 0.5 mL of the samples (100–1,500 μM) was incubated at 50°C for 20 min. A volume of 0.5 mL of trichloroacetic acid (TCA, 10%; w/v), 0.5 mL of Milli-Q water and 0.5 mL of FeCl_3_ (0.1%; w/v) was added, followed by intensive vortexing. The absorbance of the resulting mixture was measured after 60 min at 700 nm using phosphate buffer as blank (Oyaizu, 1986).

#### Total antioxidant capacity (TAOC) (modified phosphomolybdenum method)

Volume of 0.4 mL of sample solution (50–1,000 μM) was mixed with 1.6 mL of reagent solution [0.6 M H_2_SO_4_, 28 mM Na_3_PO_4_, and 4 mM (NH_4_)_2_MoO_4_] and resulting mixtures were incubated at 95°C for 90 min. The cooled reaction mixtures were then centrifuged for 10 min (3,000 rpm). The absorbance of the supernatant solution was measured, 1 h after centrifugation, at 695 nm against reagent solution as blank. An increased absorbance in reading in both assays indicated increased antioxidant power, expressed as EC_50_ values (the sample concentration giving absorbance of 0.500 from the graph of absorbance at 700 nm or 695 nm against compound concentration) (Prieto et al., 1999).

#### Oxygen radical absorbance capacity (ORAC) assay

A modification of original protocol was used (Ou et al., 2001). Stock solutions of fluorescein substrate (5 μM) and free radical generator AAPH (0.5 M) were prepared in 75 mM potassium phosphate buffer (pH = 7.4). Volume of 100 μL of sample solutions or Trolox in DMSO (20 μM) were mixed with 1,485 μL of buffer and 15 μL of fluorescein solution. The 30 min reaction at 37°C was initiated by adding 250 μL of AAPH solution. Fluorescence conditions were as follows: excitation and emission wavelengths 485 and 511 nm, respectively, slits 2 nm. The relative sample ORAC value was expressed as Trolox equivalents (TE).

### Antiproliferative activity

The *in vitro* antiproliferative activity of investigated compounds was evaluated against six human solid tumor cell lines: A549 (non-small cell lung), HBL-100, (breast), HeLa (cervix), SW1573 (non-small cell lung), as drug sensitive lines, T-47D (breast) and WiDr (colon) as drug resistant lines. These cell lines were a kind gift from Prof. G. J. Peters (VU Medical Center, Amsterdam, The Netherlands). For selectivity studies, the human fibroblasts BJ-hTert cell line was used, which was obtained from Dr. R. Freire (HUC, Tenerife, Canary Islands). Cells were kept in culture medium under standard conditions: RPMI 1640 medium, fetal bovine serum (5%), glutamine (2 mM), penicillin G (100 units/mL) and streptomycin (0.1 mg/mL). Antiproliferative tests were conducted as described earlier (Skehan et al., 1990; Miranda et al., 2006). Tested compounds were dissolved in DMSO at an initial concentration of 40 mM. DMSO was used as negative control (0.25% v/v). Antiproliferative activity of the compounds was expressed as GI_50_, which is the concentration of the compound that inhibits 50% of the culture growth.

### *In-silico* studies

The geometries of neutral *E*-isomeric form for all structures were optimized at the density functional theory (DFT) level in the gas phase. Becke-3-Lee-Yang-Par functional (B3LYP) (Lee et al., 1988; Becke, 1993) and the double split valence 6-31G(d,p) basis set were used in the calculations (Hariharan and Pople, 1973; Francl, 1982; Rassolov et al., 1998, 2001). Optimized geometries of the investigated molecules in the gas phase are shown in Supplementary Figure S1. The gas phase calculated molecular structures were re-optimized in DMSO using the Polarisable Continuum Model (Scalmani and Frisch, 2010) with DFT/B3LYP/6-31G(d,p) method. All quantum chemical calculations were performed with Gaussian09 program package (Frisch et al., 2016).

Physicochemical properties, lipophilicity, water solubility, pharmacokinetics, druglikeness and medicinal chemistry parameters were determined using the free SwissADME tools available at website of the Swiss Institute of Bioinformatics (http://www.swissadme.ch/) (Daina et al., 2017). The structures were constructed and converted into SMILES format.

Possible suggestions for targets for compounds were found using SEA (Keiser et al., 2007), which can relate proteins by a similarity ensemble approach (initials, SEA) based on the chemical similarities of ligands. Crystal structures were obtained from the Protein Data Bank (Berman et al., 2000). The proteins corresponded to KCNN1 small conductance calcium-activated potassium channel protein 1 (5wbx, ligand HET-ID AJY; (3*Z*)-6-bromo-3-(hydroxyimino)-5-methyl-1,3-dihydro-2H-indol-2-one) and MAO-B (4crt, ligand HET-ID ASS234; (*E*)-*N*-methyl-*N*-[[1-methyl-5-[3-[1-(phenylmethyl)piperidin-4-yl]propoxy]indol-2-yl]methyl]prop-1-en-1-amine), implicated in neurodegenerative diseases; as well as eukaryotic initiation factor 4E (1ipb, ligand HET-ID GTA; P1-7-methylguanosine-P3-adenosine-5′,5′-triphosphate) and 5′-nucleotidase (4h2b, ligand HET-ID 0XE; 5,6-dihydroxy-4-oxo-2-phenyl-4H-chromen-7-yl beta-D-glucopyranosiduronic acid; Baicalin), implicated in cancer. All protein structures were determined at high resolution. Hydrogen atoms were added with Maestro software (Maestro, 2017). Docking was then performed by AutodockVina (Trott and Olson, 2010) using a box size of 25 Å in each dimension; nine modes; energy range of 1 kcal/mol; 1 cpu per run; exhaustiveness = 16; and 100 runs per ligand and per protein. In each case, the co-crystallized ligand was taken as a positive control, and the binding score recorded for it was used as threshold to determine binders.

## Results and discussion

### Synthesis and characterization

Twelve benzylidene-based (1,3-selenazol-2-yl)hydrazones were prepared *via* Hantzsch type condensation of corresponding selenosemicarbazones with a series of 4-substituted α-bromoacetophenones (Figure 1). Compounds **4-OMe** and **4-Me** crystallized as single crystals suitable for X-ray structural analysis, which indicated *E-*configuration of the imine bond (*vide infra*).

Synthesis of the compounds **1** and **1-Me** was previously published, but without spectral characterization (Bulka et al., 1961). Literature data for melting points of **1** and **1-Me** significantly differ from our data (Bulka et al., 1961). Composition of the compounds was confirmed by elemental analysis, while NMR and IR spectroscopy were used for structure elucidation. 1D and 2D NMR spectra are given in Supplementary Figures S2–S41. The influence of substituents on both phenyl rings, A and B, on NMR chemical shifts of corresponding hydrogen and carbon atoms was observed. As expected, in the ^1^H NMR spectra of all compounds the signal of H–N2 is the most downfielded. Substitution of the phenyl rings had negligible influence on chemical shift of a proton from 1,3-selenazole ring, thus the signal of the proton H–C9 in the ^1^H NMR spectra of all compounds appeared in the narrow range (7.51–7.71 ppm). Introduction of NO_2_ group on the phenyl ring A, which has negative inductive and negative resonance effect, caused downfield shift of signals of all protons in the ring in comparison to signals of corresponding protons in the ^1^H NMR spectra of compounds from set 1. Also, chemical shift of H–C7 protons was affected by this substitution, where for all compounds from set 2, with NO_2_ group in *ortho*-position, significant shift to lower field was observed. Introduction of methyl group on the phenyl ring B, which is electron donating group by induction, caused shielding effect of all protons from the ring B, where signals of protons H–C13 and H–C15 were the most affected in the ^1^H NMR spectra of all methyl derivatives. The electronic effects of methoxy group, which is a withdrawer by induction and an electron donor by resonance, is determined by its position. Since it participates in delocalization of π electrons from the phenyl ring B, it functions as a strong electron donor. This is again mostly reflected on chemical shifts of H–C13 and H–C15 protons in the ^1^H NMR spectra of all methoxy derivatives, where these protons are shielded and thus their signals are upfielded. Electronic effects of substituents have the similar impact on chemical shifts of corresponding carbon atoms in ^13^C NMR spectra.

### Analysis of crystal structures

**Figure 2:**
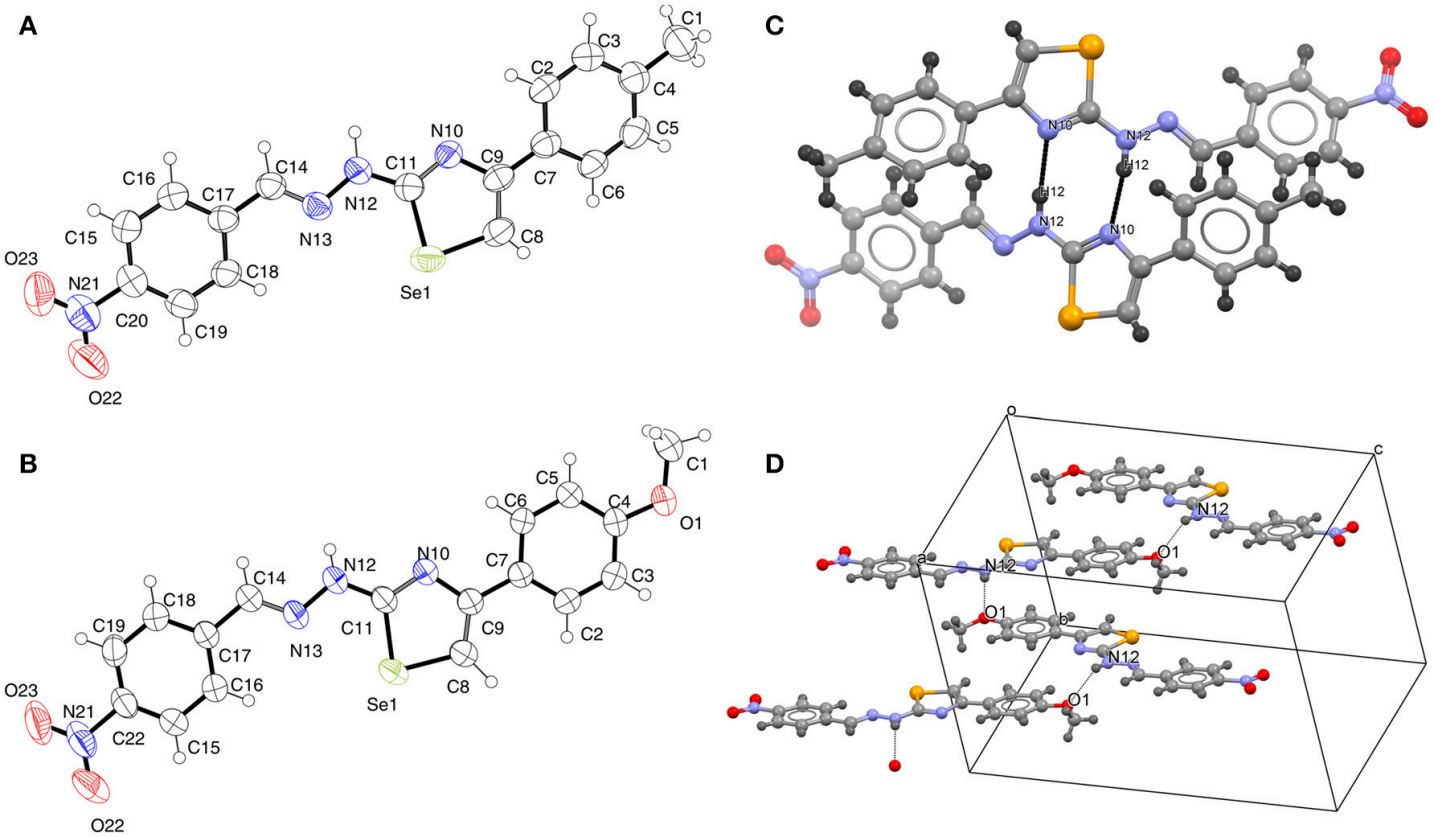
ORTEP drawings of the molecular structures of **4-Me (A)** and **4-OMe (B)** with non-H atoms labeling. Displacement ellipsoids are shown at the 50% probability level and H atoms are drawn as spheres of arbitrary radii. Crystal packing diagrams of **4-Me (C)** and **4-OMe (D)**.

**Figure 3:**
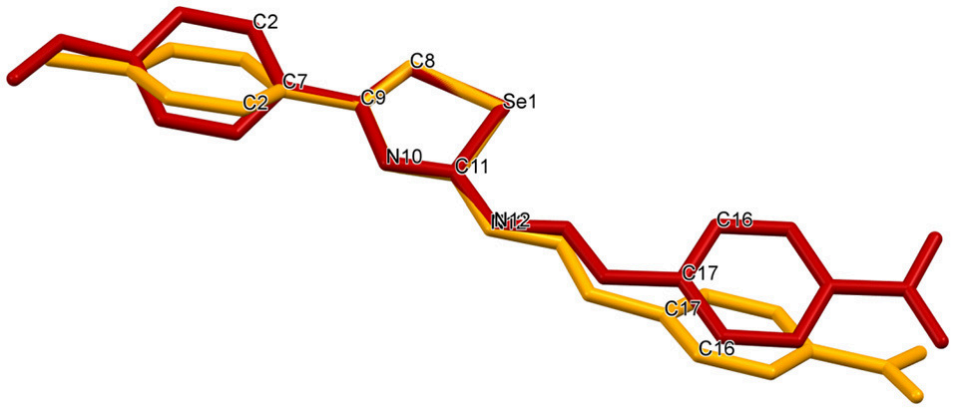
Structural superposition of 4-Me (yellow) and 4-OMe (red).

Relevant crystallographic data for **4-OMe** and **4-Me** are summarized in Supplementary Table S1. Molecular structures of **4-Me** and **4-OMe** with the atom numberings and crystal packing motifs are depicted in Figure 2, while selected bond lengths and bond angles are presented in Table 1. The geometries of the selenazole rings in both structures reveal no unusual parameters when compared with the set of related structures from the current version of CSD (Groom et al., 2016). Analysis of the interplanar angles defined by the least square plane of the selenazole ring and the least square planes of both phenyl rings reveals a certain level of planarity in the structure of **4-OMe** unlike in **4-Me** (Supplementary Table S2). Visually this result is depicted in Figure 3, which displays an overlay of molecular structures of **4-Me** and **4-OMe**. The torsion angle Se1–C11–N12–N13 [−7.3(4)° in **4-Me** and 1.3(3)° in **4-OMe**] reveals the *cis*-orientation of the N13 with respect to the selenium (and, consequently, *trans*-orientations with respect to the N10) in both structures, which are therefore conformationally prone to act as *N*,*Se* bidentate ligands in possible metal coordination. Basic crystallographic packing motif in the structure of **4-Me** is a distinct paddle wheel-like centrosymmetric molecular dimer formed by the hydrogen bond N12– 12···N10^*i*^ [*i* = 1–*x, y*, 1/2–*z*; *d* (N12–H): 0.86 Å, *d* (H···N10): 2.01 Å, *d* (N12···N10): 2.857(5) Å, angle: 168°] of the graph set notation R 2,2 (8) (Figure 2C). The paddlewheel axis running through Se1 and C11 of both involved molecules is roughly parallel with the crystallographic *a-*axis. In the structure of (roughly planar) **4-OMe** crystal packing is guided by a single hydrogen bond N12–H12···O1^*ii*^ [*ii* = 1/2+*x, y*, 1/2–*z*; *d* (N12–H): 0.81(4) Å, *d* (H···O1): 2.48 (3) Å, *d* (N12···O1): 3.202 (3) Å, angle: 150(3)°] through which the *zig-zag* oriented molecules are connected into the “endless” chains parallel to the crystallographic *ac* plane (Figure 2D).

**Table 1:** Selected experimentally obtained (XRD) and calculated (DFT) bond lengths (Å) and angles (°) for **4-Me** and **4-OMe**.

	**4-Me (XRD)**	**4-Me (DFT)**	**4-OMe (XRD)**	**4-OMe (DFT)**
C7–C9	1.475(5)	1.476	1.475(3)	1.476
C8–C9	1.353(5)	1.368	1.351(3)	1.369
C8–Se1	1.871(5)	1.874	1.873(2)	1.876
C9–N10	1.388(4)	1.391	1.391(2)	1.391
C11–N10	1.300(5)	1.291	1.290(2)	1.292
C11–N12	1.348(5)	1.375	1.361(3)	1.375
C11–Se1	1.872(4)	1.887	1.8808(19)	1.886
C14–N13	1.277(4)	1.290	1.271(3)	1.290
C14–C17	1.451(4)	1.459	1.463(3)	1.459
N12–N13	1.358(4)	1.338	1.354(2)	1.337
N21–O23	1.212(5)	1.232	1.214(3)	1.232
N21–O22	1.218(6)	1.232	1.222(3)	1.232
C9–C8–Se1	111.6(3)	111.16	111.86(16)	111.60
C8–C9–N10	116.0(3)	116.20	116.55(18)	116.20
C8–C9–C7	126.9(3)	125.90	125.85(19)	126.00
N10–C9–C7	117.1(3)	117.90	117.59(17)	117.80
N10–C11–N12	121.3(3)	122.10	124.32(18)	122.00
N10–C11–Se1	115.2(3)	116.30	116.57(15)	116.30
N12–C11–Se1	123.6(3)	121.70	119.11(14)	121.70
N13–C14–C17	121.8(3)	121.20	119.71(19)	121.10
C11–N10–C9	113.4(3)	112.80	112.22(16)	112.80
C11–N12–N13	120.4(3)	120.60	117.09(17)	120.60
C14–N13–N12	115.4(3)	119.10	118.96(17)	119.10
O23–N21–O22	122.6(4)	124.60	123.6(2)	124.60
O23–N21–C20	119.0(4)	117.70	118.4(2)	117.70
O22–N21–C20	118.4(4)	117.70	118.0(2)	117.70
C8–Se1–C11	83.76(16)	83.20	82.79(9)	83.20

### Cyclic voltammetry (CV)

**Figure 4:**
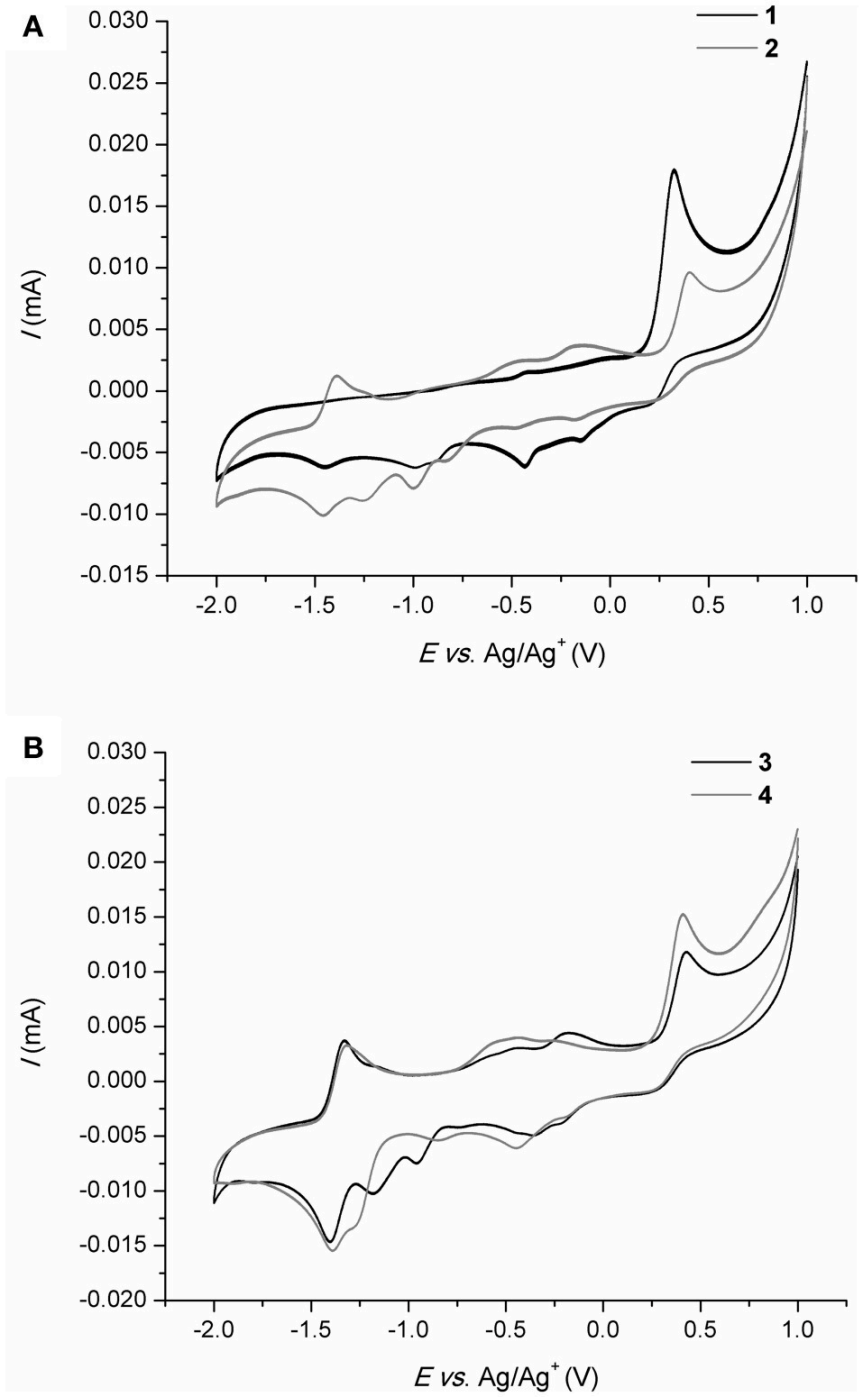
Cyclic voltammograms of **1** and **2 (A)** and **3** and **4 (B)**.

All investigated compounds were studied voltammetrically in DMSO using TBAP as supporting electrolyte. Results of CV study are given in Table 2. Examples of cyclic voltammograms of compounds **1–4** are given in Figure 4. In the investigated potential range (+1.0 to −2.0 V), the compounds from set 1 showed mainly one reduction and one oxidation peak. Reduction peak around −1.40 V is caused by reduction of imine group of the ligand. The peak at around +0.40 V can be attributed to the oxidation of chalcogen or C8 atoms. Both electrochemical processes are caused by chemical reaction (EC mechanism), as no peaks were observed in the reverse scan. For the oxidation peaks there were a few peaks of small intensities at the subsequent cathodic sweep as a result of decomposition of the oxidized species (Filipović et al., 2017). Cyclic voltammograms of nitro derivatives showed additional reduction and oxidation peaks. Reduction peak at around −1.20 V corresponds to reversible one-electron reduction of the radical anion of the nitro group which is commonly known in aprotic solvents (Silvester et al., 2006). Since the intensities of the reverse scan currents are decreased the mechanism of the reaction is also EC. Additional oxidation peak at around −1.35 V belongs to reversible one-electron oxidation of imine group. The oxidation peak is invisible for compounds from set 1 which means that the presence of strong electron withdrawing nitro group enables oxidation of the anion (Fry and Reed, 1969). The intensities of the reverse scan are increased by 20–30% implying the ECE nature of the reaction mechanism. Peak currents were correlated with the square root of scan rate (20–500 mV s^−1^) and the linear relationship was obtained which indicated diffusion controlled process on the electrode surface.

**Table 2:** Voltammetric characteristics of the the benzylidene-based (1,3-selenazol-2-yl)hydrazones^a^.

**Compound**	**${\text{E}}_{{\text{p}}^{\text{R}\left(\text{I}\right)\text{b}}}$**	***I*_p_/cv^1/2^^c^**	**${\text{E}}_{{\text{p}}^{\text{O}\left(\text{I}\right)}}$**	***I*_p_/cv^1/2^**	**${\text{E}}_{{\text{p}}^{\text{R}\left(\text{II}\right)}}$**	***I*_p_/cv^1/2^**	**${\text{E}}_{{\text{p}}^{\text{O}\left(\text{II}\right)}}$**	***I*_p_/cv^1/2^**	**${\text{E}}_{{\text{p}}^{\text{Ox}\left(\text{III}\right)}}$**	***I*_p_/cv^1/2^**	**${\text{E}}_{{\text{HOMO}}^{\text{d}}}$ (CV)**	***E*_HOMO_ (DFT)**
**1**	/	/	/	/	−1.45	2.1	/	/	+0.33	39.1	−4.90	−5.34
**1-Me**	/	/	/	/	−1.50	2.9	/	/	+0.28	38.4	−4.85	−5.20
**1-OMe**	/	/	/	/	−1.59	2.3	/	/	+0.31	26.6	−4.88	−5.30
**2**	−1.26	6.8	−1.27	4.1	−1.46	5.3	−1.39	12.7	+0.40	23.2	−4.97	−5.49
**2-Me**	−1.27	13.2	−1.23	2.3	−1.49	6.6	−1.39	13.9	+0.36	24.0	−4.93	−5.31
**2-OMe**	−1.25	11.3	−1.26	3.6	−1.47	10.6	−1.40	15.5	+0.43	23.0	−5.00	−5.43
**3**	−1.18	10.7	−1.20	4.2	−1.40	16.8	−1.33	22.2	+0.42	25.7	−4.99	−5.51
**3-Me**	−1.20	4.2	−1.19	5.4	−1.40	17.9	−1.33	4.3	+0.42	18.9	−4.99	−5.32
**3-OMe**	−1.21	13.1	−1.19	4.6	−1.44	14.9	−1.32	21.3	+0.42	20.2	−4.99	−5.45
**4**	−1.29	25.3	/	/	−1.38	3.5	−1.32	21.3	+0.41	35.8	−4.98	−5.46
**4-Me**	−1.27	25.3	/	/	−1.39	2.4	−1.32	19.4	+0.40	34.8	−4.97	−5.28
**4-OMe**	−1.30	26.2		/	−1.39	2.2	−1.31	18.9	+0.37	33.5	−4.94	−5.40

a In DMSO containing 0.1 M TBAP at v = 100 mV/s.

b E vs ferrocene/ferrocenium couple (Fc/Fc^+^) in V.

c In μA/mM(V/s)^1/2^;

d in eV. *E_HOMO_ = −4.8 – (E – E_Fc_), E_Fc_ = +0.23 V*.

### DFT and time-dependent-DFT calculations

**Figure 5:**
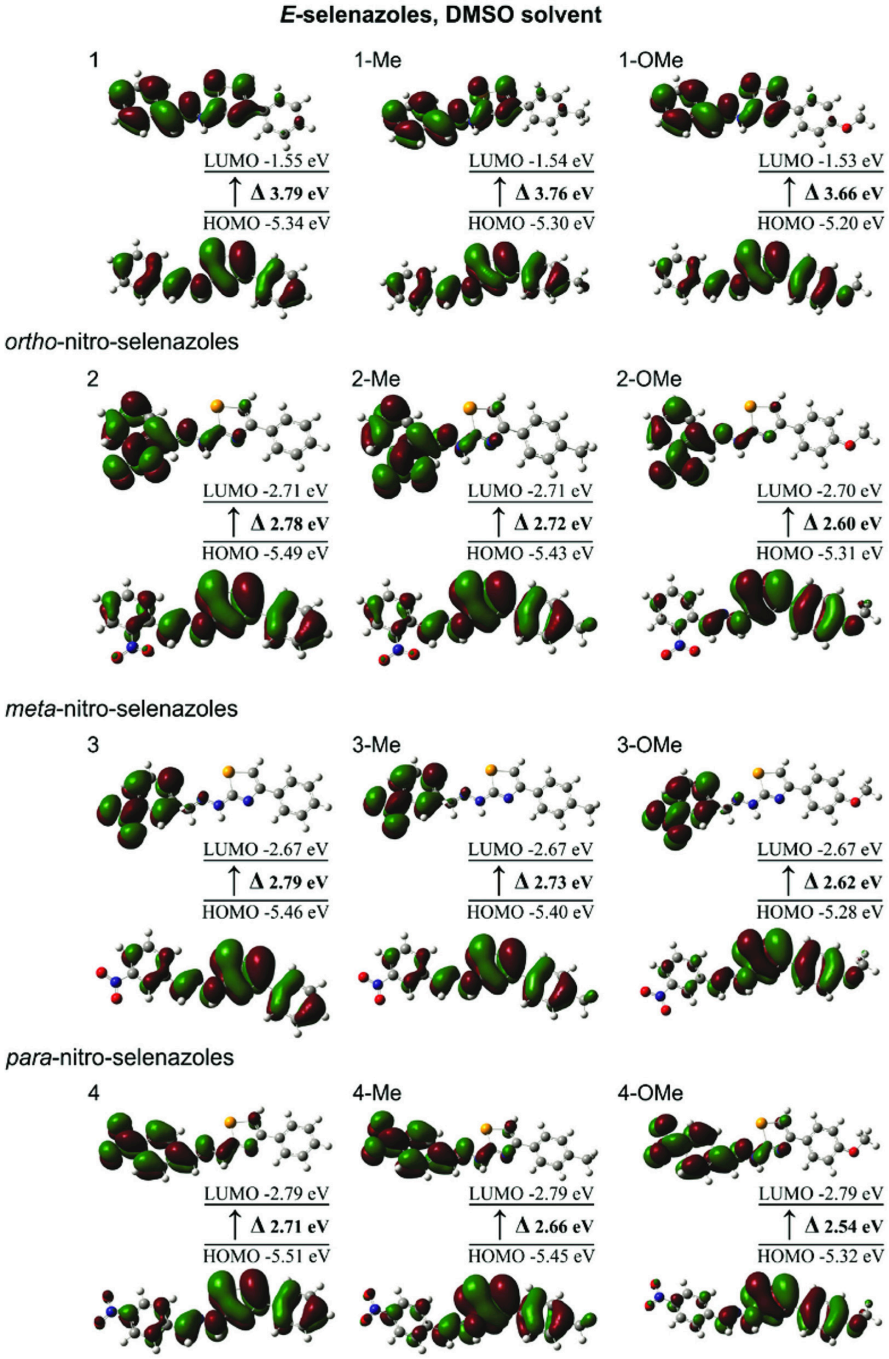
Molecular orbital plots and energy levels of the HOMO, the LUMO, and HOMO-LUMO transitions of the benzylidene-based (1,3-selenazol-2-yl) hydrazones in DMSO.

Electronic properties of investigated molecules were studied using calculated energy of HOMO and LUMO orbitals and HOMO–LUMO energy gap (*E*_gap_). All vertical excitation energies were computed using B3LYP/6-31G(d,p) optimized ground-state geometries in DMSO. Influence of substituents is estimated by comparing the calculated frontier molecular orbital energies (*E*_LUMO_, *E*_HOMO_) and *E*_gap_ (Table 3). Molecular orbital plots and energy levels of the HOMO, the LUMO and HOMO-LUMO transitions of investigated compounds in DMSO are depicted in Figure 5. The main difference between compounds from set 1 and nitro-substituted (1,3-selenazol-2-yl)hydrazones derives from the stabilization of LUMO in the presence of nitro group. Different positions of nitro group on the phenyl ring A cause certain changes in frontier molecular orbital energies. As it is well known, electron acceptor group, such as nitro group, adjacent to the aromatic ring decreases the electron density on the ring through a resonance withdrawing effect. If an acceptor is in a *para* or *ortho* position, certain stabilization can be expected through the corresponding resonance forms. The change in the position of the nitro group from *para* to *ortho* and *meta* destabilizes both HOMO and LUMO. A relatively small increase in HOMO orbital energies can be negligible. Destabilization of the LUMO by 0.1 eV when nitro substituent changes position from *para* to *ortho* or *meta*, leads to an increase of the energy gap. In all molecules with *para* and *ortho*-nitro substituents, the LUMO are mainly located on the aromatic rings A and hydrazone bridges. In the case of molecules containing the nitro group in *meta-*position, the LUMO are mainly located on the aromatic rings A with smaller participation of the hydrazone bridges. The HOMO are located on selenazole rings, phenyl rings B and hydrazone bridges (Figure 5). The presence of electron donating substituents (–Me and –OMe) on the phenyl rings B, destabilize HOMO and decrease the energy gap. Since –OMe group is stronger electron donating group in comparison to –Me group, selenazole analogs with OMe substituted phenyl rings B have the smallest energy gap.

**Table 3:** Calculated energies of the HOMO and LUMO orbitals and energy gap (in eV) for *E*-(1,3-selenazol-2-yl)hydrazones in DMSO obtained by TD/DFT method.

**Compound**	***E*_LUMO_**	***E*_HOMO_**	***E*_gap_**
**1**	−1.55	−5.34	3.79
**1**-**Me**	−1.54	−5.30	3.76
**1**-**OMe**	−1.53	−5.20	3.66
**2**	−2.71	−5.49	2.78
**2**-**Me**	−2.71	−5.43	2.72
**2**-**OMe**	−2.70	−5.31	2.61
**3**	−2.79	−5.51	2.72
**3**-**Me**	−2.79	−5.45	2.66
**3**-**OMe**	−2.79	−5.32	2.54
**4**	−2.67	−5.46	2.79
**4**-**Me**	−2.67	−5.40	2.73

### MAO A/B inhibition capacity

The main characteristics of neurodegenerative diseases are their complex etiology and late onset. Currently available therapies cannot stop progressive loss of function of neurons and can provide just symptomatic relief. There is an urgent need for discovery of novel chemical entities which can be used for the treatment of neurodegeneration (Grottelli et al., 2016). One of the strategies for the treatment of neurodegenerative diseases is targeting MAOs, in particular MAO B in case of Parkinson's disease. These are enzymes, joined to the outer membrane of mitochondria, which contain flavin adenine dinucleotide (FAD). Two MAO isoforms, A and B, are encoded by different genes. They have different distribution in tissues and specificity for their substrates and inhibitors. Well known MAO A substrates are serotonin, adrenaline and noradrenaline, while this isoform is irreversible inhibited by clorgyline and reversibly inhibited by moclobemide. MAO B substrates are β-phenethylamine and benzylamine, while irreversible inhibition is achieved by the MAO B selective antiparkinsonian drugs selegiline and rasagiline (Carradori and Silvestri, 2015). Notably, MAOs regeneration process can yield the oxidative stress or hydrogen peroxide, thus, inhibitors of MAOs might demonstrate an indirect antioxidative and neuroprotective effect (Ramsay, 2016).

With the well-known representative phenelzine or iproniazid, two hydrazine derivatives as non-selective MAO inhibitors are used as antidepressants in the class of MAO inhibitors. Based on that, numerous thiazol-2-ylhydrazones were developed to obtain isoform-selective MAO inhibitors (for review see Carradori and Silvestri, 2015), while related hydrazone-selenazoles have so far not been investigated in this context to best of our knowledge. Among thiazole derivatives, benzilydene-based (1,3-thiazol-2-yl)hydrazones were the most active. Further enhancement of their activity is achieved by the substitution at position 4 of the 1,3-thiazole nucleus. Additionally, presence of the aryl group at C4 of the 1,3-thiazole ring allows the correct orientation of the inhibitor inside the active site of enzyme close to the catalytic FAD aromatic cage (Oncü Can et al., 2018). Consequently, we investigated the MAO inhibition capacities of our structurally related benzilydene-based (1,3-selenazol-2-yl)hydrazones. In a pre-screening, we found most promising inhibition capacities for compounds **1** and **4** with percental MAO inhibition >70% at a concentration of 1 μM (Table 4), while substitution on B phenyl ring tend to results in reduced MAO inhibition potency for these two representatives (e.g., **1** vs. **1-Me** and **1-OMe**). Further more detailed characterization revealed nanomolar activity. The most potent compound **4**, bearing a *meta* nitro-group, showed IC_50_ values of 73 nM (95% CI = [58, 93]) and 258 nM (95% CI = [222, 301]) for MAO B and MAO A, respectively. Surprisingly, compound **1** demonstrated high MAO B preference (IC_50_ = 252 nM, 95% CI = [173, 365]) with no inhibition of MAO A (IC_50_ > 10,000 nM).

**Table 4:** Monoamine oxidase (MAO) A/B inhibition capacities of benzylidene-based (1,3-selenazol-2-yl)hydrazones.

**Compound**	**% Inhibition**^a^ **(at 1** μ**M)**	
	**MAO A**	**MAO B**
**1**	−6.8 ± 12.6	89.4 ± 1.5
**1-Me**	12.7 ± 3.5	23.4 ± 17.1
**1-OMe**	32.4 ± 3.3	33.0 ± 11.6
**2**	26.5 ± 3.7	64.8 ± 6.1
**2-Me**	17.2 ± 1.9	22.1 ± 8.7
**2-OMe**	28.0 ± 4.0	35.9 ± 11.2
**3**	32.7 ± 6.2	57.5 ± 11.4
**3-Me**	13.4 ± 2.4	7.4 ± 27.0
**3-OMe**	31.2 ± 1.6	23.8 ± 7.7
**4**	71.9 ± 3.5	96.3 ± 0.5
**4-Me**	22.1 ± 2.1	48.1 ± 2.9
**4-OMe**	49.4 ± 1.3	42.5 ± 19.0

a *Percental inhibition calculated from remained enzyme activity normalized to control (= 100%). Values are given as means ± standard deviations (n = 2, global fit)*.

### *In vitro* antioxidant capacity (AOC)

Since neurodegenerative diseases have complex and variable underlying mechanisms, novel requirements for efficient therapies include factors such as mitochondrial dysfunction, neuroinflammation, and especially oxidative stress which have been identified as major determinants for their progress and development. Consequently, an antioxidant drug development strategy for neurodegenerative diseases has been of paramount importance (Bautista-Aguilera et al., 2017).

Despite the fact that (thiazol-2-yl)hydrazones were investigated in great extent as potential MAO inhibitors and neurodegenerative drugs, only one recent study is dealing with their antioxidant properties (Carradori et al., 2018). On the other hand, results of comparative antioxidant study on pyridine-based analogs of compounds from set 1 and their sulfur isosters indicated greater free-radical scavenging activity of (selenazol-2-yl)hydrazones in DPPH assay (Filipović et al., 2017). To get deeper insight on mechanism of AOC of investigated compounds we investigated their radical scavenging activity, the oxygen radical absorption capacity and reduction ability was measured in a series of four *in vitro* tests (Table 5). The DPPH assay is well known because of its ease and convenience for testing of the free radical-scavenging activity of various synthetic compounds. When an antioxidant scavenges these stable free radical by hydrogen radical or electron donation the purple DPPH assay solutions decolorized. ORAC test assay detects decrease in fluorescence of fluorescein due to its oxidation by a radical formed by the breakdown of AAPH over time (Ou et al., 2001). Antioxidant suppresses this reaction by hydrogen atom transfer. Trolox, a water soluble vitamin E analog, serves as a positive control for quantification of antioxidant activity present by its normalization to equivalent Trolox units. Since the reducing power of a compound may be a good indication of its possible antioxidant activity, the reduction of Fe(III) to Fe(II) which results in Perl's Prusian blue colored complex formation (Oyaizu, 1986), as well as Mo(VI) to Mo(V) reduction with formation of green colored phosphate/Mo(V) complex (Prieto et al., 1999), were investigated in the presence of the tested compounds.

**Table 5:** Antioxidant capacity of investigated benzylidene-based (1,3-selenazol-2-yl)hydrazones and the standard.

**Compound**	**IC_50_ (μM)^a^**	**EC**_**50**_ **(**μ**M)^b^**		**TE**
	**DPPH**	**TAOC**	**TRP**	**ORAC**
**1**	8.63 ± 1.66	648 ± 55	990 ± 92	0.75 ± 0.05
**1-OMe**	54.26 ± 4.54	548 ± 53	806 ± 85	0.74 ± 0.06
**1-Me**	45.06 ± 6.53	603 ± 61	966 ± 88	0.65 ± 0.07
**2**	21.9 ± 5.6	357 ± 41	390 ± 48	0.82 ± 0.07
**2-OMe**	40.5 ± 3.8	433 ± 39	560 ± 53	0.82 ± 0.07
**2-Me**	20.2 ± 4.3	416 ± 45	470 ± 41	0.77 ± 0.05
**3**	173.5 ± 11.6	325 ± 38	420 ± 45	0.90 ± 0.06
**3-OMe**	298.1 ± 14.8	632 ± 50	488 ± 46	0.86 ± 0.07
**3-Me**	151.6 ± 10.1	571 ± 45	480 ± 50	0.83 ± 0.05
**4**	44.8 ± 2.3	433 ± 43	376 ± 51	0.92 ± 0.08
**4-OMe**	79.2 ± 3.9	645 ± 53	398 ± 44	0.92 ± 0.07
**4-Me**	40.1 ± 4.7	579 ± 51	495 ± 48	0.92 ± 0.07
vitamin C	79.1 ± 1.8	140 ± 10	155 ± 39	0.97 ± 0.07

a IC_50_, concentration providing 50% of radicals scavenging activity.

b *EC_50_, Concentration providing 0.500 of absorbance; The lower IC_50_ or EC_50_ value the higher the antioxidant capacity; Values are given as means ± standard deviations (n = 3)*.

In our previous study pyridine-based analogs (HLSe^1^, HLSe^2^ and HLSe^3^) of compounds from set 1 were tested in DPPH test and the activities were compared with vitamin C (Filipović et al., 2017). Unsubstituted derivative HLSe^1^ appeared to be the most active, while addition of –OMe and –Me substituents resulted in less active species. The same trend was observed in the case of their benzylidene-based analogs from set 1 (Table 5), but with a significant difference in terms of activity. All three derivatives showed significantly stronger free-radical scavenging activity than vitamin C, especially **1**, which was an order of magnitude more active than the standard.

Addition of nitro group on the phenyl ring A reduced the activity of **2**, **4** and **4-OMe** to some extent, while this effect was the strongest for compounds from set 3 which is the only series of compounds with lower activity than vitamin C. In all three sets of compounds containing nitro group, the order of activities changed from H > Me > OMe (set 1) to Me> H > OMe (sets 2–4), but activity of non-substituted and Me-derivatives was almost the same in the case of *ortho* and *para* substitution. Compounds **2-OMe**, **2-Me** and **4-Me** are the only nitro group-containing compounds which showed better activity than their non-substituted analogs.

To the best of our knowledge ORAC, TAOC, and TRP tests were performed for the first time for evaluation of AOC of some 1,3-selenazole based compounds. While observed activities in TAOC and TRP tests were negligible (Table 5), activities of all investigated compounds were greater than vitamin C in ORAC test. Again, the series without nitro substituent showed the best activity, but **1-Me** appeared to be the most active compound. Methyl derivatives showed the best activities in all three series. In contrast to DPPH test, compounds having nitro group in *ortho* position showed the weakest activities. Based on results presented in Table 5 it was possible to establish simple structure-activity relationship. The order of activities of compounds from the sets is: set 1 > set 2 > set 3 > set 4, while in each set the order of activity is Me > OMe > H.

### Antiproliferative effects

While 1,3-thiazoles are well known by their anticancer activity (Ayati et al., 2015), their selenium analogs have been studied in much less extent. To the best of our knowledge, there are only two systematic anticancer activity studies of 1,3-selenazoles (Zaharia et al., 2013; Zhao et al., 2013). Herein we performed the *in vitro* antiproliferative activity of the benzilydene-based (1,3-selenazol-2-yl)hydrazones on the following human solid tumor cell lines: A549, HBL-100, HeLa, SW1573, T-47D and WiDr and one normal human cell line BJ-hTert (Table 6).

**Table 6:** Antiproliferative activities of benzylidene-based (1,3-selenazol-2-yl)hydrazones.

**Compound**	**GI**_**50**_ **(**μ**M)**						
	**A549**	**HBL-100**	**HeLa**	**SW1573**	**T-47D**	**WiDr**	**BJ-hTert**
**1**	28.0 ± 7.1	91 ± 12	41.0 ± 9.5	24 ± 8	6.4 ± 0.9	4.8 ± 0.8	n.d.
**1-Me**	n.d.^a^	n.d.	n.d.	47 ± 3	n.d.	n.d.	n.d.
**1-OMe**	n.d.	n.d.	n.d.	42.0 ± 7.7	n.d.	n.d.	n.d.
**2**	4.9 ± 1.5	21.0 ± 8.6	6.0 ± 0.7	5.3 ± 0.6	12.0 ± 1.5	5.0 ± 0.9	n.d.
**2-Me**	8.8 ± 2.2	95.0 ± 8.1	7.4 ± 1.4	4.6 ± 1.2	13.0 ± 1.3	8.2 ± 1.0	n.d.
**2-OMe**	22.0 ± 5.6	n.d.	49 ± 12	46 ± 10	36 ± 8.6	22.0 ± 2.3	n.d.
**3**	6.1 ± 0.8	28.0 ± 0.8	24.0 ± 7.1	16.0 ± 4.8	52 ± 13	40.0 ± 0.6	n.d.
**3-Me**	44 ± 15	60.0 ± 9.5	31.0 ± 4.0	18.0 ± 4.5	46.0 ± 9.7	55 ± 19	n.d.
**3-OMe**	52 ± 1	n.d.	26 ± 2	8.4 ± 0.2	67.0 ± 2.2	n.d.	n.d.
**4**	16.0 ± 3.5	n.d.	31.0 ± 8.5	23.0 ± 1.2	6.3 ± 1.1	6.9 ± 1.3	n.d.
**4-Me**	n.d.	n.d.	n.d.	n.d.	n.d.	n.d.	n.d.
**4-OMe**	16.0 ± 5.3	47 ± 13	33 ± 10	26 ± 17	66 ± 17	n.d.	n.d.
5-fluorouracil	n.d.	4.0 ± 0.7	15.0 ± 4.7	4.6 ± 1.5	47 ± 18	49.0 ± 6.7	5.5 ± 0.5

a *n.d, not determined (GI_50_ > 100 μM)*.

In our study, **1** showed moderate activity (GI_50_ = 10–100 μM) when tested against A549, HBL-100, HeLa and SW1573 cell lines, and good activity (GI_50_ = 1–10 μM) against T-47D and WiDr cell lines. Substitution on B phenyl ring reduced activity in set 1 since **1-Me** and **1-OMe** were inactive (GI_50_ > 100 μM) against five cell lines. In contrast, the introduction of a nitro group in the phenyl ring A significantly influenced the antiproliferative activity of **2**–**4** against A549, HBL-100, HeLa and SW1573 cells. The same trend was observed for nitro analogs of **1-Me** and **1-OMe** on all six cell lines with one exception. Namely, **4-Me** remained inactive against all six cell lines similarly to **1-Me**. The most potent compounds were **2** and **2-Me**, which showed good activity against A549, HeLa, SW1573 and WiDr cells. However, GI_50_ values obtained on T-47D cell line were very close to 10 μM. Also, a good activity was noticed for **3** on A549 cells, **3-OMe** on SW1573 cells and **4** against T-47D and WiDr cells. Some of the activities exhibited by benzylidene-based (1,3-selenazol-2-yl)hydrazones were comparable or even better than values obtained for positive control 5-fluorouracil, the blockbuster anticancer drug. It is worth to mention that in general all investigated compounds showed selectivity toward tumor cell lines, since GI_50_ values for non-transformed BJ-hTert cell line were not reached in the range of applied concentrations (up to 100 μM). Contrary, cytotoxic activity on BJ-hTert cell of positive control 5-fluorouracil was in low micromolar concentration range.

### Prediction of absorption, distribution, metabolism, and excretion (ADME) parameters and pan assay interference compounds (PAINS) evaluation

One of the main reasons for the frequent failure to develop drug-like candidates is the risk of unwanted adverse side effects and poor bioavailability in *in vivo* assays. To reduce the time and cost of analysis of molecules without desirable pharmacokinetic or pharmacodynamic profiles many *in-silico* platforms for evaluation of number of physicochemical, pharmacokinetics and medicinal chemistry properties have been developed (Muller et al., 2017). The *in-silico* ADME profiles of the most active compounds regarding MAO inhibition (**1** and **4**) and antiproliferative activity (**2** and **2-Me**) were assessed through robust SwissADME program and results are presented in Table 7. All compounds shown desirable Lipinski rule principles like MW ≤ 500, number of atoms which act as hydrogen bond acceptors ≤ 10, number of hydrogen bond donors ≤ 5 and 1-octanol / water partition coefficient (logP_o/w_ ≤ 5) values (Lipinski et al., 2001). Other physicochemical properties of the most active compounds, such as number of rotatable bonds (≤ 10), molar refractivity (from 40 to 130) and topological polar surface area (TPSA ≤ 140 Å^2^), were also found within the acceptable range. All compounds are predicted to be highly absorbed by gastrointestinal (GI) system after oral administration, while some of them are likely to inhibit cytochrome P450 gene isoforms (i.e. CYP1A2, CYP219). One of the most important parameters, a fundamental pre-requisite for potential central nervous system (CNS) drugs, is blood-brain barrier (BBB) permeation (Pajouhesh and Lenz, 2005). SwissADME predicted that **4**, which showed MAO B inhibition in a nanomolar concentration range, would not cross BBB passively, while **1** is expected to be BBB accessible. However, based on Lipinski's rules for CNS drugs (Pajouhesh and Lenz, 2005; Fernandes et al., 2016), **4** would be likely active in the CNS. Relevant strategies for selection of molecules with preferred drug-like profiles examined by SwissADME indicate that the most active compounds represent drug candidates since they possess important functional groups and bioavailability. Finally, according to a recently published editorial by Aldrich et al. (Aldrich et al., 2017), in order to remove suspicion of artificial activity, in addition to SwissADME the compounds have been evaluated by ZINC PAINS Pattern Identifier (Sterling and Irwin, 2015). Applied algorithms did not report our compounds as potential PAINS or covalent inhibitors.

**Table 7:** Pharmacological profiles, medicinal chemistry principles and lead-likeness properties of compounds **1**, **4**, **2** and **2-Me**.

	**1**	**4**	**2**	**2-Me**
**PHYSICOCHEMICAL PROPERTIES**				
Molecular weight	326.25	371.25	371.25	385.28
#Heavy atoms	20	23	23	24
#Aromatic heavy atoms	17	17	17	17
Fraction Csp^3^	0	0	0	0.06
#Rotatable bonds	4	5	5	5
#H-bond acceptors	2	4	4	4
#H-bond donors	1	1	1	1
Molar Refractivity	84.09	92.91	92.91	97.88
Topological Polar Surface Area (TPSA-Å^2^)	37.28	83.1	83.1	83.1
ClogP_o/w_	2.07	1.54	1.54	1.82
**PHARMACOKINETICS**				
GI absorption	++^a^	++	++	++
BBB permeant	+^b^	–^c^	–	–
Pgp substrate	+	–	–	–
CYP1A2 inhibitor	–	+	+	+
CYP2C19 inhibitor	–	+	+	+
CYP2C9 inhibitor	–	–	–	–
CYP2D6 inhibitor	+	–	–	–
CYP3A4 inhibitor	–	–	–	–
log Kp (cm/s)	−5.98	−6.38	−6.38	−6.2
**DRUGLIKENESS**				
Lipinski #violations	0	0	0	0
Ghose#violations	0	0	0	0
Veber#violations	0	0	0	0
Egan #violations	0	0	0	0
Muegge#violations	0	0	0	0
Bioavailability score	0.55	0.55	0.55	0.55
**MEDICINAL CHEMISTRY**				
PAINS #alerts	0	0	0	0
Leadlikeness	Yes	Yes	Yes	Yes
Synthetic accessibility	3.63	3.62	3.71	3.81

a ++ high;

b + activity;

c *– no activity*.

### Docking study

Most drugs on the market were developed according to “one-target-one-disease” philosophy (Strebhardt and Ullrich, 2008) and despite notable successes of this approach, especially with single gene disorders, multifactorial diseases such as cancer still remain inadequately treated (Talevi, 2015). However, there are many examples of approved anticancer drugs, initially developed as single-targeting, but actually multi-targeting agents (Frantz, 2005; Yildirim et al., 2007). There is growing evidence that treatment of complex disorders, such as neurodegenerative disorders and cancer, is more likely to be effective through simultaneous modulation of multiple targets, making multi-target paradigm a relevant issue in the drug discovery process. Because of all mentioned above, it is important to study multi-targeting properties of novel bioactive compounds at the very beginning of their development in order to get insight about their ability to act against complex diseases by modulating multiple targets. Among other methods for target identification, the docking studies showed their significance in recent years (Ferreira et al., 2015). In this work, we tested the binding capacities of compounds that had the strongest inhibition capacity to MAO B (**1** and **4**) to also bind into the small conductance calcium-activated channel protein 1 (KCNN1), since this is a novel target for the treatment of neurological diseases through activation (Dolga et al., 2014). Also, for the most active compounds in antiproliferative screening (**2** and **2-Me**) docking to cancer related proteins, eukaryotic translation factor 4E (EIF4E) (Lu et al., 2016) and 5′-nucleotidase (5-NT) (Frasson Corbelini et al., 2015) was performed. The compounds studied had stronger calculated binding scores than known inhibitors, except for 5-NT where they were within 1 kcal/mol. The results are shown in Table 8, with co-crystallized ligands' values underlined.

**Table 8:** Docking scores (kcal/mol) for compounds and co-crystallized ligands in neurodegenerative and cancer related proteins.

**Neurodegenerative targets**	**5wbx/KCNN1**	**4crt/MAO B**
**1**	−8.5	−10.3
**4**	−8.4	−10.7
lig5wbx	–*7.7*	−8.1
lig4crt	−6.4	–*8.4*
**Cancer targets**	1ipb/EIF4E	4h2b/5-NT
**2-Me**	−10.0	−9.2
**2**	−9.5	−9.0
lig1ipb	–*9.3*	−10.3
lig4h2b	−9.7	–*9.9*

*Scores for co-crystallized ligands are shown in italic*.

**Figure 6:**
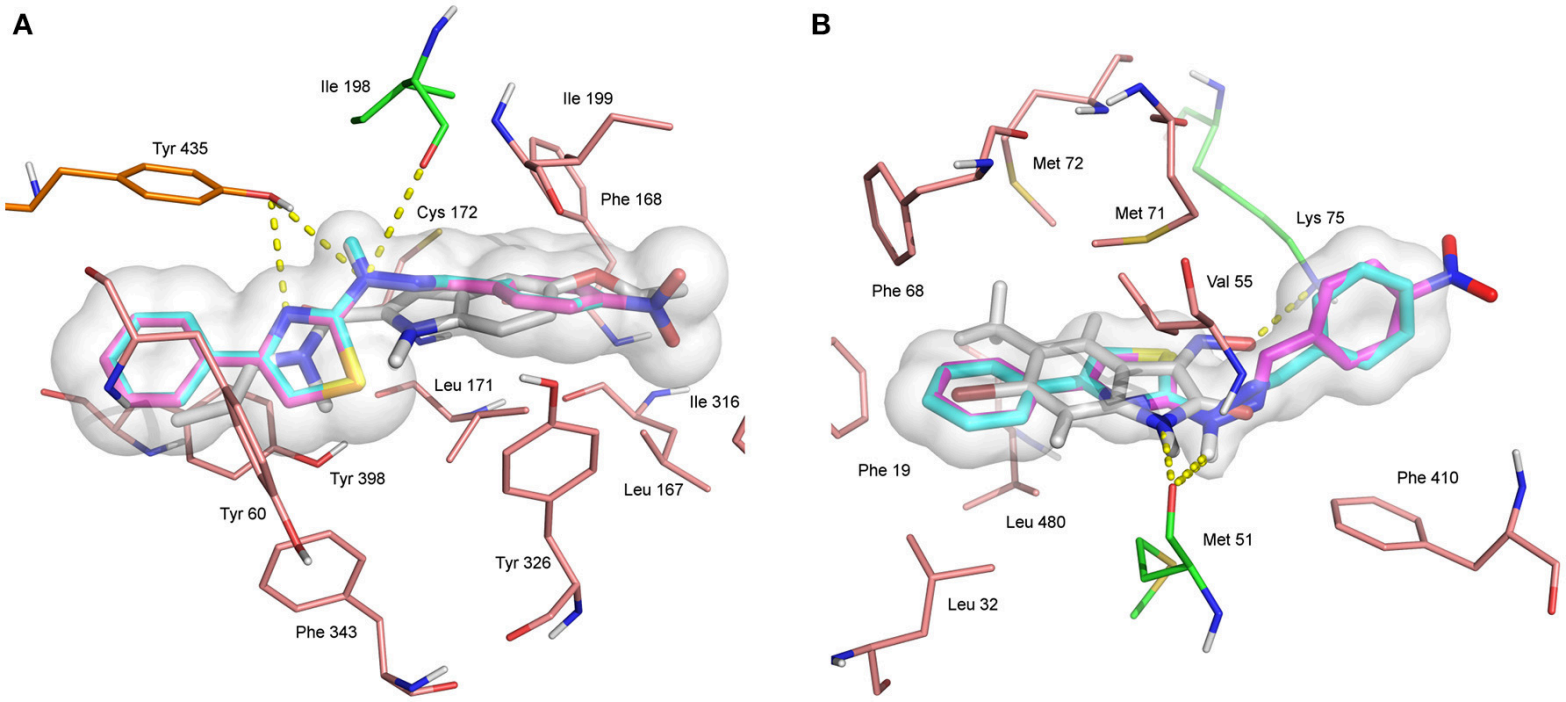
**(A)** Binding site of MAO B in white with co-crystallized ligand ASS234 ((*E*)-*N*-methyl-*N*-[[1-methyl-5-[3-[1-(phenylmethyl) piperidin-4-yl]propoxy]indol-2-yl]methyl]prop-1-en-1-amine). **(B)** Binding site of KCNN1 small conductance calcium-activated potassium channel protein 1 in white with co-crystallized ligand AJY; (3*Z*)-6-bromo-3-(hydroxyimino)-5-methyl-1,3-dihydro-2H-indol-2-one. In each case compounds **1** in cyan and **4** in magenta. Residues forming interactions shown in stick, with hydrophobic interaction groups shown in pink, electrostatic interaction in green, and both hydrophobic and electrostatic in orange. Hydrogen bonds shown as dashed lines; nitrogen in blue, oxygen in red, sulfur and selenium in yellow.

In addition, the results show that compounds **1** and **4** have good interactions inside the binding site of MAO B, as seen in Figure 6A. It can be seen that **1** and **4** have a near perfect overlap inside the binding site and they make strong hydrophobic and electrostatic interactions with residues in the binding site. They also have a binding pose similar to that of the known inhibitor ASS234 (Bautista-Aguilera et al., 2017). Figure 6B shows that the co-crystallized ligand and both compounds **1** and **4** donate a hydrogen bond to residue Met 51 of the channel protein KCNN1. In addition, AJY receives a hydrogen bond from Lys 75. Hydrophobic residues participating in the binding are Phe 19, Val 55, Phe 68, Met 71, Met 72, Phe 140, and Leu 480.

**Figure 7:**
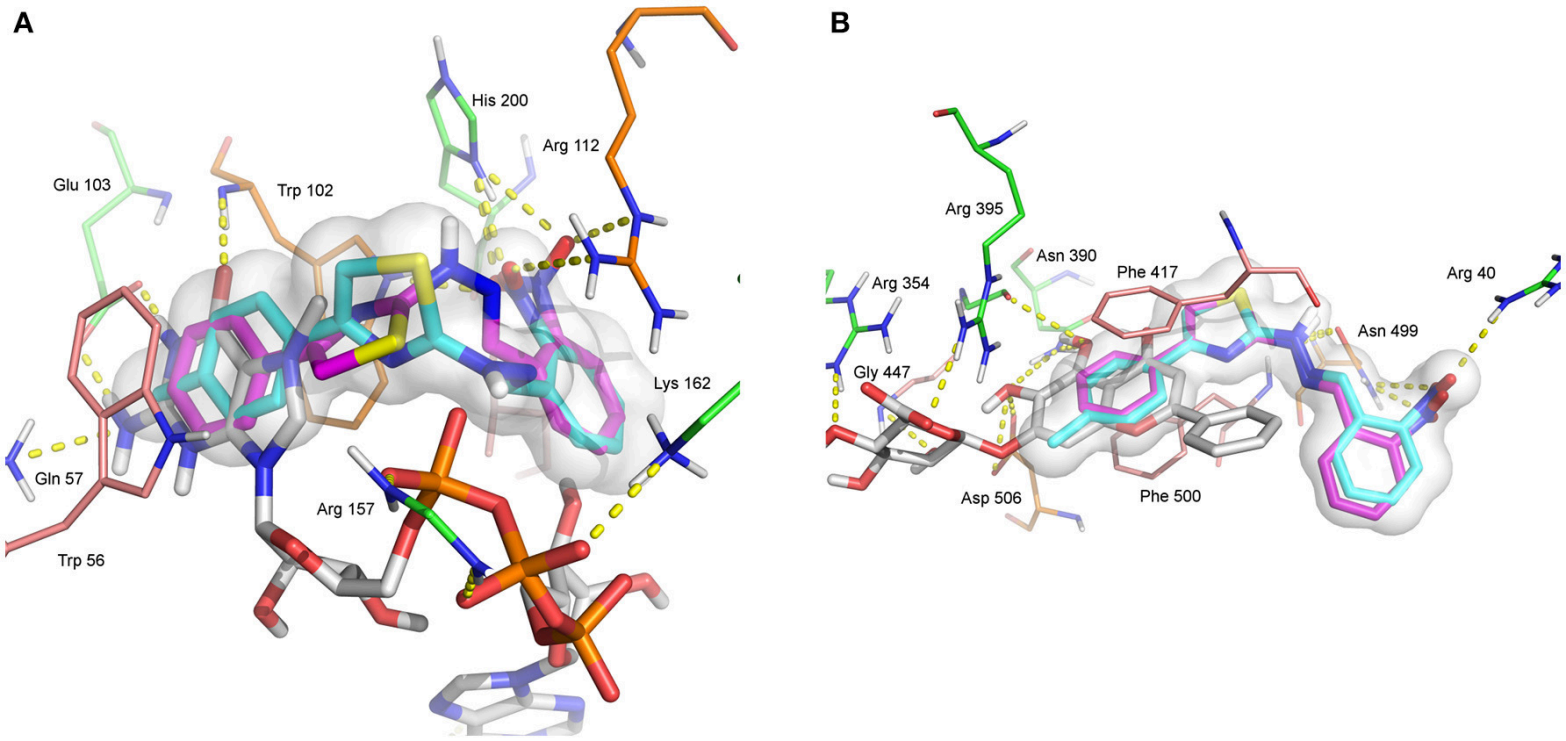
**(A)** Binding site of eukaryotic translation factor 4E in white with co-crystallized ligand GTA; P1-7-methylguanosine-P3-adenosine-5′,5′-triphosphate. **(B)** Binding site of 5′ nucleotidase in white with co-crystallized ligand 0XE; 5,6-dihydroxy-4-oxo-2-phenyl-4H-chromen-7-yl beta-D-glucopyranosiduronic acid; Baicalin. In each case compounds **2-Me** in cyan and **2** in magenta. Residues forming interactions shown in stick, with hydrophobic interaction groups shown in pink, electrostatic interaction in green, and both hydrophobic and electrostatic in orange. Hydrogen bonds shown as dashed lines; nitrogen in blue, oxygen in red, sulfur and selenium in yellow.

Figure 7A shows that both compounds **2-Me** and **2** receive hydrogen bonds from residues Trp 102, Arg 112, and His 200 from the binding site of EIF4E. Residues Trp 102 and Arg 112 participate also in π-π (as does Trp 56) and cation-π interactions, respectively, with the ligands. In addition, GTA participates in hydrogen bonding with Gln 57, Trp 102, Glu 103, Arg 157, and Lys 162. Phe 417 and Phe 500 from the binding site of 5-NT participate in π-π contacts with all ligands, as it can be seen in Figure 7B. Arg 40 and Asn 499 donate hydrogen bonds to both **2-Me** and to **2**. Asn 499 and Asp 506 also participate in nonpolar contacts to the ligands.

## Conclusions

Study of compounds from focused library of 12 benzilydene-based (1,3-selenazol-2-yl)hydrazones in screening on MAO B inhibition revealed that **1** and **4** possess IC_50_ values in nanomolar concentration range. Docking studies showed that KCCN1 is additional target for **1** and **4**, which indicates their possible multi-targeting properties for the treatment of neurodegenerative disorders. Antiproliferative activity screening indicates that **2** and **2-Me** are the most potent anticancer agents among investigated compounds with better activity than that of the positive control 5-fluorouracil. Docking studies point to 5-NT and EIF4E as possible cancer-related targets. All investigated compounds showed significant antioxidant activities, better than vitamin C in DPPH and ORAC assays. To conclude, our findings highlight the pharmacophore suitability of benzylidene-based (1,3-selenazol-2-yl)hydrazones as novel MAO B/KCNN1 targeting compounds with excellent antioxidative properties. This class also possess antiproliferative activity which may be attributed to their strong binding to cancer related targets 5-NT and EIF4E. Our further investigation will be focused on experimental work in order to confirm multi-targeting hypothesis.

## Author contributions

NF designed the study and drafted the manuscript; NF and TT analyzed and interpreted the data; HE performed synthesis and characterization of compounds; MN performed antioxidant-related assays; AL performed CV experiments and participated in analysis and interpretation of the data; AV performed X-ray crystallographic analysis; JP performed anticancer related experiments and participated in analysis and interpretation of the data; ID, SG, and AG-S performed *in-silico* studies; SH performed enzyme inhibition assays and HS contributed to discussion and critically revised the manuscript. All authors read and approved the submitted version.

### Conflict of interest statement

The authors declare that the research was conducted in the absence of any commercial or financial relationships that could be construed as a potential conflict of interest.
