# Supplementary material for: Selenazolyl-hydrazones as Novel Selective MAO Inhibitors With Antiproliferative and Antioxidant Activities: Experimental and In-silico Studies
Source: Front Chem. 2018 Jul 3;6:247. doi: 10.3389/fchem.2018.00247 (PMC6037691; doi:10.3389/fchem.2018.00247)
Supplement: Supplementary file 1 [file Data_sheet_1.docx]

Supplementary Material

Selenazolyl-hydrazones as Novel Selective MAO Inhibitors with Antiproliferative and Antioxidant Activities: Experimental and
*In-silico* Studies

**Hana Elshaflu^1^, Tamara R. Todorović^2^, Milan Nikolić^2^, Aleksandar Lolić^2^,
Aleksandar Višnjevac^3^, Stefanie Hagenow^4^, José M. Padrón^5^, Alfonso T. García-Sosa^6^,
Ivana S. Djordjević^7^, Sonja Grubišić^7^, Holger Stark^4^, Nenad R. Filipović^8,*^**

*** Correspondence:** Nenad R. Filipović: nenadf.chem@gmail.com

^1^Faculty of Technology and Metallurgy, University of Belgrade, Belgrade, Serbia

^2^Faculty of Chemistry, University of Belgrade, Belgrade, Serbia

^3^Physical Chemistry Division, Ruđer Bošković Institute, Zagreb, Croatia

^4^Institute of Pharmaceutical and Medicinal Chemistry, Heinrich Heine University Düsseldorf, Düsseldorf, Germany

^5^Instituto Universitario de Bio-Orgánica “Antonio González”, Universidad de La Laguna, Tenerife, Spain

^6^Institute of Chemistry, University of Tartu, Tartu, Estonia

^7^Institute of Chemistry, Technology and Metallurgy, University of Belgrade, Belgrade, Serbia

^8^Faculty of Agriculture, University of Belgrade, Belgrade, Serbia

# Supplementary Figures and Tables

| **Figure S1.** | Optimized geometries of benzylidene-based (1,3-selenazol-2-yl)hydrazones in the gas phase obtained with DFT/B3LYP/6-31G(d,p) method ........................................................................................................ | 2 |
| --- | --- | --- |
| **Figures S2.-S41.** | 1D and 2D NMR spectra of twelve benzylidene-based (1,3-selenazol-2-yl)hydrazones in DMSO-*d*_6_ ..................................................................... | 3-22 |
| **Table S1.** | Crystallographic data for **4-Me** and **4-OMe** .............................................. | 23 |
| **Table S2.** | Angles between the selenazole ring least square plane and phenyl rings least square planes .................................................................................. | 23 |


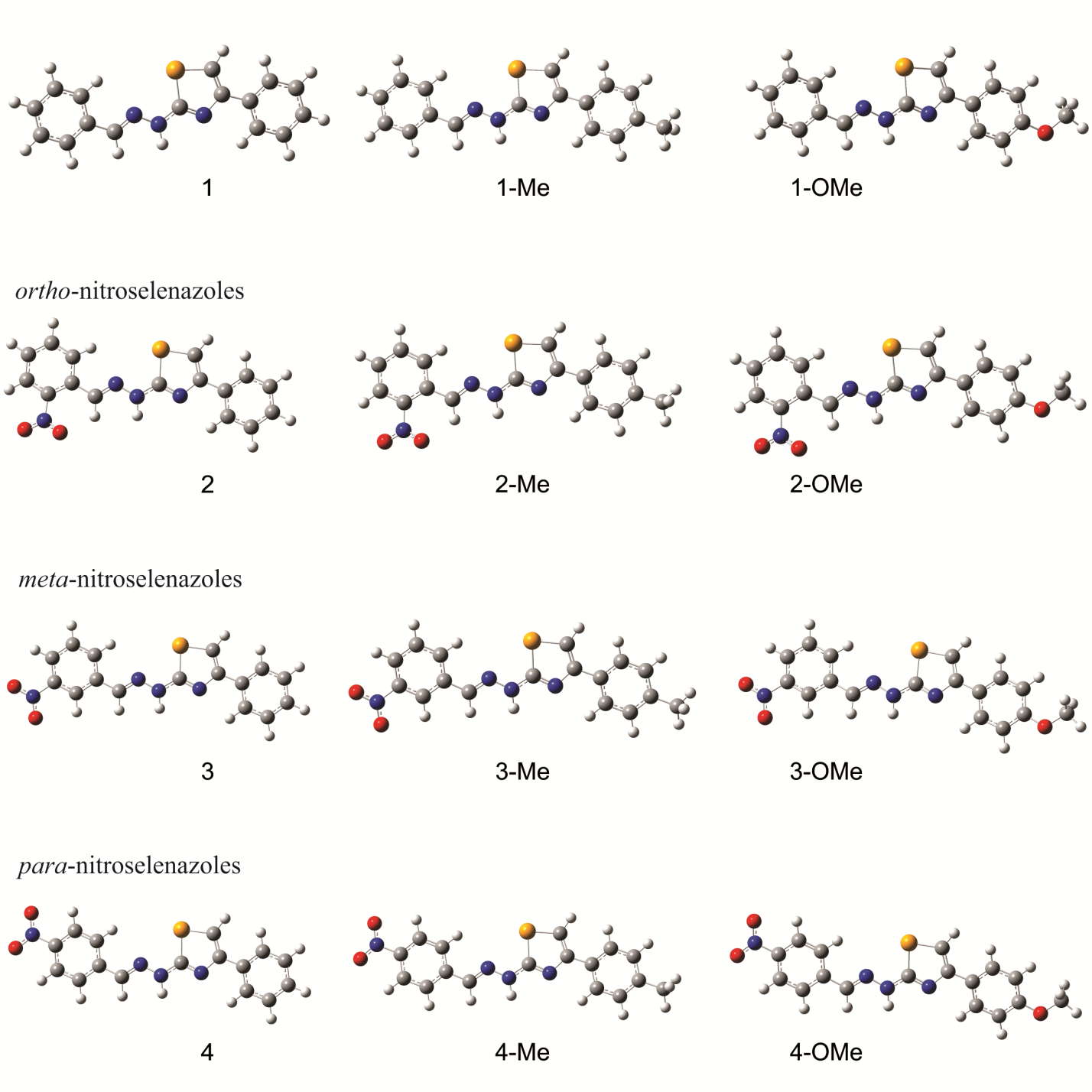


**Supplementary Figure S1.** Optimized geometries of benzylidene-based (1,3-selenazol-2-yl)hydrazones in the gas phase obtained with DFT/B3LYP/6-31G(d,p) method.


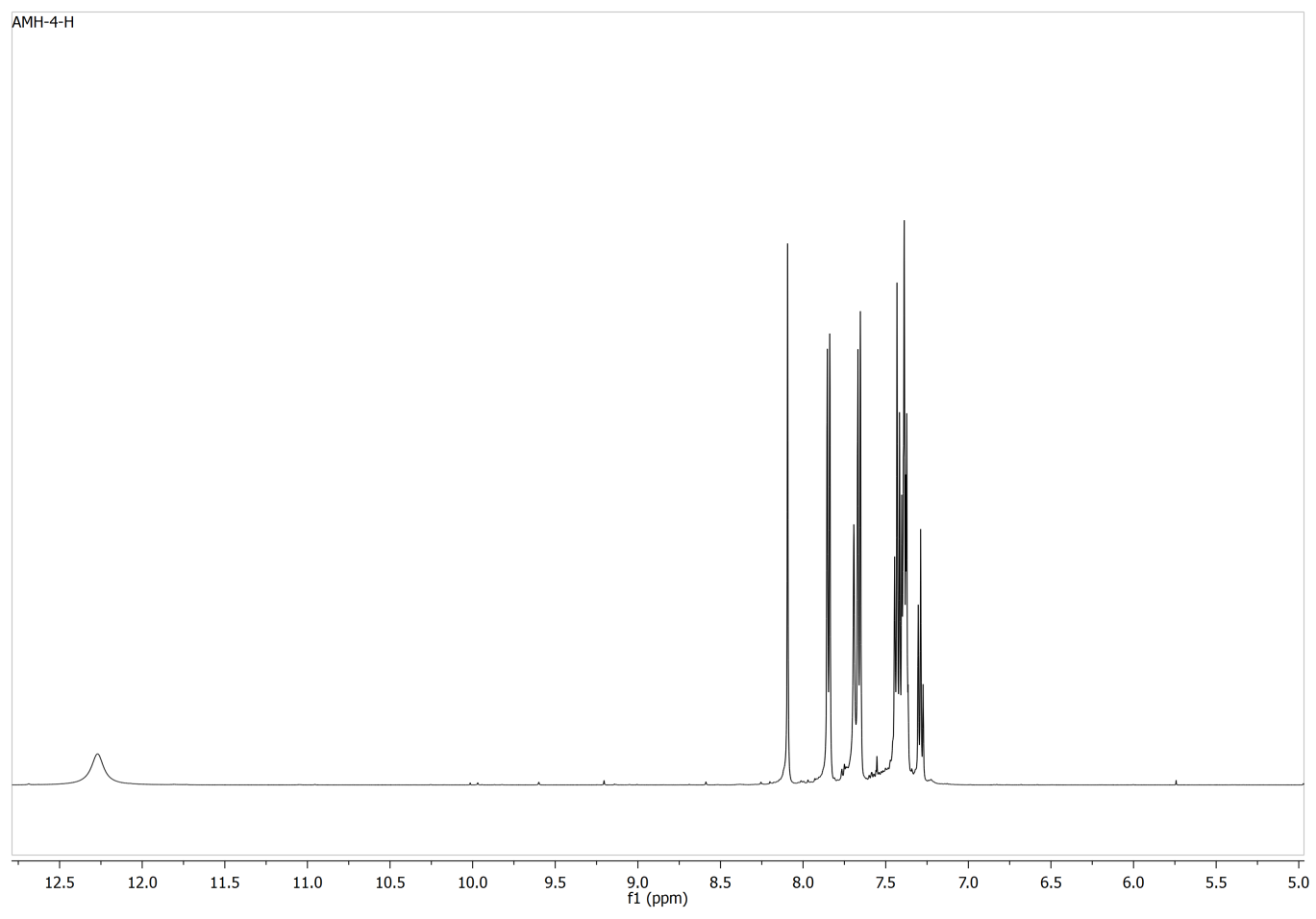


**Supplementary Figure S2.** ^1^H NMR spectrum of **1** in DMSO-*d_6_*.

**
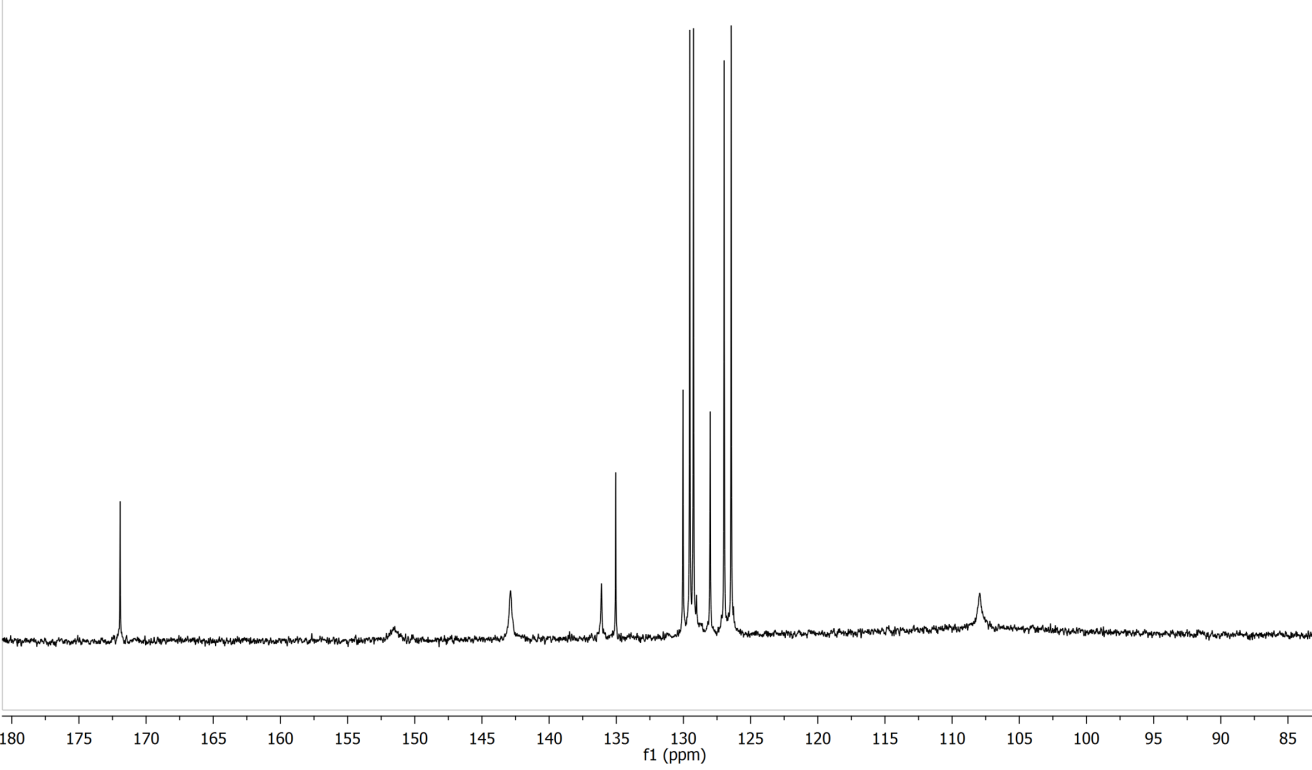
**

**Supplementary Figure S3.** ^13^C NMR spectrum of **1** in DMSO-*d_6_*.

**
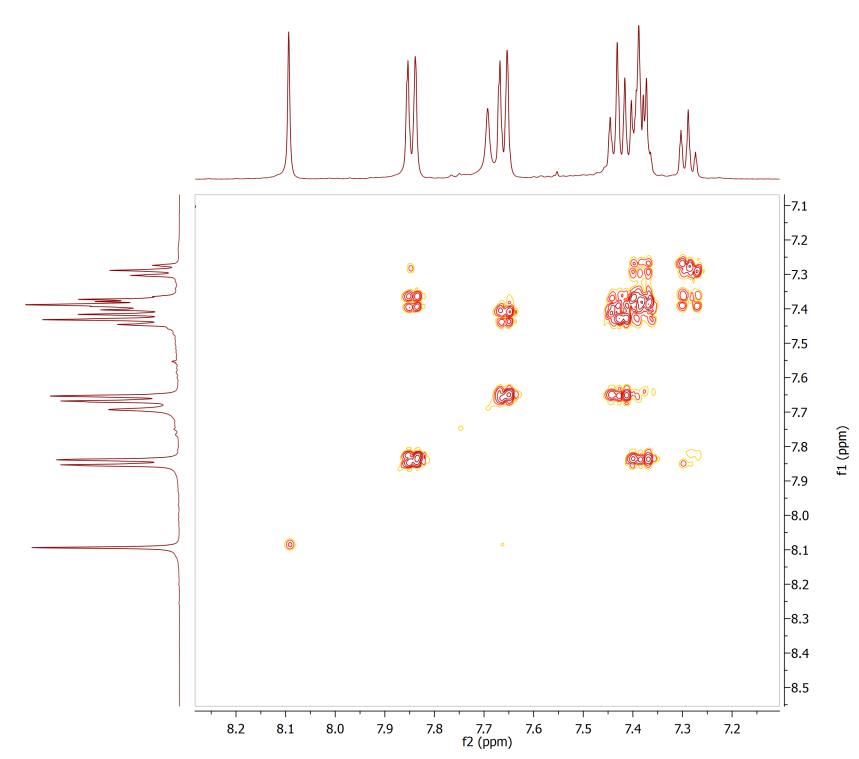
**

**Supplementary Figure S4.** COSY spectrum of **1** in DMSO-*d_6_*.

**
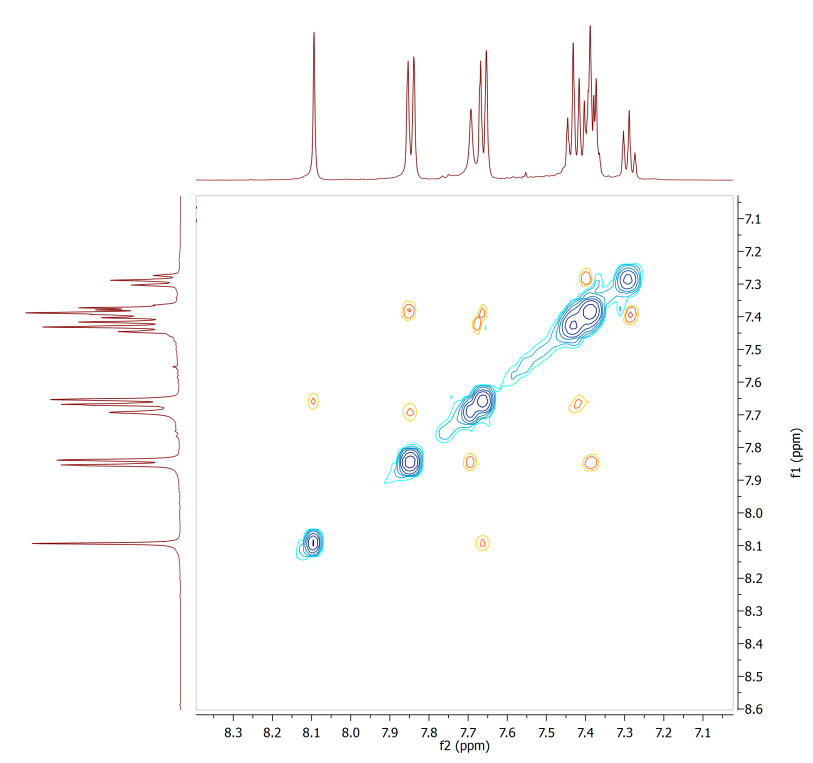
**

**Supplementary Figure S5.** NOESY spectrum of **1** in DMSO-*d*_6_.


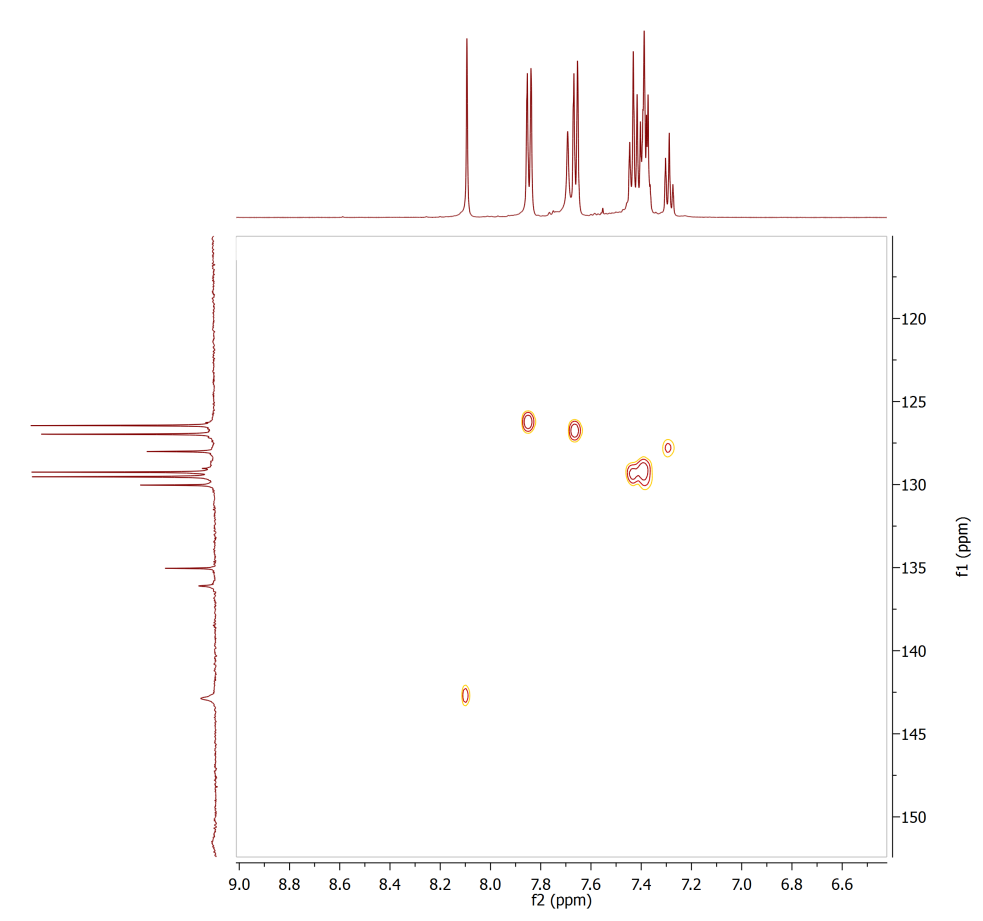


**Supplementary Figure S6.** ^1^H−^13^C HSQC NMR spectrum of **1** in DMSO-*d*_6_*.*

**
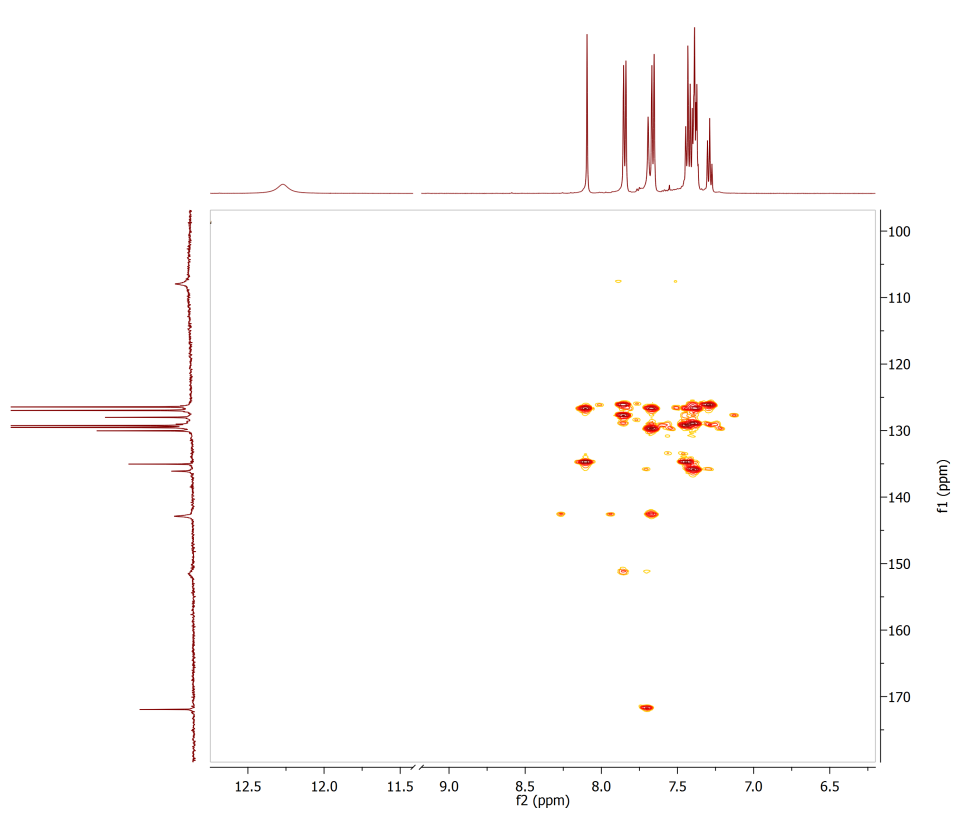
**

**Supplementary Figure S7.** ^1^H−^13^C HMBC NMR spectrum of **1** in DMSO-*d*_6_*.*

**
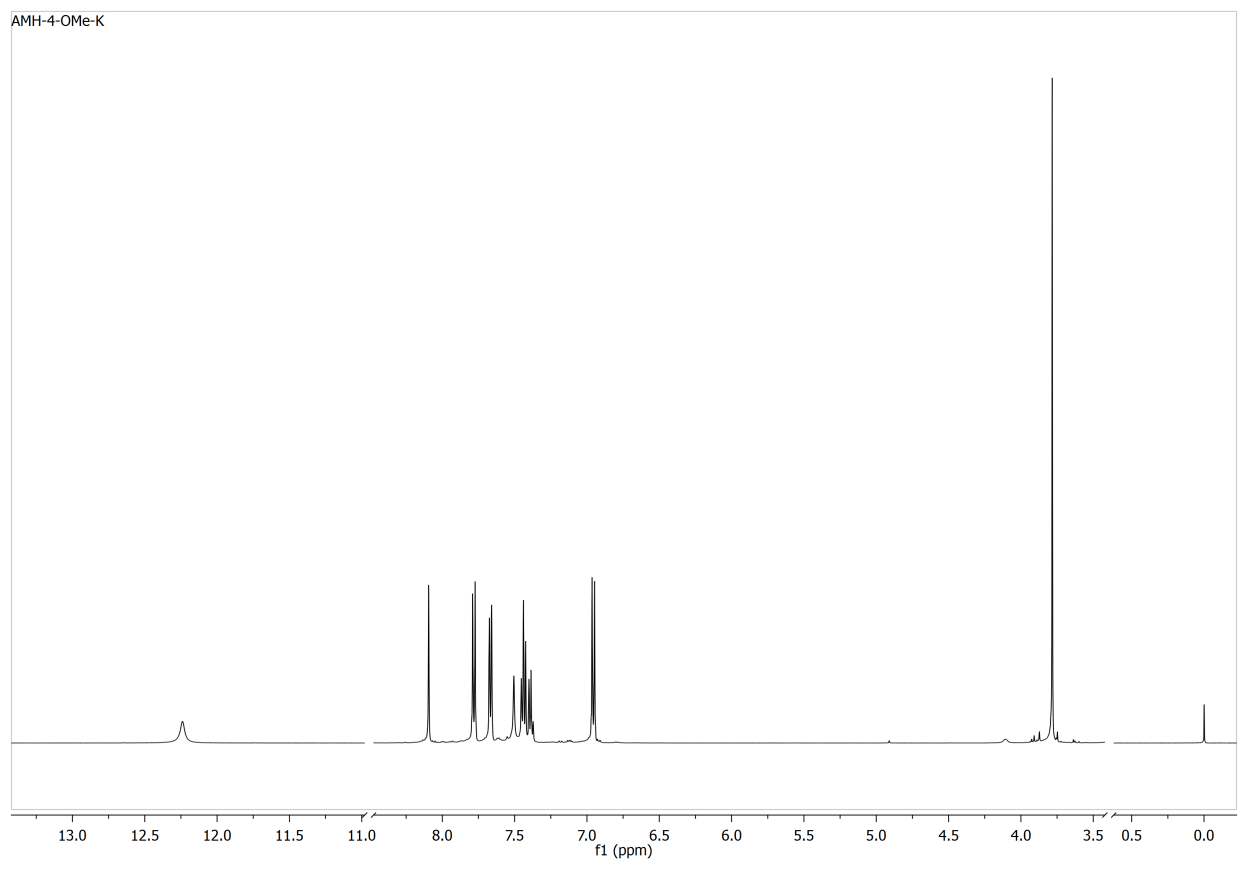
**

**Supplementary Figure S8.** ^1^H NMR spectrum of **1-OMe** in DMSO-*d*_6_.


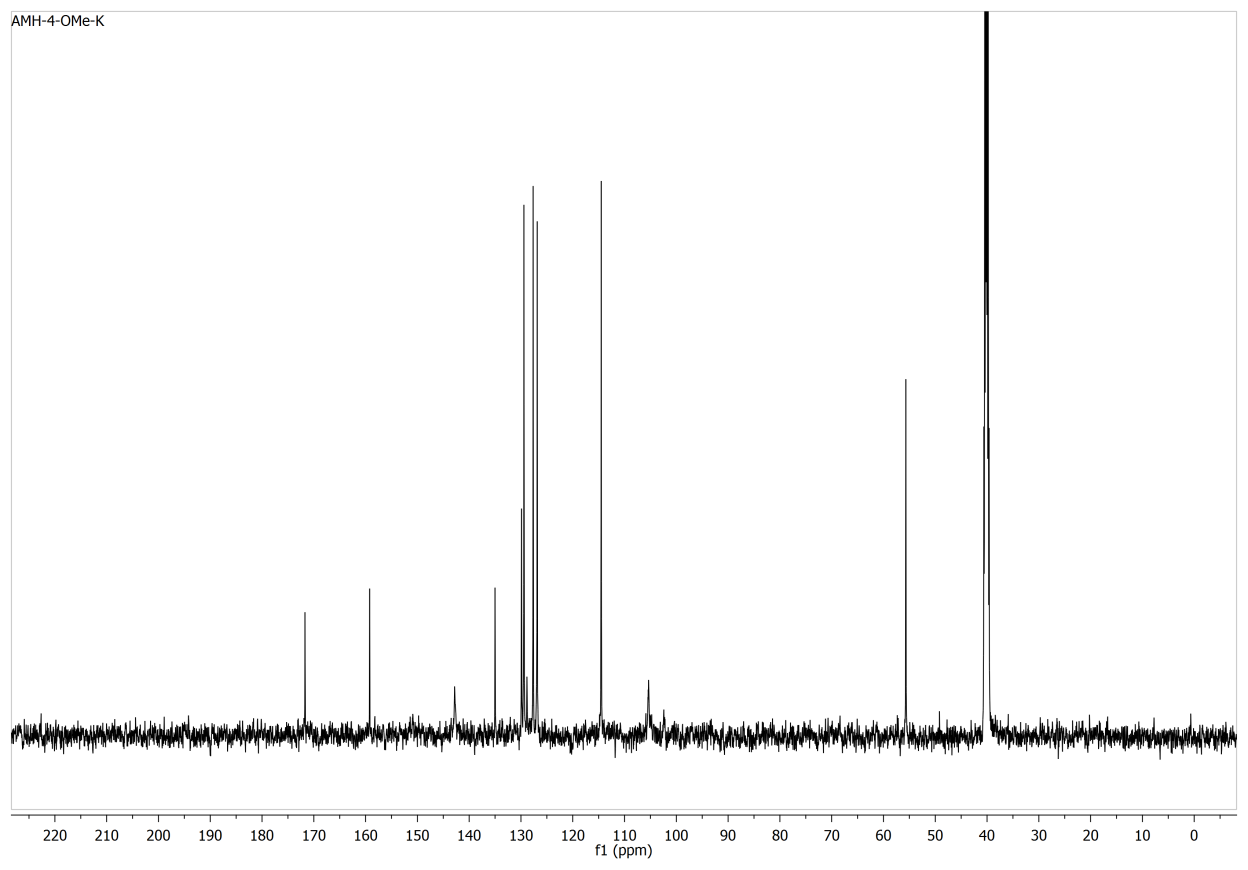


**Supplementary Figure S9.** ^13^C NMR spectrum of **1-OMe** in DMSO-*d*_6_.


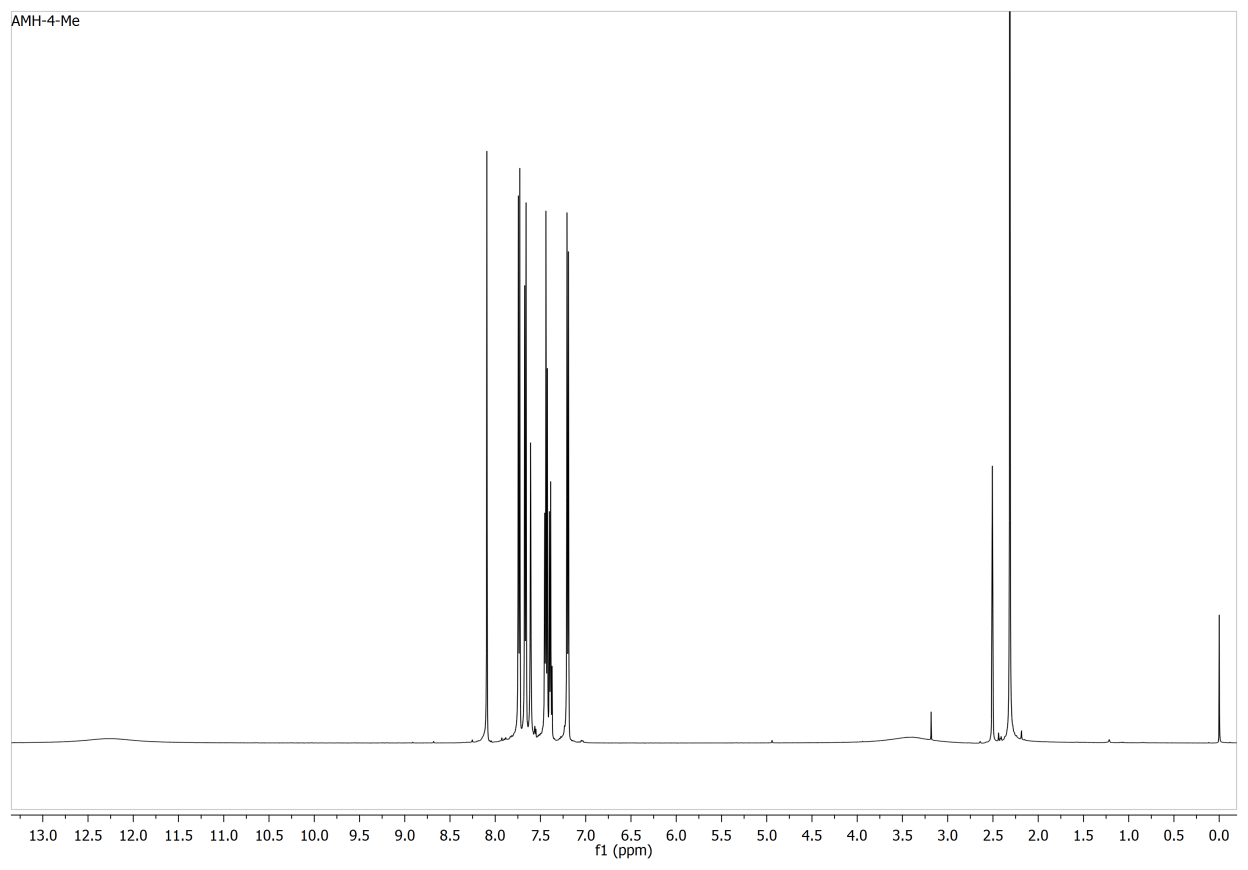


**Supplementary Figure S10.** ^1^H NMR spectrum of **1-Me** in DMSO-*d*_6_.


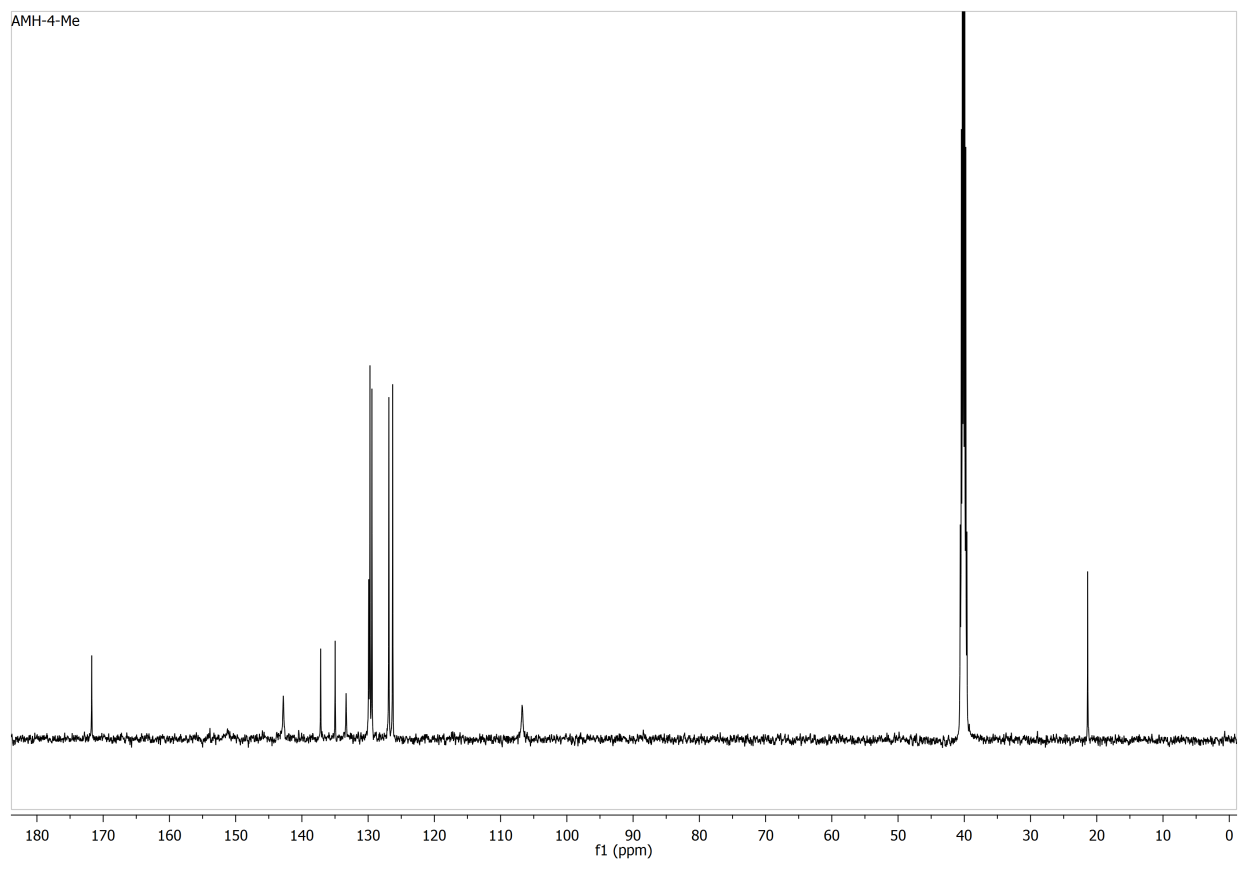


**Supplementary Figure S11.** ^13^C NMR spectrum of **1-Me** in DMSO-*d*_6_.


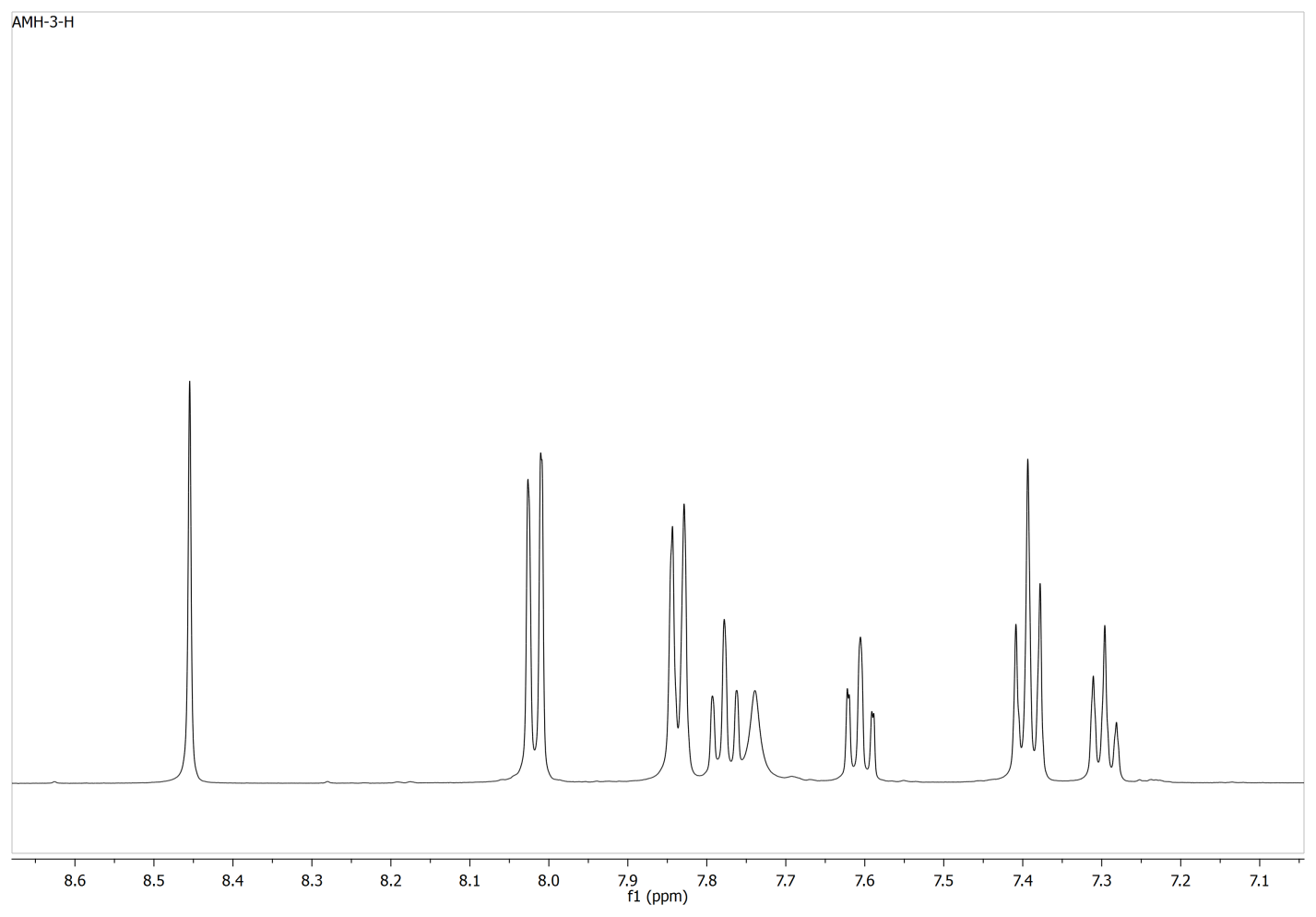


**Supplementary Figure S12.** ^1^H NMR spectrum of **2** in DMSO-*d_6_*.


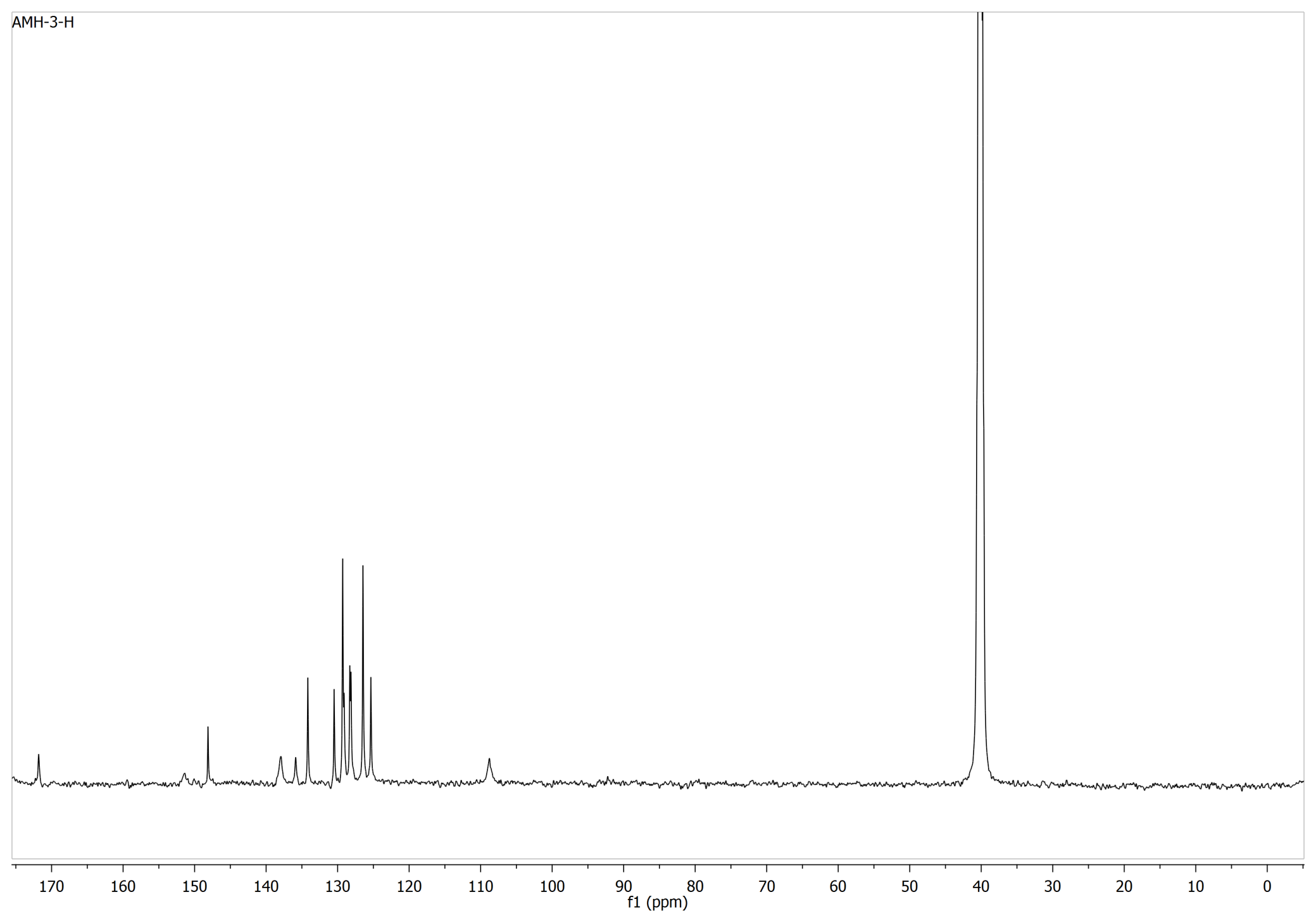


**Supplementary Figure S13.** ^13^C NMR spectrum of **2** in DMSO-*d_6._*


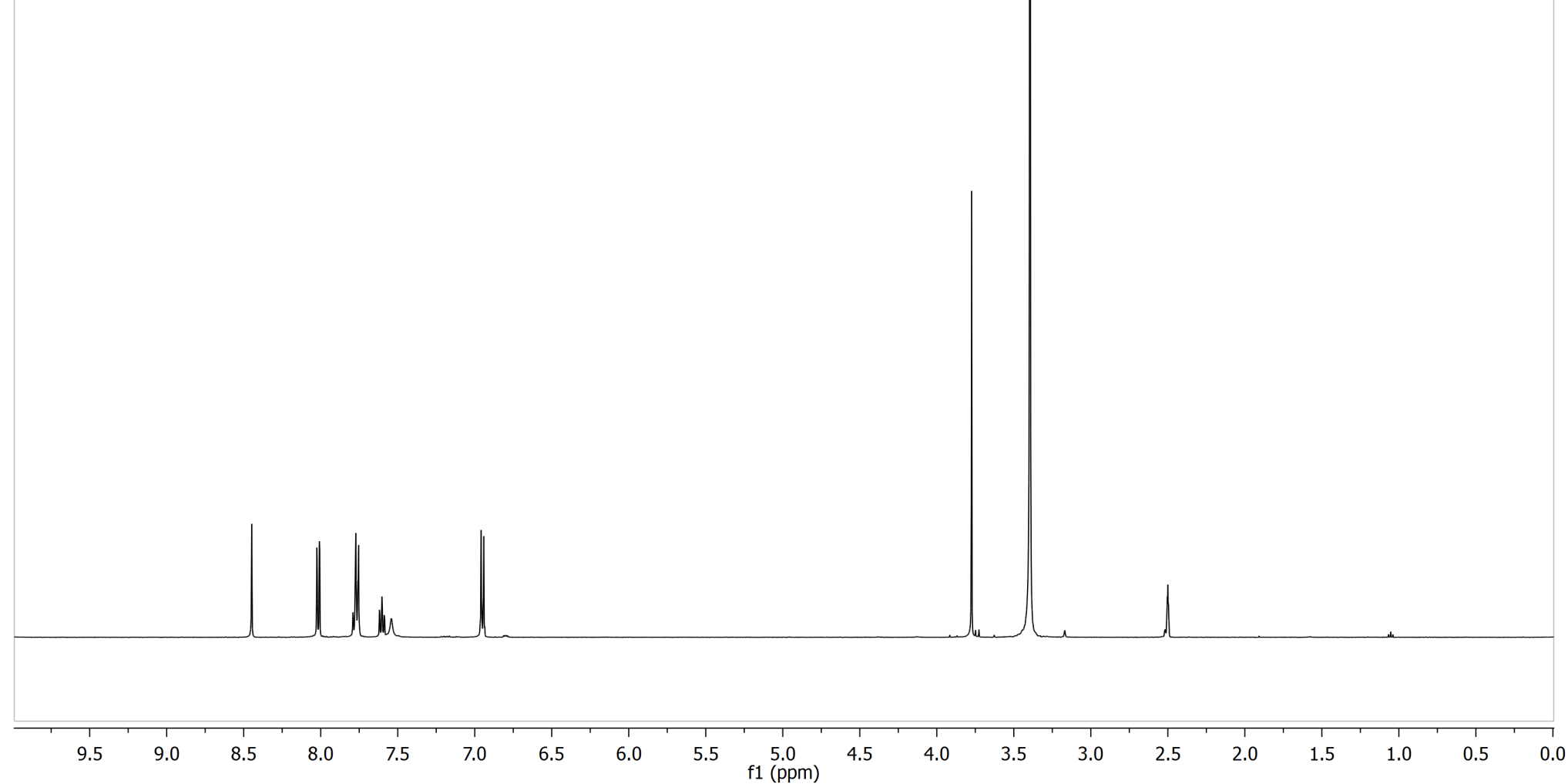


**Supplementary Figure S14.** ^1^H NMR spectrum of **2-OMe** in DMSO-*d_6_*.


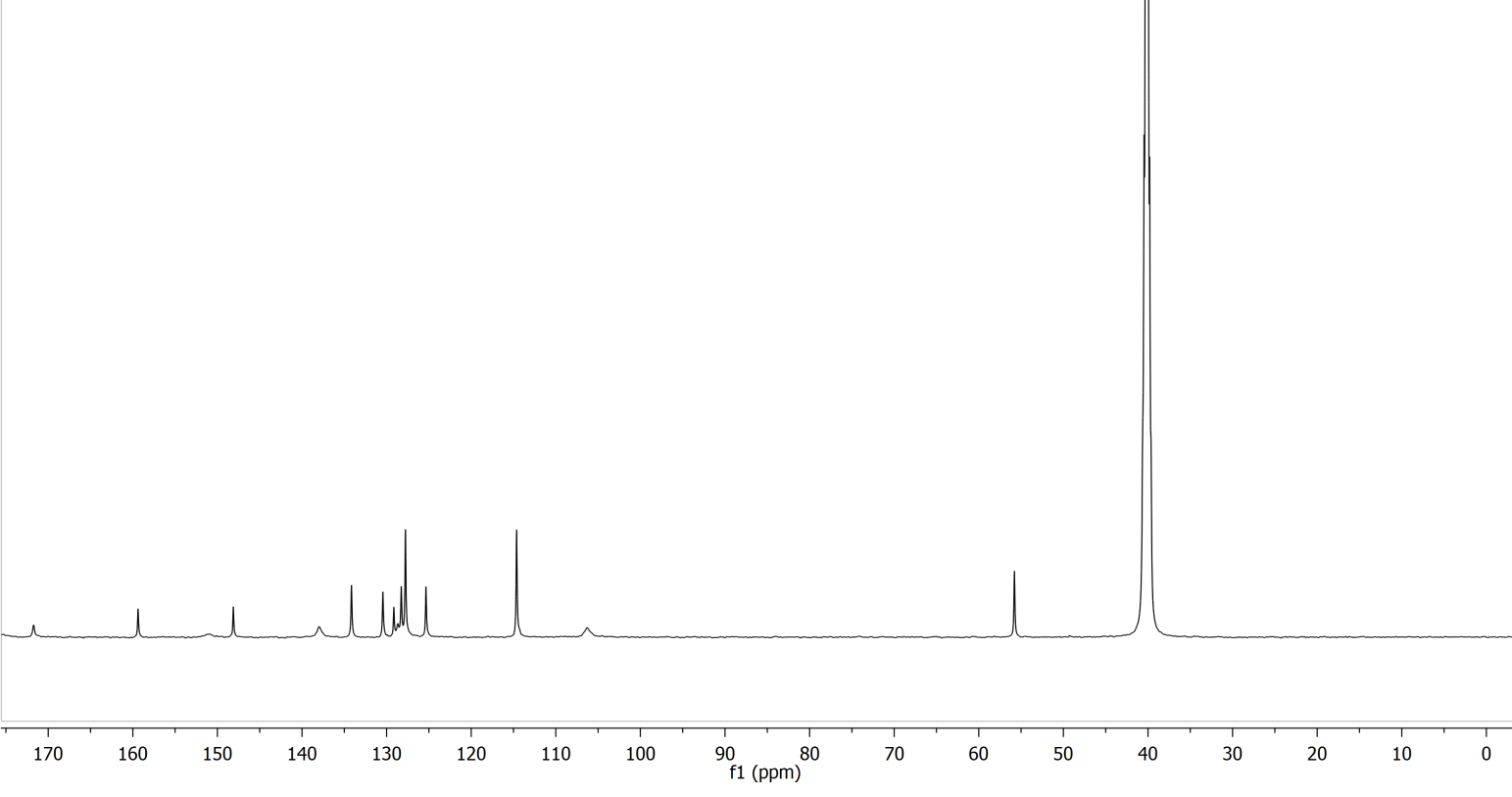


**Supplementary Figure S15.** ^13^C NMR spectrum of **2-OMe** in DMSO-*d_6._*


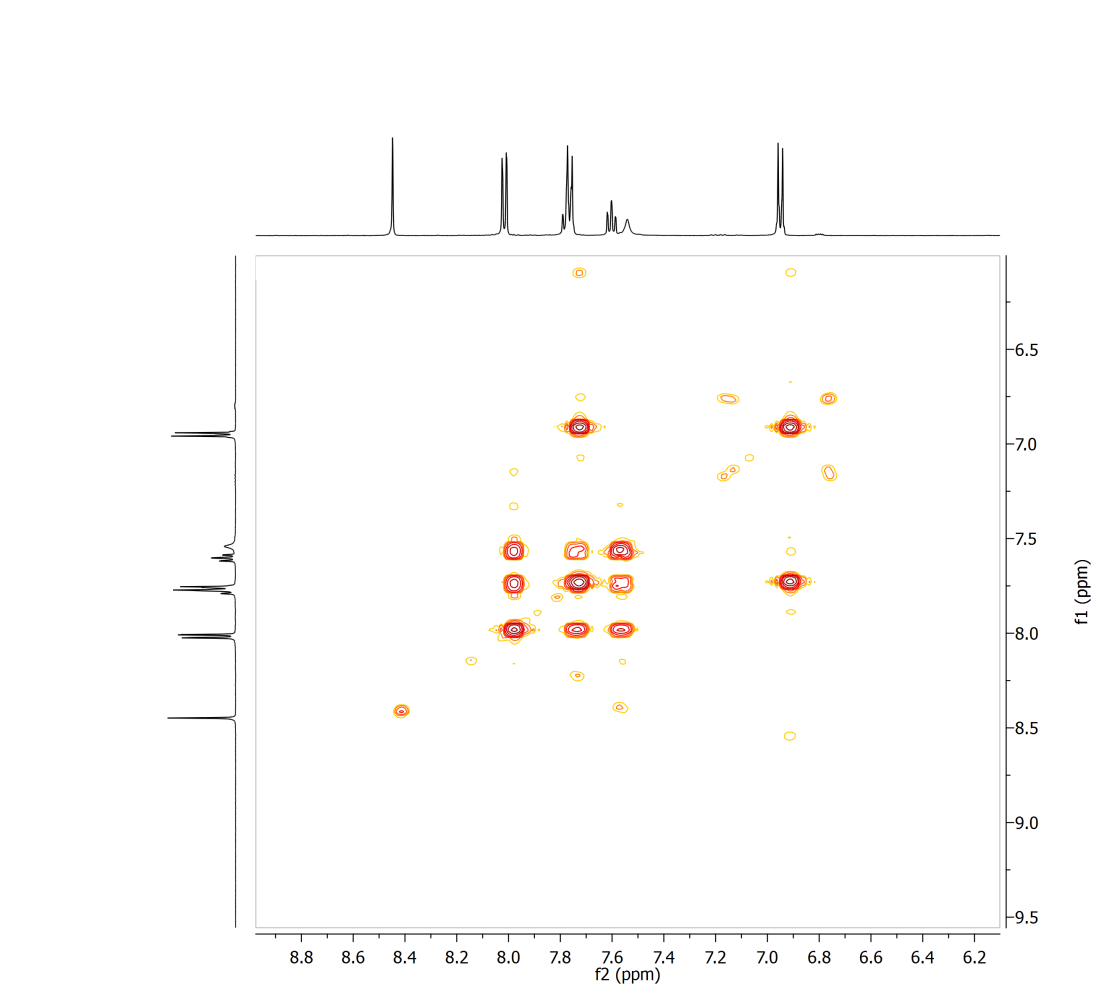


**Supplementary Figure S16.** COSY spectrum of **2-OMe** in DMSO-*d_6_*.


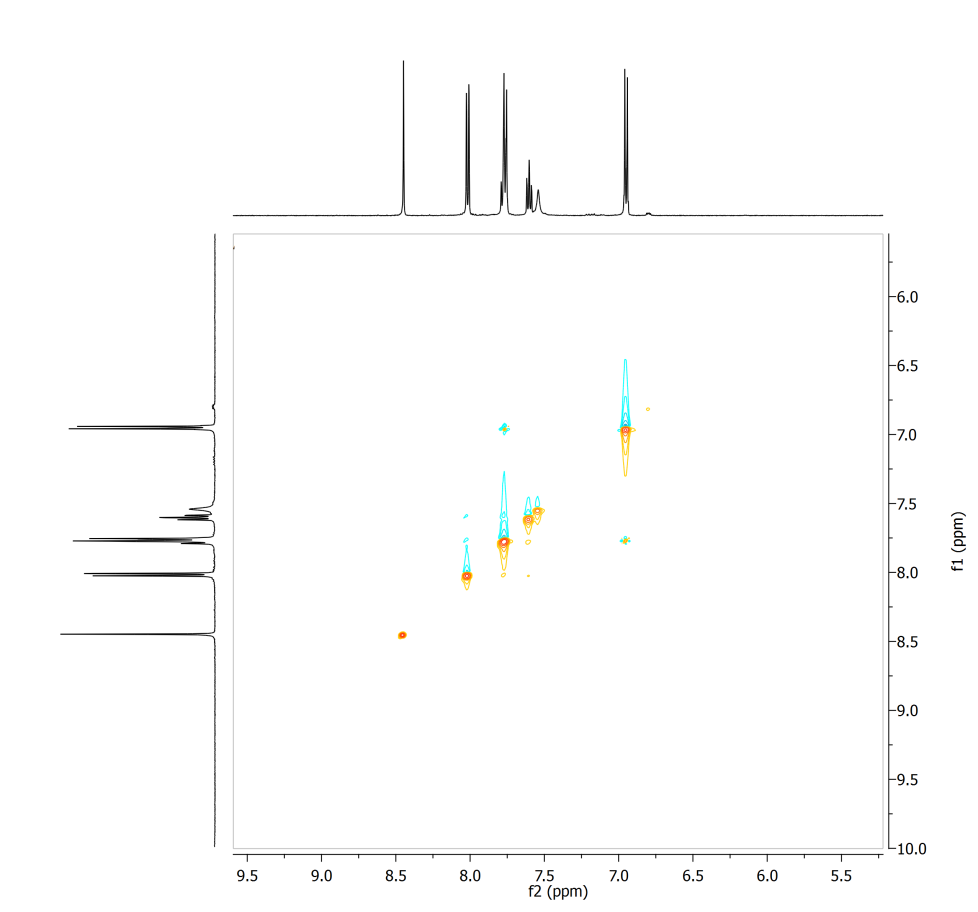


**Supplementary Figure S17.** NOESY spectrum of **2-OMe** in DMSO-*d_6_*.


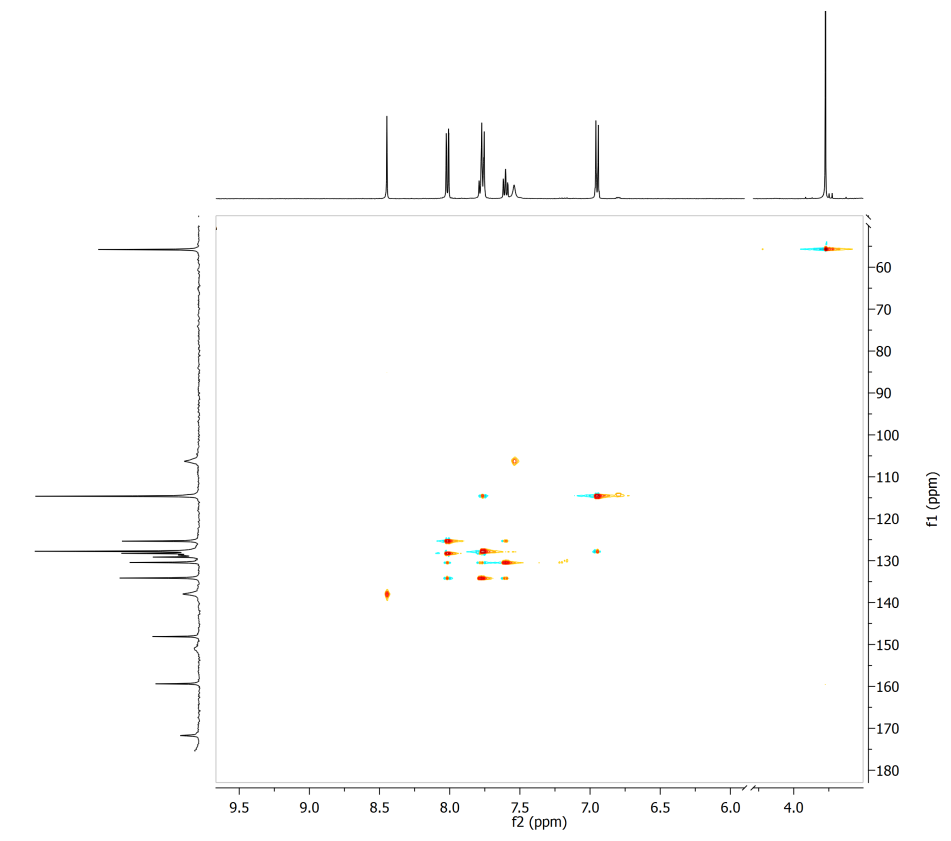


**Supplementary Figure S18.** ^1^H−^13^C HSQC NMR spectrum of **2-OMe** in DMSO-*d_6_*.


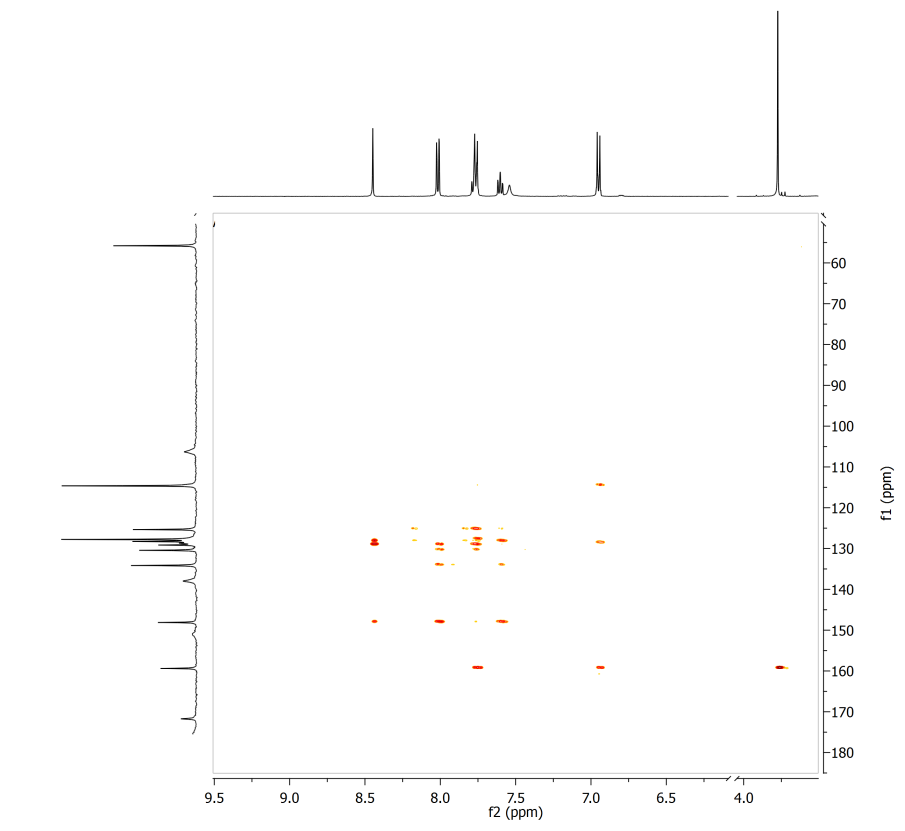


**Supplementary Figure S19.** ^1^H−^13^C HMBC NMR spectrum of **2-OMe** in DMSO-*d_6_*.


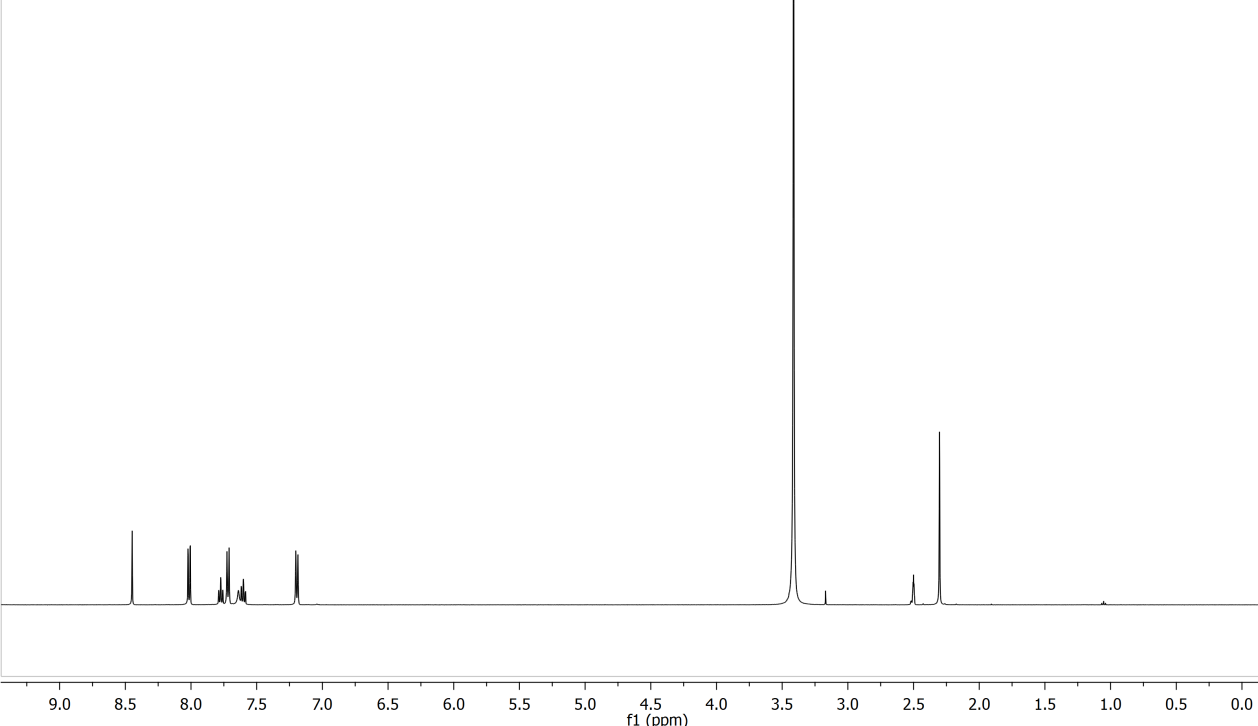


**Supplementary Figure S20.** ^1^H NMR spectrum of **2-Me** in DMSO-*d_6_*.


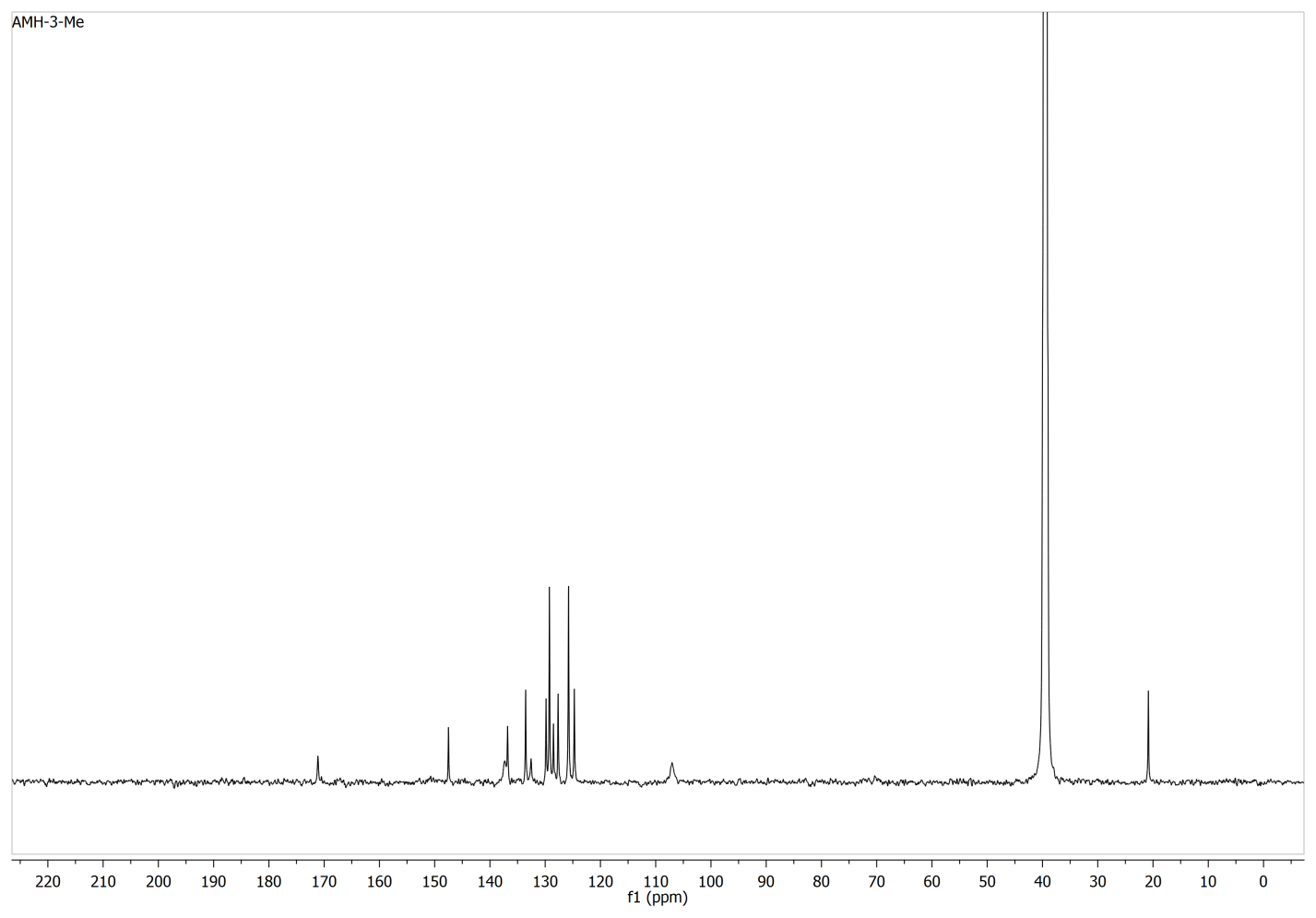


**Supplementary Figure S21.** ^13^C NMR spectrum of **2-Me** in DMSO-*d_6._*


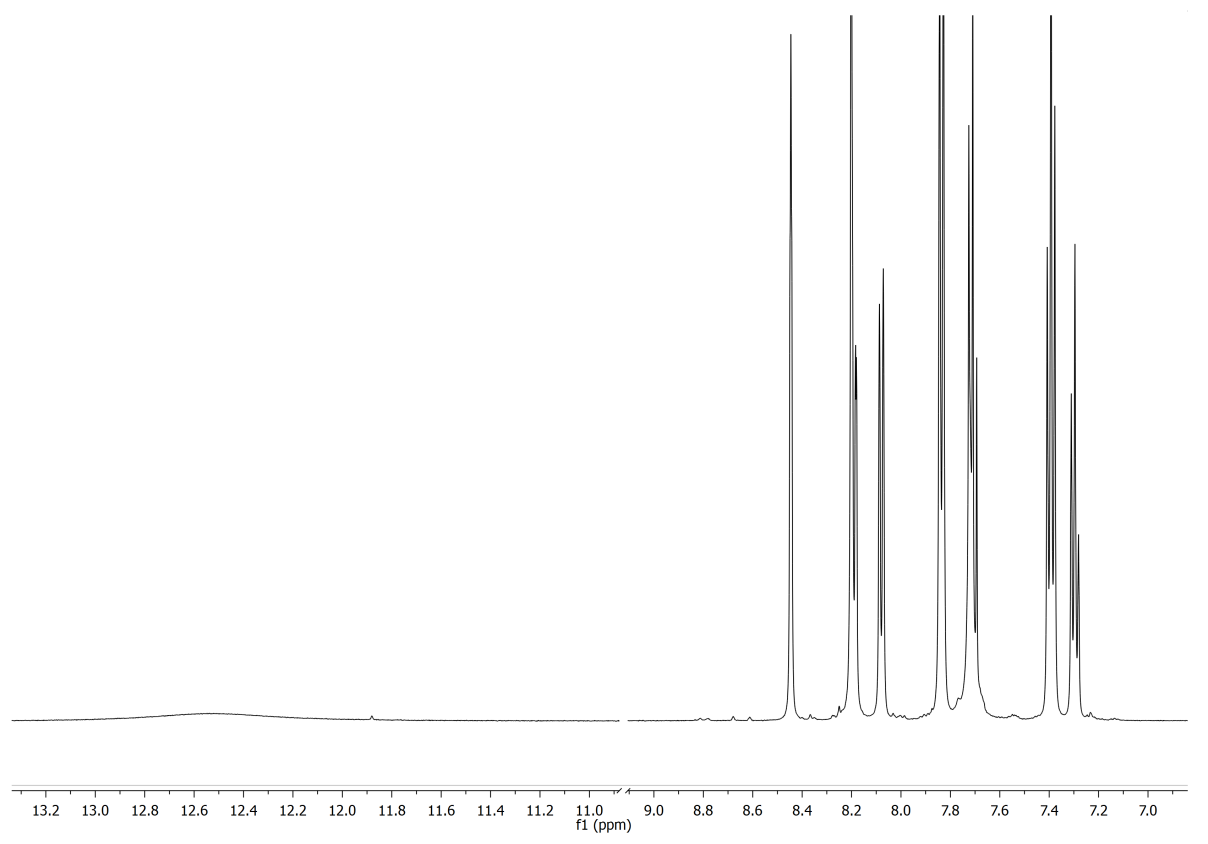


**Supplementary Figure S22.** ^1^H NMR spectrum of **3** in DMSO-*d_6_*.


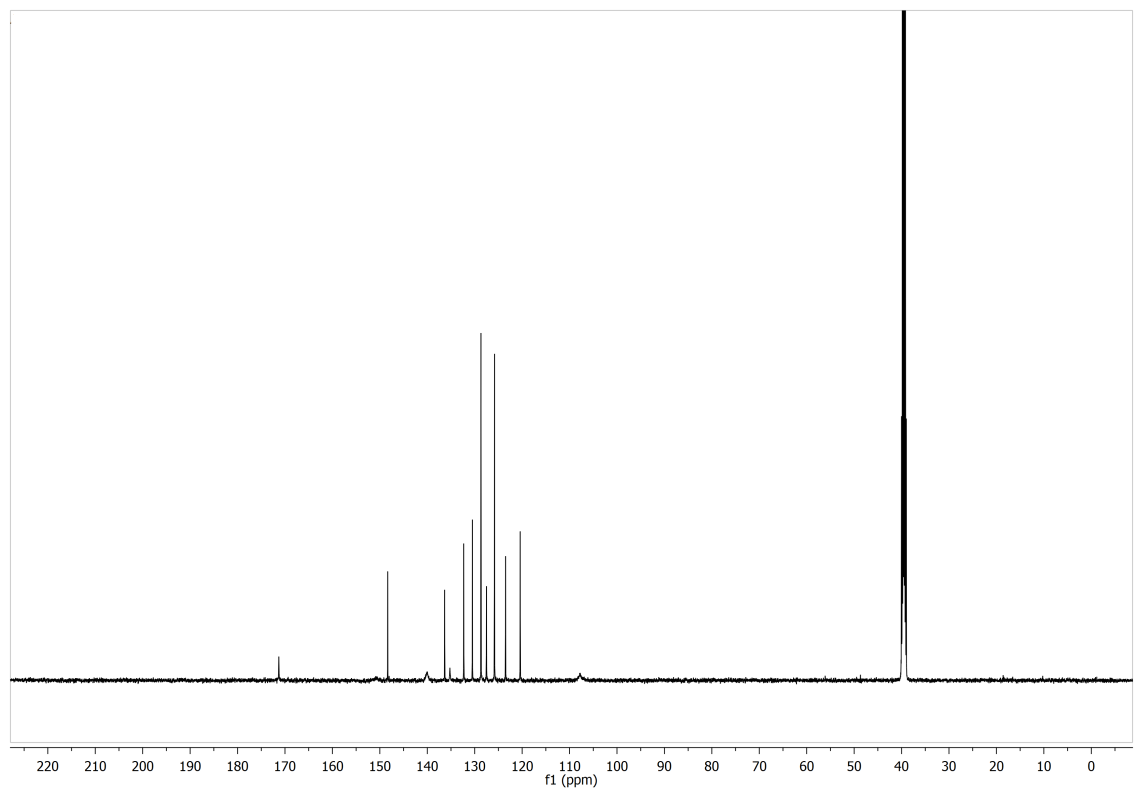


**Supplementary Figure S23.** ^13^C NMR spectrum of **3** in DMSO-*d_6_*.


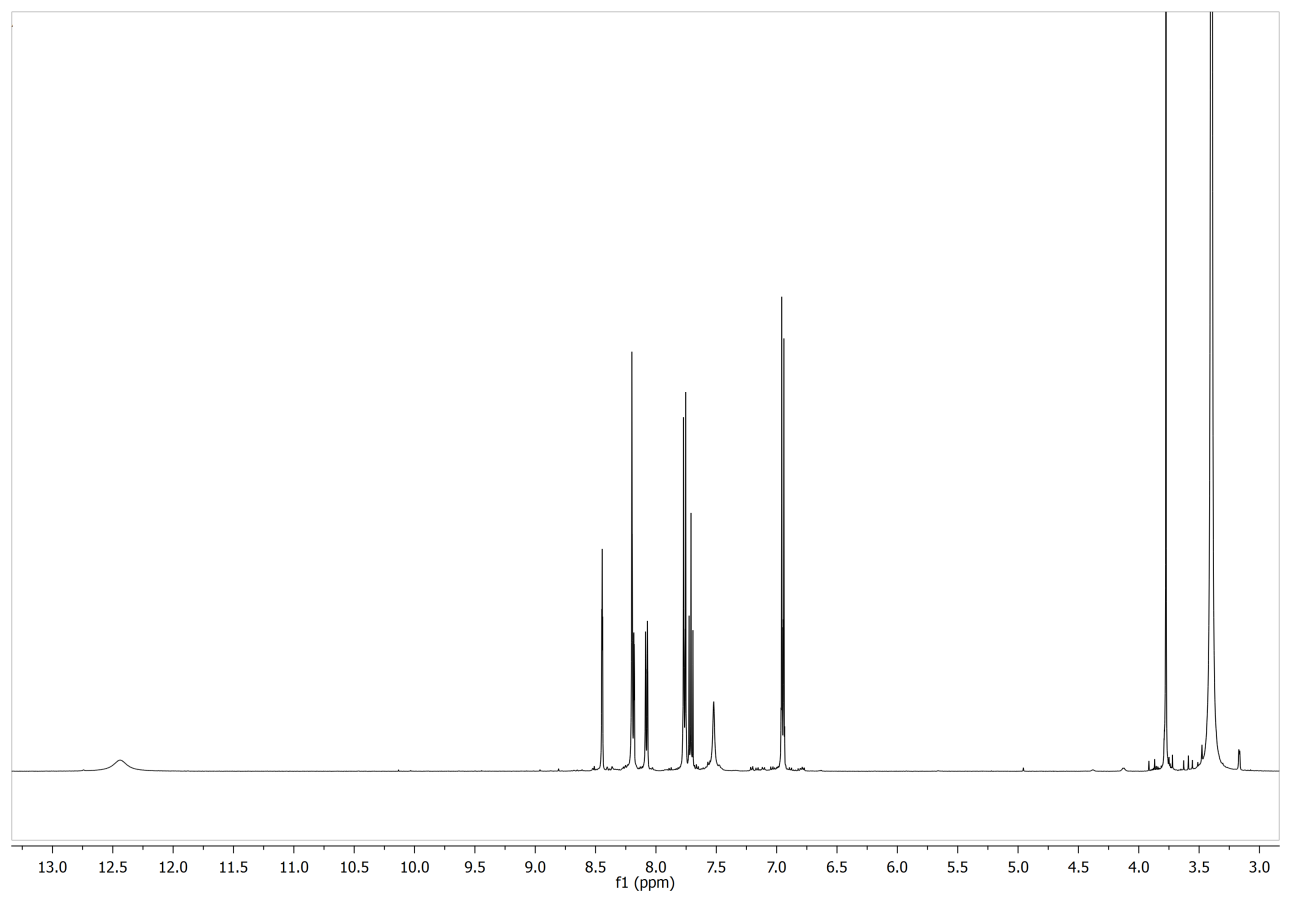


**Supplementary Figure S24.** ^1^H NMR spectrum of **3-OMe** in DMSO-*d_6_.*


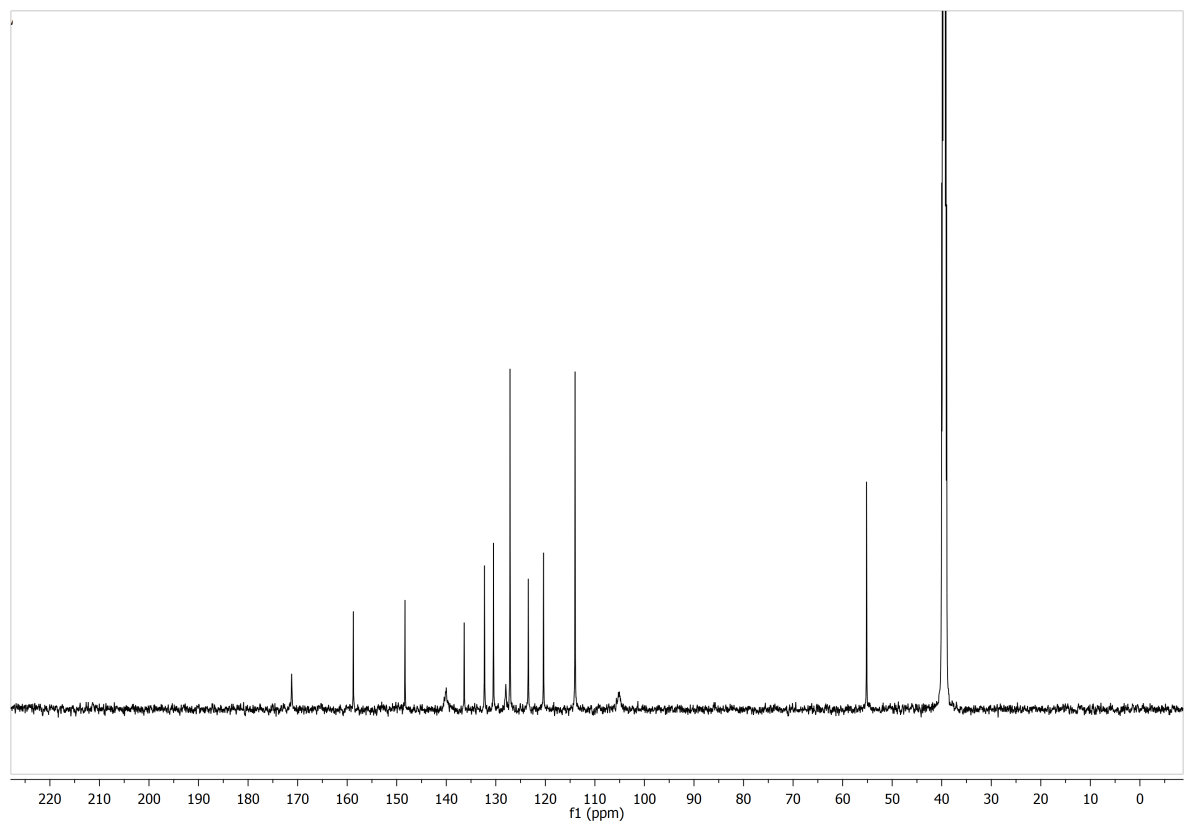


**Supplementary Figure S25.** ^13^C NMR spectrum of **3-OMe** in DMSO-*d_6._*


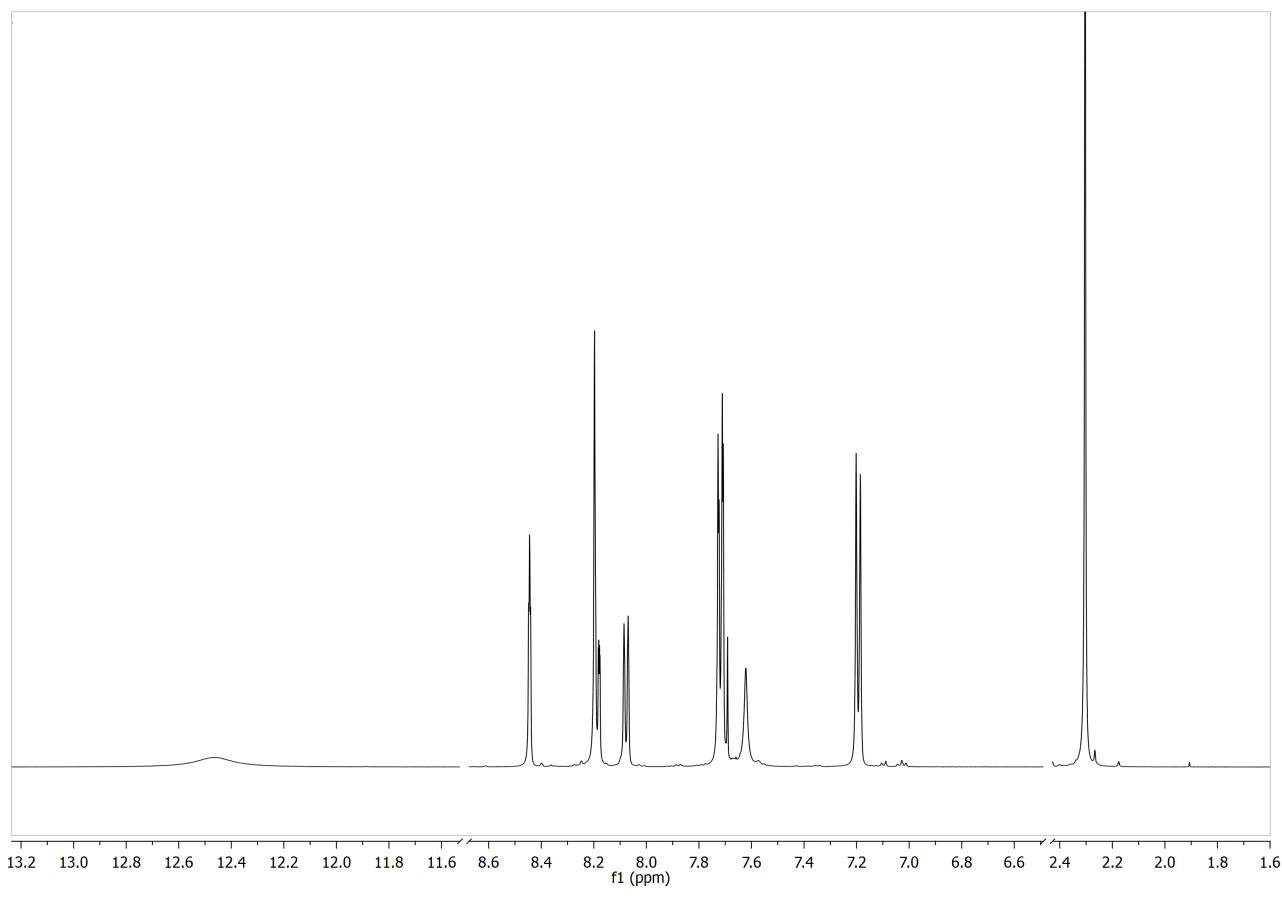


**Supplementary Figure S26.** ^1^H NMR spectrum of **3-Me** in DMSO-*d_6_*.


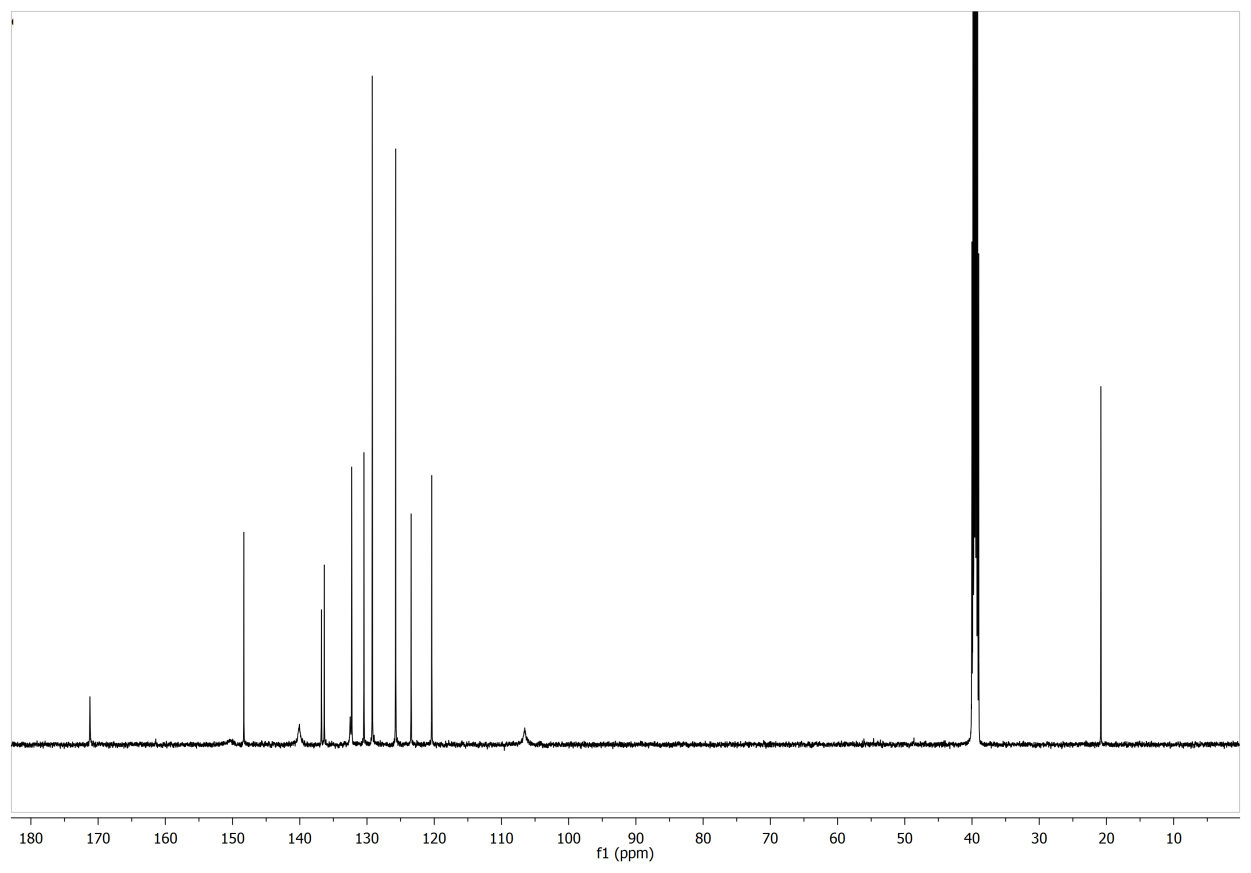


**Supplementary Figure S27.** ^13^C NMR spectrum of **3-Me** in DMSO-*d_6_*.


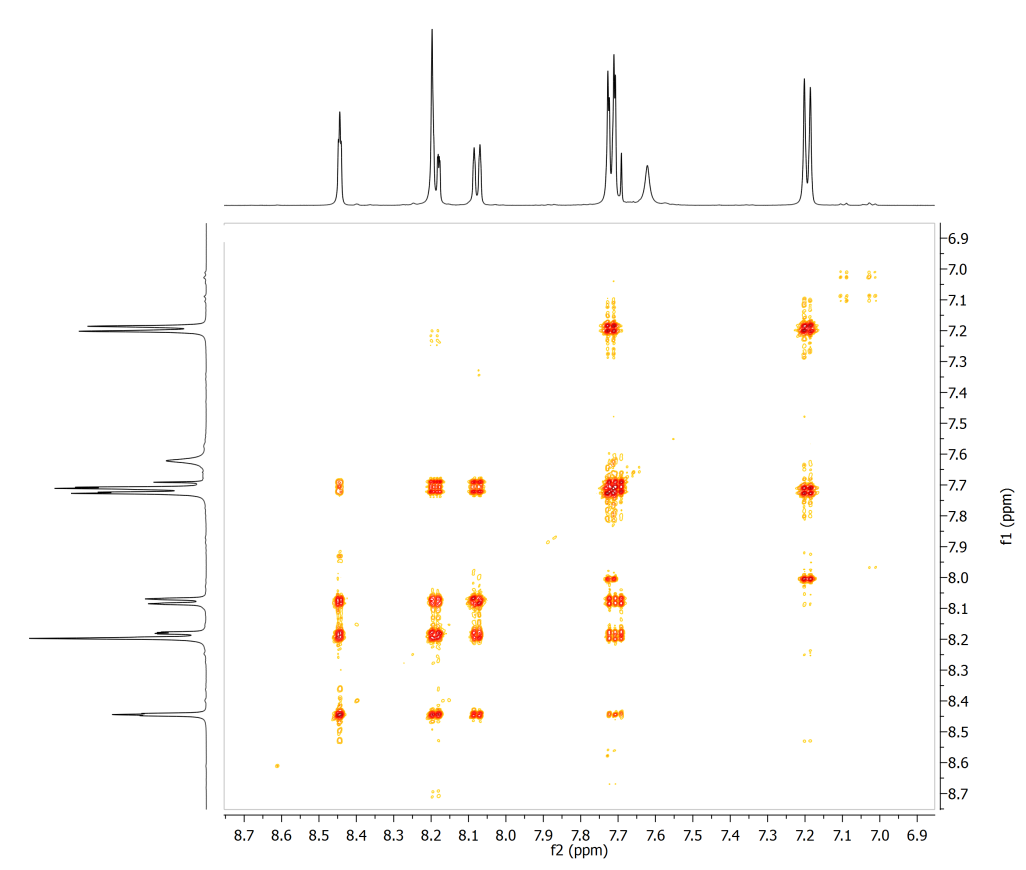


**Supplementary Figure S28.** COSY spectrum of **3-Me** in DMSO-*d_6_*.


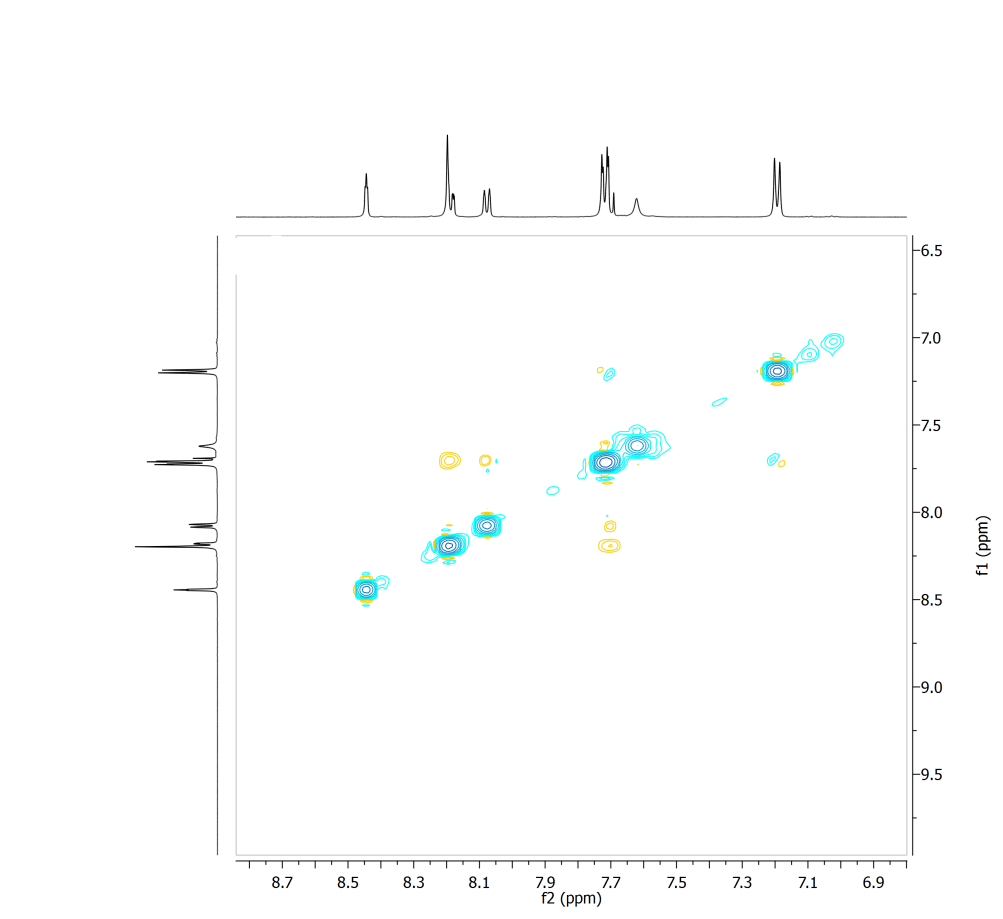


**Supplementary Figure S29.** NOESY spectrum of **3-Me** in DMSO-*d_6_*.


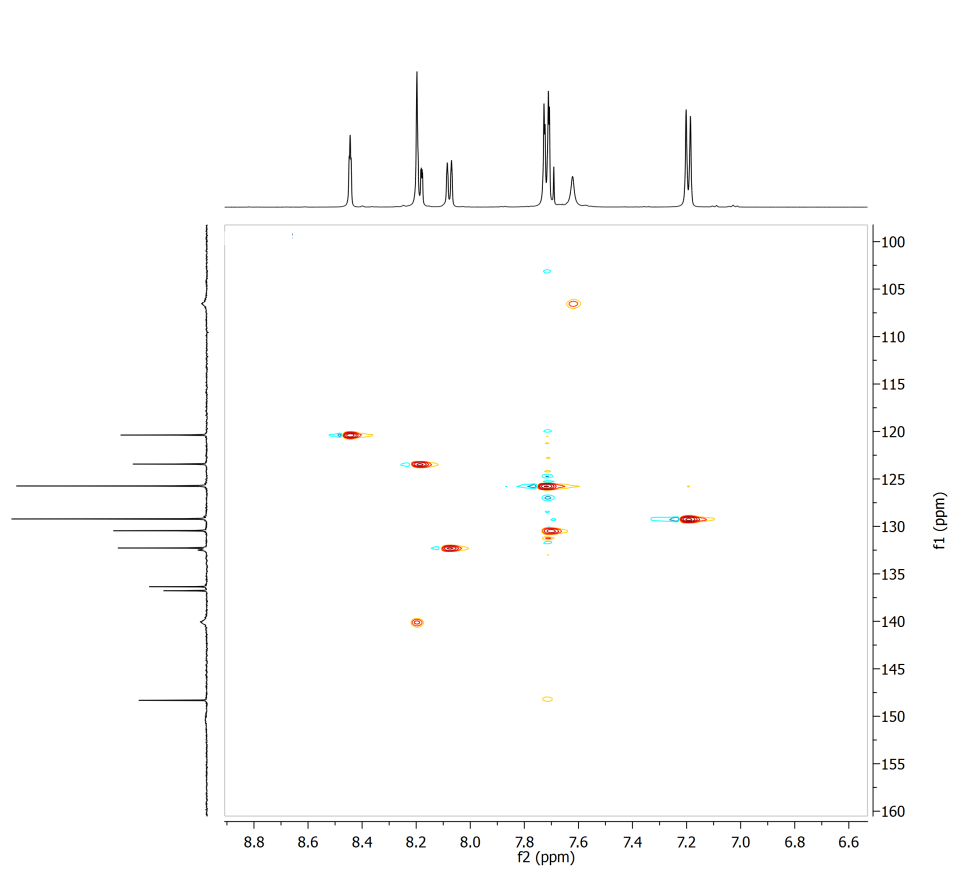


**Supplementary Figure S30.** ^1^H−^13^C HSQC NMR spectrum of **3-Me** in DMSO-*d_6_*.


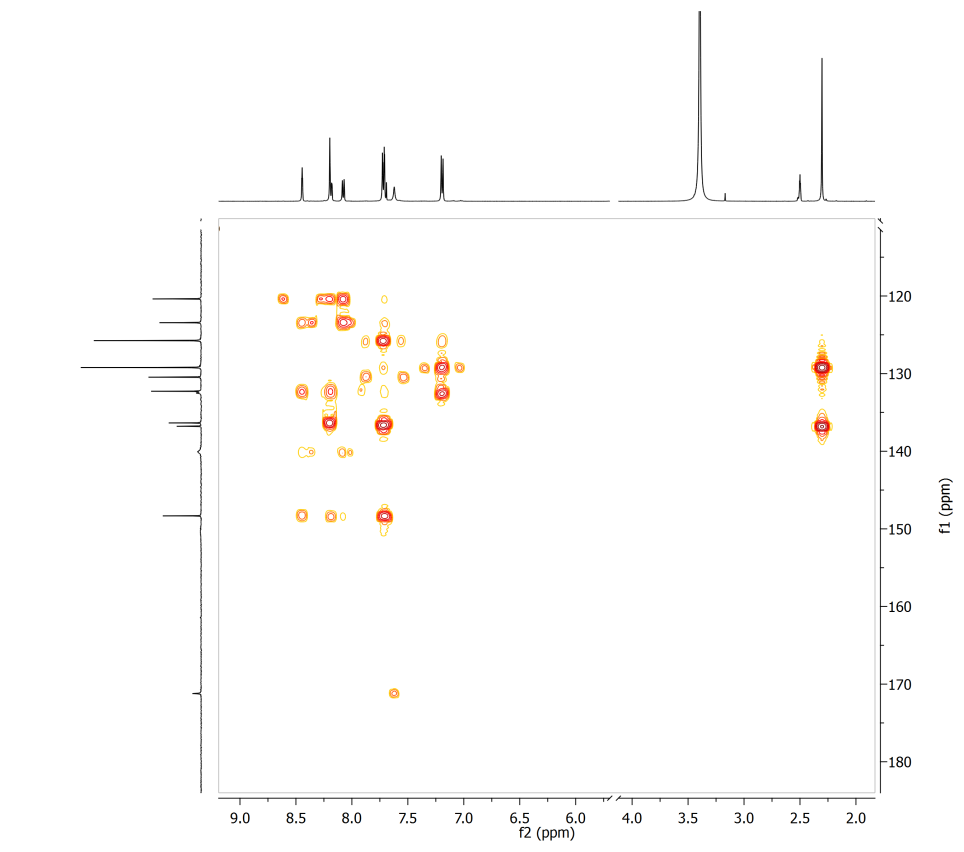


**Supplementary Figure S31.** ^1^H−^13^C HMBC NMR spectrum of **3-Me** in DMSO-*d_6_*.


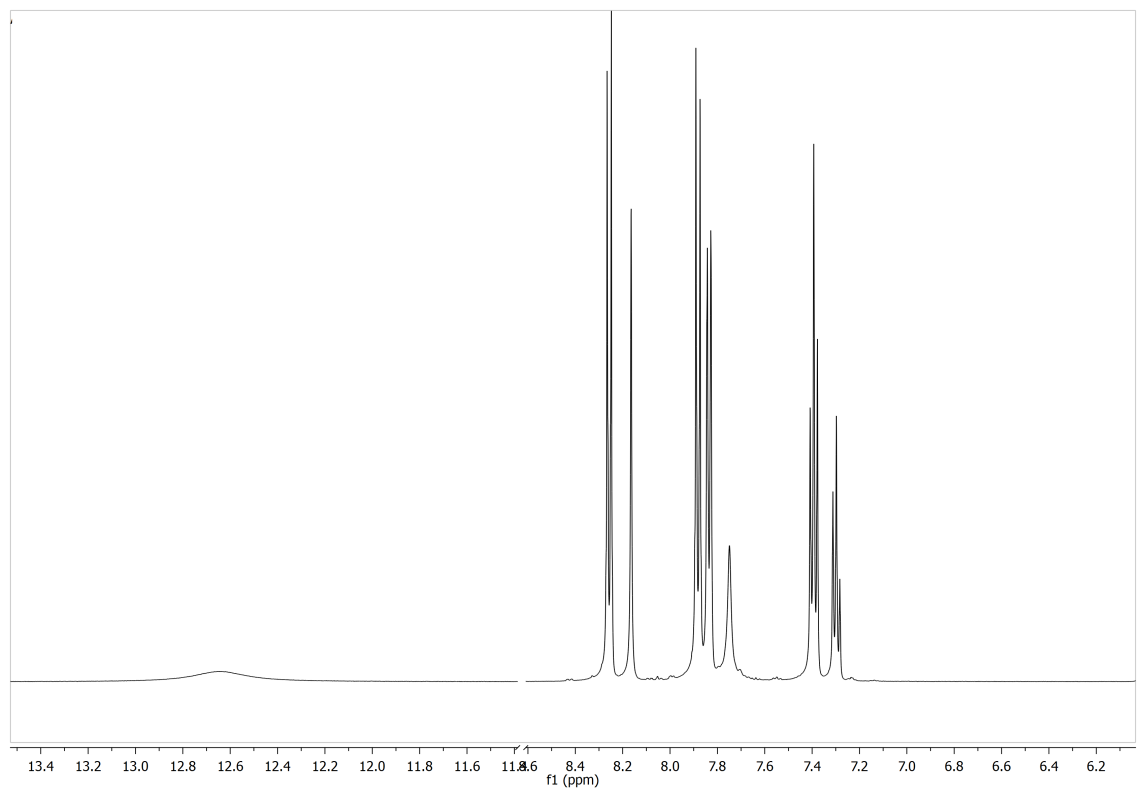


**Supplementary Figure S32.** ^1^H NMR spectrum of **4** in DMSO-*d_6_*.


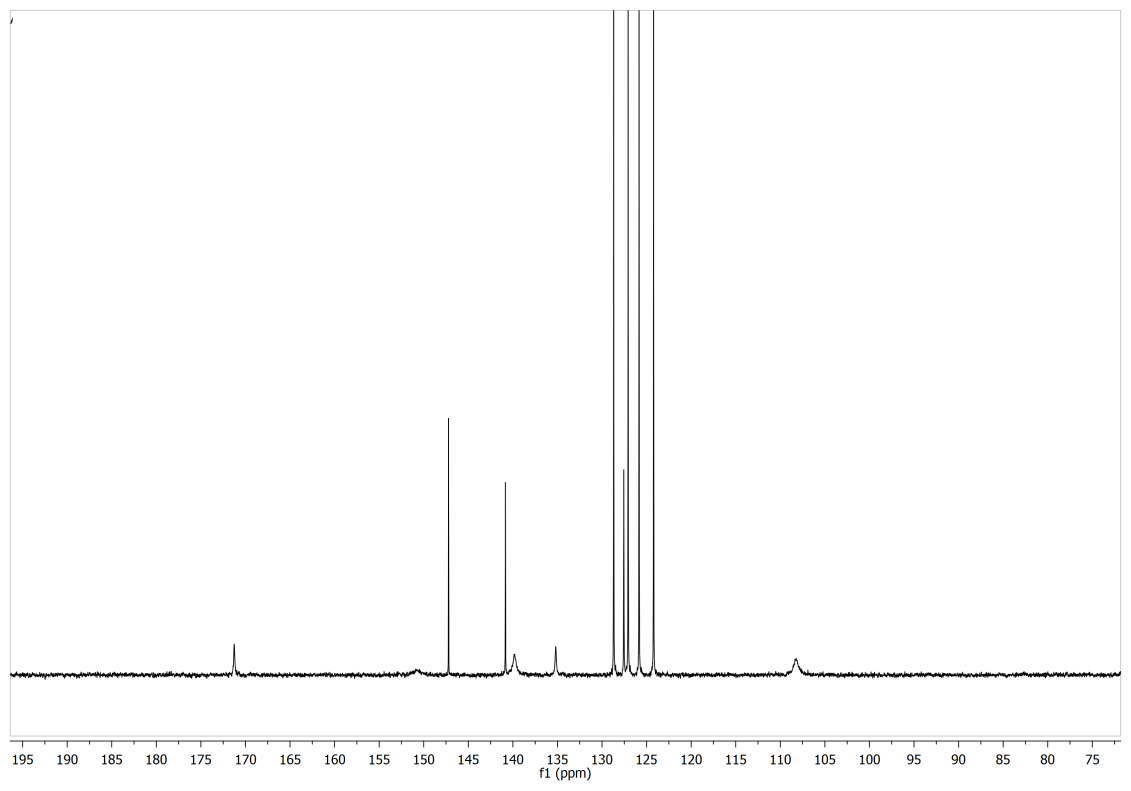


**Supplementary Figure S33.** ^13^C NMR spectrum of **4** in DMSO-*d_6_*.


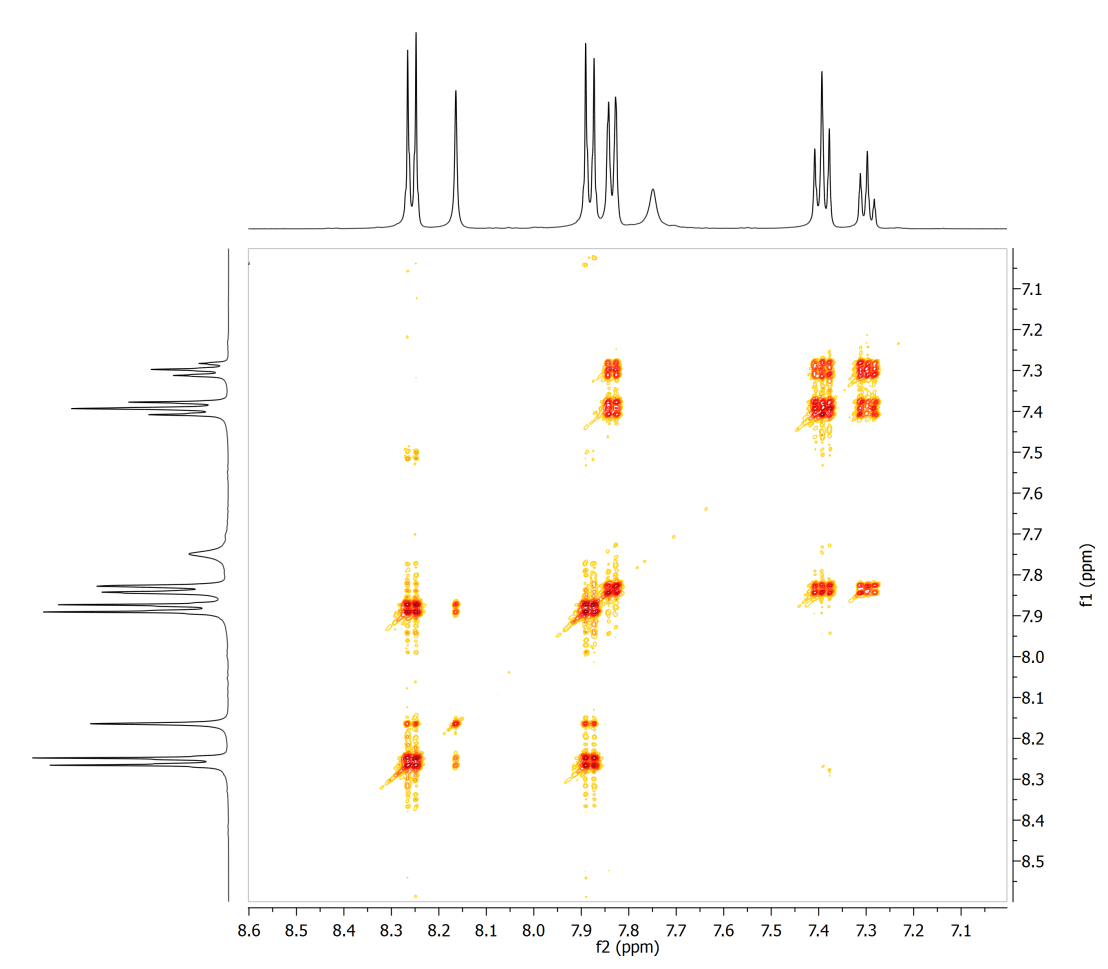


**Supplementary Figure S34.** COSY spectrum of **4** in DMSO-*d_6_*.


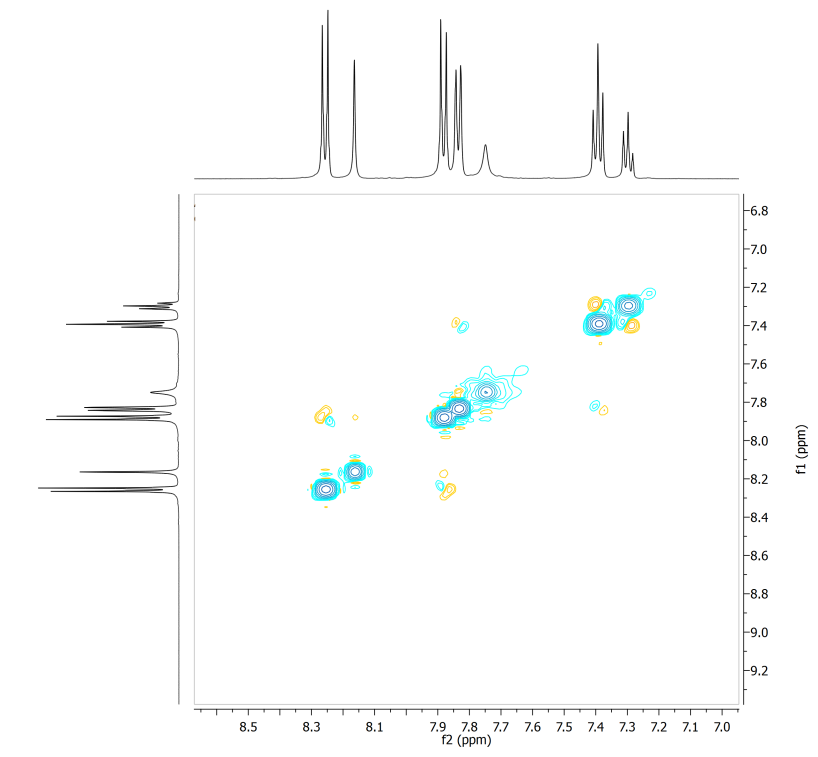


**Supplementary Figure S35.** NOESY spectrum of **4** in DMSO-*d_6_*.


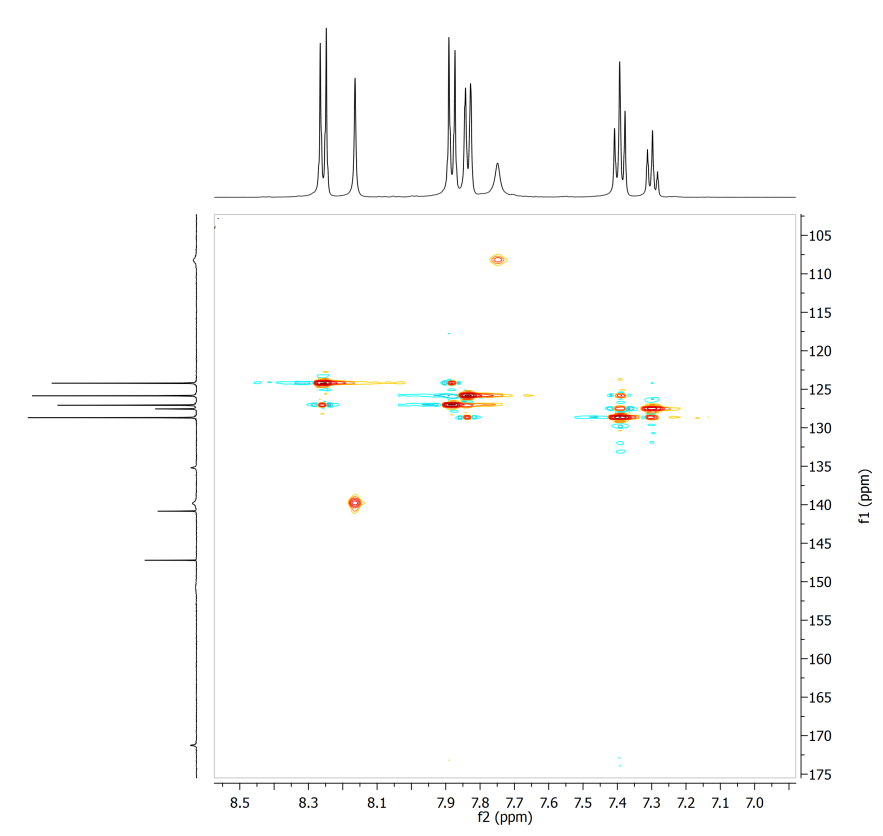


**Supplementary Figure S36.** ^1^H−^13^C HSQC NMR spectrum of **4** in DMSO-*d_6_*.


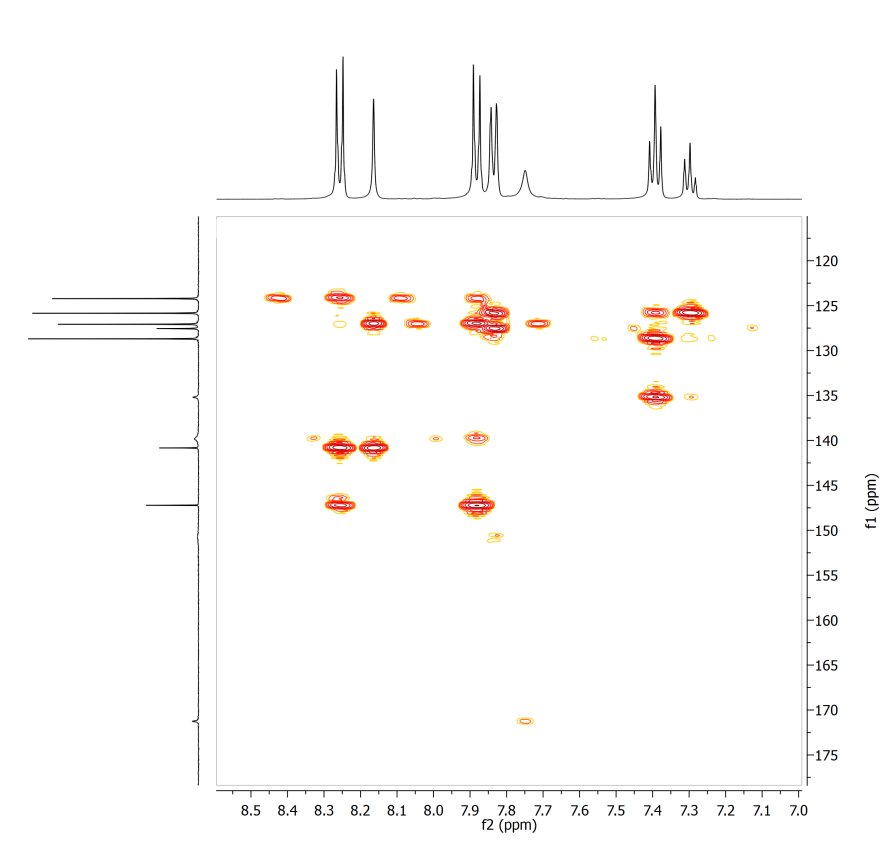


**Supplementary Figure S37.** ^1^H−^13^C HMBC NMR spectrum of **4** in DMSO-*d_6_*.


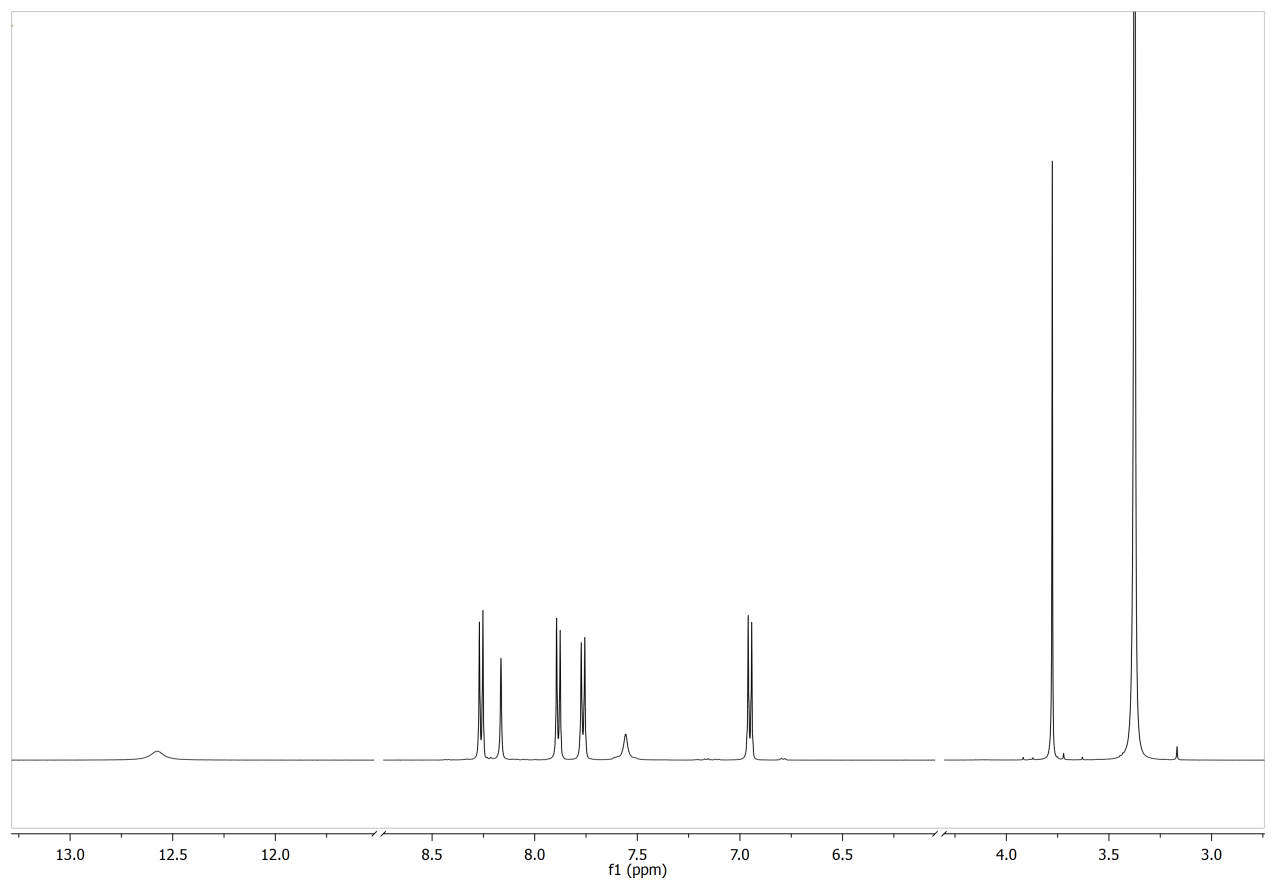


**Supplementary Figure S38.** ^1^H NMR spectrum of **4-OMe** in DMSO-*d_6_*.


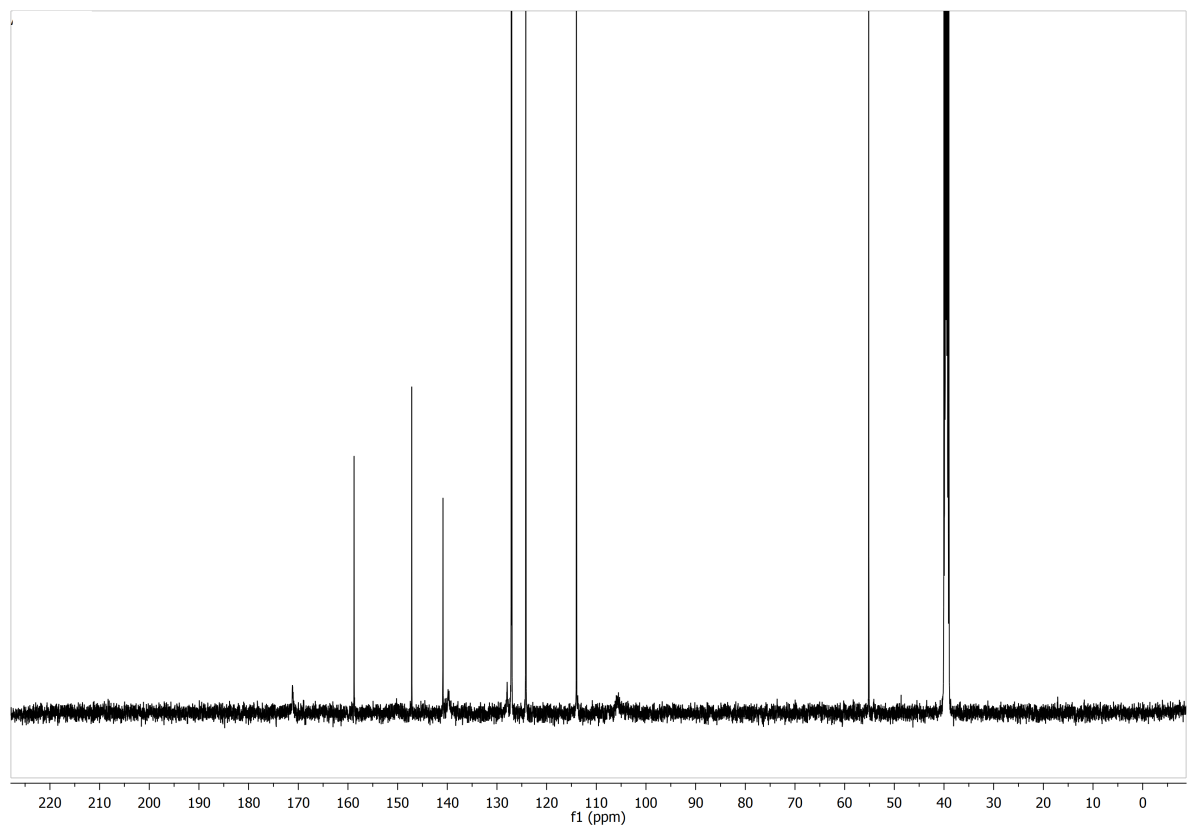


**Supplementary Figure S39.** ^13^C NMR spectrum of **4-OMe** in DMSO-*d_6_*.


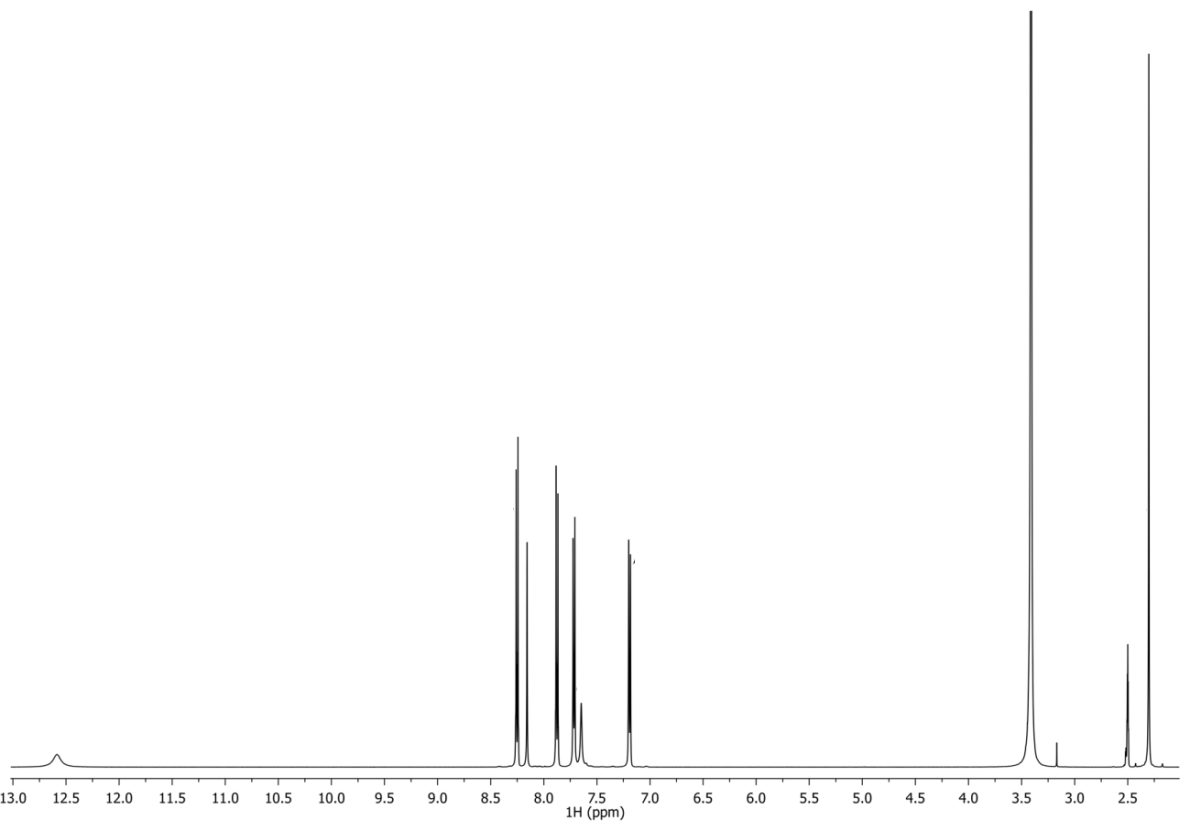


**Supplementary Figure S40.** ^1^H NMR spectrum of **4-Me** in DMSO-*d_6_*.


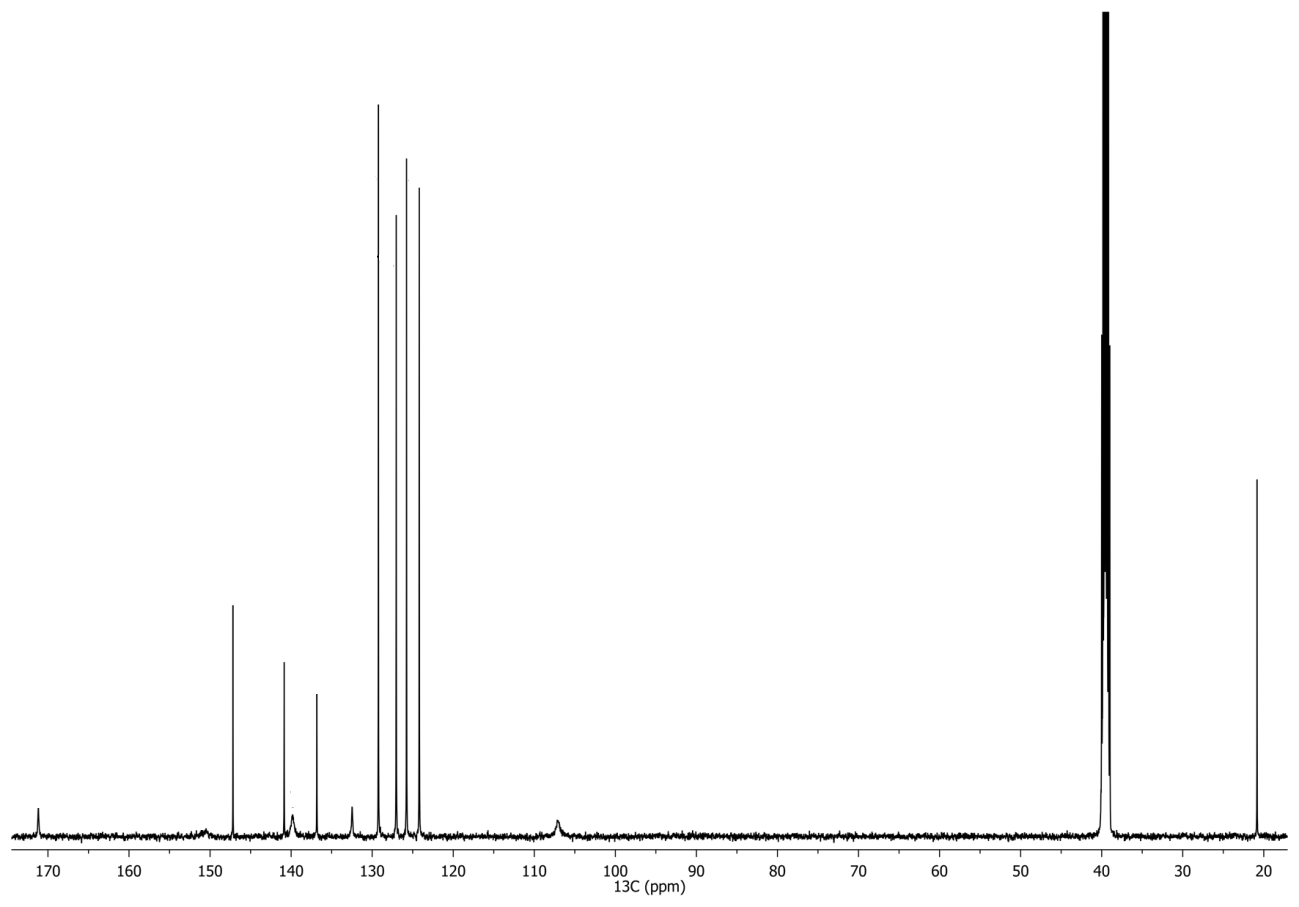


**Supplementary Figure S41.** ^13^C NMR spectrum of **4-Me** in DMSO-*d_6_*.

Supplementary Table S1. Crystallographic data for 4-Me and 4-OMe.

| **Structure** | **4-Me** | **4-OMe** |
| --- | --- | --- |
| *Brutto* formula | C_17_H_14_N_4_O_2_Se | C_17_H_14_N_4_O_3_Se |
| Formula weight (gmol^‒1^) | 385.28 | 401.28 |
| Crystal color and habit | Orange prism | Brown prism |
| Crystal dimensions (mm) | 0.33 × 0.26 × 0.21 | 0.30 × 0.21 × 0.20 |
| Space group | *Pbcn* | *Pbca* |
| *a* (Å) | 11.4924(3) | 11.8767(6) |
| *b* (Å) | 7.9189(4) | 13.5795(7) |
| *c* (Å) | 35.9317(19) | 20.3707(11) |
| *V* (Å^3^) | 3270.0(3) | 3285.4(3) |
| *Z* | 8 | 8 |
| *μ* (Cu*K*_α_) (mm^-1^) | 3.263 | 3.218 |
| Absorption correction | Multi-scan | Multi-scan |
| *F*(000) | 1552 | 1616 |
| *θ* max (°) | 74.000 | 76.014 |
| No. refl. measured | 7934 | 9975 |
| No. refl. unique | 3212 | 3383 |
| No. refl. observed [*I*>2σ(*I*)] | 2562 | 2912 |
| *R*_int_ | 0.0395 | 0.0229 |
| *R*_σ_ | 0.0601 | 0.0315 |
| Parameters | 227 | 239 |
| *R*_1_ [*I*>2σ(*I*)] | 0.0461 | 0.0319 |
| *wR*_2_, all | 0.1414 | 0.0964 |
| *S* | 1.054 | 1.044 |
| *ρ*_max_, *ρ*_min_ (eÅ^‒3^) | 0.46, ‒0.68 | 0.25, ‒0.34 |

Supplementary Table S2. Angles between the selenazole ring least square plane and phenyl rings least square planes.

| Se1‒C8‒C9‒N10‒C11 | **4-Me** (º) | **4-OMe** (º) |
| --- | --- | --- |
| C2‒C3‒C4‒C5‒C6‒C7 | 30.18 (15) | 4.94 (10) |
| C15‒C16‒C17‒C18‒C19‒C20 | 24.86 (15) | 6.03 (11) |
